# A wearable platform for closed-loop stimulation and recording of single-neuron and local field potential activity in freely moving humans

**DOI:** 10.1038/s41593-023-01260-4

**Published:** 2023-02-20

**Authors:** Uros Topalovic, Sam Barclay, Chenkai Ling, Ahmed Alzuhair, Wenhao Yu, Vahagn Hokhikyan, Hariprasad Chandrakumar, Dejan Rozgic, Wenlong Jiang, Sina Basir-Kazeruni, Sabrina L. Maoz, Cory S. Inman, Matthias Stangl, Jay Gill, Ausaf Bari, Aria Fallah, Dawn Eliashiv, Nader Pouratian, Itzhak Fried, Nanthia Suthana, Dejan Markovic

**Affiliations:** 1 Department of Electrical and Computer Engineering, University of California, Los Angeles, Los Angeles, CA USA; 2 Department of Psychiatry and Biobehavioral Sciences, Jane and Terry Semel Institute for Neuroscience and Human Behavior, University of California, Los Angeles, Los Angeles, CA USA; 3 Department of Electrical Engineering, King Saud University, Riyadh, Saudi Arabia; 4 Department of Bioengineering, University of California, Los Angeles, Los Angeles, CA USA; 5 Medical Scientist Training Program, David Geffen School of Medicine, University of California, Los Angeles, Los Angeles, CA USA; 6 Department of Psychology, University of Utah, Salt Lake City, UT USA; 7 Department of Neurosurgery, David Geffen School of Medicine, University of California, Los Angeles, Los Angeles, CA USA; 8 Department of Neurology, University of California, Los Angeles, Los Angeles, CA USA; 9 Department of Neurological Surgery, UT Southwestern Medical Center, Dallas, TX USA; 10 Department of Psychology, University of California, Los Angeles, Los Angeles, CA USA

**Keywords:** Extracellular recording, Learning algorithms, Lab-on-a-chip, Long-term memory

## Abstract

Advances in technologies that can record and stimulate deep brain activity in humans have led to impactful discoveries within the field of neuroscience and contributed to the development of novel therapies for neurological and psychiatric disorders. Further progress, however, has been hindered by device limitations in that recording of single-neuron activity during freely moving behaviors in humans has not been possible. Additionally, implantable neurostimulation devices, currently approved for human use, have limited stimulation programmability and restricted full-duplex bidirectional capability. In this study, we developed a wearable bidirectional closed-loop neuromodulation system (Neuro-stack) and used it to record single-neuron and local field potential activity during stationary and ambulatory behavior in humans. Together with a highly flexible and customizable stimulation capability, the Neuro-stack provides an opportunity to investigate the neurophysiological basis of disease, develop improved responsive neuromodulation therapies, explore brain function during naturalistic behaviors in humans and, consequently, bridge decades of neuroscientific findings across species.

## Abstract (web summary)

The Neuro-stack is a wearable platform for recording from single neurons in freely moving humans. It also allows for personalized stimulation during real-time decoding of neural activity.

## Main

Understanding brain function and its relation to cognition and behavior requires the integration of multiple levels of inquiry, ranging from the examination of single cells all the way up to the probing of human experience under naturalistic conditions. One major barrier that separates these approaches is the inability to record single-neuron activity during naturalistic behaviors in humans, which frequently involve full-body locomotion as well as twitches, gestures and actions of the face and hands. Single-neuron studies of freely moving behaviors are currently exclusively done in animals (for example, rodents)^1,2^. Although single-neuron studies in humans have yielded unique insights into memory, perception, decision-making as well as pathologies such as Parkinson’s disease (PD) and epilepsy (for review, see refs. ^3–6^), they have been solely done in immobile participants. Thus, major gaps remain between understanding findings from neuroscience studies in animals to those in humans. Determining the single-neuron mechanisms of naturalistic behavior in individuals who can declare their thoughts and imaginations, interact socially, move freely and navigate real-world environments would present an unprecedented opportunity in the field of systems neuroscience.

In parallel with progress in neuroscience, the medical field has seen a considerable increase in the use of therapies delivered through implanted neural devices to treat and evaluate abnormal brain activity in patients with brain disorders^7–12^. However, current clinical implantable devices do not allow single-neuron recording or extensive customization of stimulation parameters (for example, pulse shape and precise timing with respect to ongoing neural activity), capabilities that would markedly expand the types of research questions that could be investigated. Furthermore, developing accurate animal models of human brain disorders and reproducing associated impairments/symptoms in laboratory settings remain major challenges. Recordings of single-neuron activity in patients with brain disorders, however, especially under naturalistic settings, would provide a unique window into the neural mechanisms of brain pathology, symptoms and treatment response and would lead to more personalized and effective therapies^3,4^. Although several closed-loop neuromodulation therapies using intracranial electroencephalographic (iEEG) recordings are effective, a substantial portion of patients still do not respond to treatment. Furthermore, given temporal relationships between single-unit spiking and ongoing oscillatory activity^13–17^, closed-loop neuromodulation therapies may benefit from personalization during a period when both single-unit and local field potential (LFP) activity is available. Finally, an additional impediment in optimizing closed-loop neuromodulation treatments is the lack of a customizable bidirectional interface with a larger dynamic input range that can record multi-channel single-unit and LFP activity during temporally precise phase-locked stimulation (PLS).

Intracranial neurophysiological studies, using micro-electrodes in patients with epilepsy or PD, can record LFPs and single-unit activity; however, research participants must be tethered to large equipment and remain immobile. There are two possibilities for studies to leverage these clinical opportunities. The first is to use existing research equipment (for example, Blackrock Microsystems (https://blackrockneurotech.com/research), Neuralynx (https://neuralynx.com), Nihon Kohden (EEG-1200 EEG system, https://us.nihonkohden.com/products/eeg-1200) and Ripple Neuro (Custom Neuroscience Research Tools, https://rippleneuro.com)), which is bulky and expensive (up to ~$200,000), with immobile participants who participate in voluntary research studies while hospitalized. Intracranial stimulation studies are similarly done bedside, primarily using open-loop stimulation^18–24^, although recent studies have explored the use of closed-loop stimulation^25–29^. The second option is to use FDA-approved, commercially available devices already implanted in thousands of individuals to treat epilepsy or movement disorders (for example, Neuropace RNS System^30^ and Medtronic Percept^31^). These chronically implanted devices offer research participants mobility at the expense of using larger electrodes and fewer channels (usually four) that cannot record single-unit activity. Other devices (for example, Medtronic Summit RC+S^32–34^) allow for 16-channel iEEG recording at higher sampling rates (but no single units) and exist only in a handful of patients with an FDA investigational device exemption (IDE), limiting their widespread use by the scientific community. These closed-loop implantable neural technologies also have limited full-duplex ability, where multi-channel PLS during single-unit and LFP recording is not possible. Although research studies using existing systems have given rise to several impactful discoveries^30,34–37^, the possibility of devices one day recording from single neurons, delivering customizable closed-loop stimulation, would provide unparalleled opportunities for first-in-human scientific discovery and development of more effective medical therapies for brain disorders.

Here we present a potential technological pathway toward more advanced implantable technologies with the development of a miniaturized bidirectional neuromodulation external device, Neuro-stack. The Neuro-stack can simultaneously record up to 128-channel monopolar or bipolar (from 256 macro-recording contacts) iEEG and 32-channel monopolar (from 32 micro-recording contacts) single-unit/LFP activity during ambulatory behaviors in humans who have macro-electrodes and micro-electrodes implanted for clinical reasons. Moreover, the Neuro-stack can deliver customizable closed-loop multi-channel (up to 32 simultaneous) stimulation where parameters such as pulse shape, frequency, amplitude, pulse width, inter-pulse width, polarity, channel selection and timing (for example, for PLS) are configurable.

We include data acquired using the Neuro-stack showing single-unit, LFP and iEEG activity recorded in 12 participants who had depth electrodes implanted for epilepsy evaluation. In one participant, we used the Neuro-stack to perform binary prediction of memory performance in real time (69% F1 score) using neural activity recorded from medial temporal lobe (MTL) regions. We also demonstrate the Neuro-stack’s ability to record single-neuron activity during walking behavior and deliver customized stimulation. These capabilities could be useful for future studies investigating the neural mechanisms underlying naturalistic behaviors in humans and developing novel closed-loop neuromodulation therapies for patients with brain disorders that will be effective in real-world settings.

## Results

**Fig. 1:**
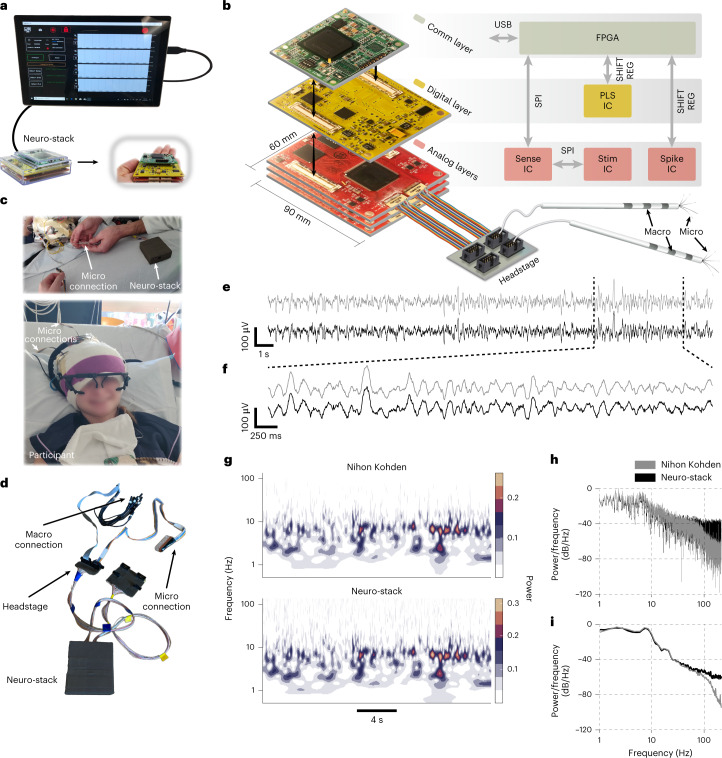
Neuro-stack platform. **a**, Neuro-stack and GUI-based tablet for single-neuron and LFP recordings and closed-loop PLS. The tablet allows for selection of recording and stimulation channel(s), sampling rate, monopolar/bipolar recordings and other parameters. Shown are the packaged (left) and unpackaged (right) versions. **b**, The Neuro-stack consists of three stacked layers: (1) Communication (Comm), (2) Digital and (3) Analog. Presented are the PCBs (size = 90 × 60 mm^2^) and 5 × 2 pins (eight channels, one reference and one ground, ten total pins) Omnetics headstage connectors to which micro-electrodes can be connected (only top Analog layer connected). Note that each Analog layer receives up to two Omnetics connectors to connect, with up to four electrodes through one headstage. A high-level block diagram of each layer is shown (right). The Comm layer contains an FPGA that mediates command and data transmission (via USB) between external software and IC chips. The Digital layer contains the PLS IC. The Analog layer contains chips for sensing (Sense IC) and stimulation (Stim IC). Three Analog layers are shown to allow recording of 192 channels (64 × 3 layers). SPI is used for FPGA communication with the Sense and Stim ICs and shift register for FPGA communication with the PLS and Spike ICs. **c**, The Neuro-stack connected to micro-electrodes in a participant wearing an eye-tracking system. **d**, Shown are ten-pin touchproof jumpers for macro-electrode and ten-pin connectors (for example, Adtech) for micro-electrode recordings. **e**, Example data recorded simultaneously using a clinical monitoring system (Nihon Kohden, gray) and Neuro-stack (black) showing similarity of signals. **f**, Zoomed-in traces from **e**. **g**, Example power spectrograms from data (**e**) showing concordant activity patterns (Nihon Kohden, top; Neuro-stack, bottom). Frequency (0.1–200 Hz) is shown using a logarithmic scale. **h**, Example normalized PSD plots using FFT (0.1–200 Hz, 500-Hz sampling) on data shown in **e**. **i**, Example smoothed normalized PSD plots using the Welch method (1–200 Hz, 500-Hz sampling) on data shown in **e**. Source data

**Fig. 2:**
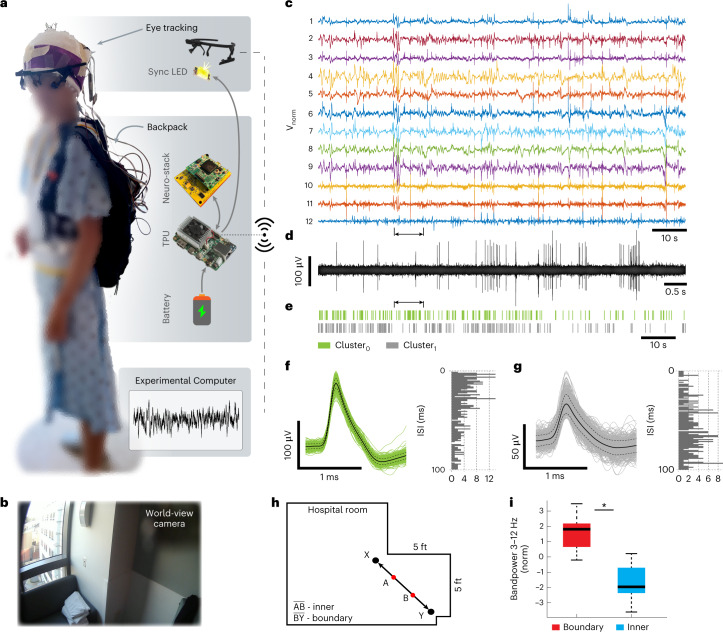
Neuro-stack as a wearable platform for recording neural activity during ambulatory behavior in humans. **a**, An example research participant wearing the backpack carrying the Neuro-stack system, a single board computer with a TPU and a battery, to allow for recording of single-neuron and LFP activity during ambulatory behavior. The participant was also wearing an eye-tracking device that keeps track of head direction, pupil size changes and eye movements. Data captured from the eye-tracker were synchronized with the neural data using a programmable LED that is visible on the eye-tracker world-view camera. Wireless communication among the Neuro-stack, eye-tracker and other external monitoring devices is enabled through a Wi-Fi access point on the TPU device. **b**, Neural activity was recorded during an ambulatory task where participants walked repeatedly (ten times) between two opposite corners of a 5 × 5 ft^2^ room (from X to Y; Extended Data Fig. 2b). Example video frame from the eye-tracking world-view camera as an example participant approached point Y in the room (bottom). **c**, Neural activity (line noise removed, voltage-normalized separately for each channel) from 12 micro-electrode channels (1–6: hippocampus, 6–12: anterior cingulate) during the ambulatory walking task from an example participant. **d**, Ten seconds of filtered data from channel 12 (arrows point to corresponding sections on **c** and **e**). **e**, A raster plot of two single units isolated (Cluster_0_ and Cluster_1_) from channel 12. **f**, Cluster_0_ from channel 12 and its corresponding ISI histogram (right). All detected single-unit waveforms are plotted together with mean (black line) ± s.d. (dotted black line). **g**, Cluster_1_ from channel 12 and its corresponding ISI histogram (right). All detected single-unit waveforms are plotted together with mean (black line) ± s.d. (dotted black line). **h**, Top–down view of the hospital room layout in which an example participant walked back and forth repeatedly between points X and Y within a small area of the hospital room (~25 ft^2^). Points A and B represent points 1/3 and 2/3 of the XY path, respectively, and are used to define the boundary versus inner areas of the room. **i**, Increase in theta (3–12 Hz) bandpower when participants were located near the environmental room boundary (**h**, BY) compared to the inner area of the room (**h**, AB). *P = 5.7 × 10^−5^ (two-sided permutation test, *n* = 16 channels). On each box, the central bolded black line indicates the median, and the bottom and top edges of the box indicate the 25th and 75th percentiles, respectively. The whiskers extend to the most extreme data points, minima on the bottom and maxima on the top. Source data

**Fig. 3:**
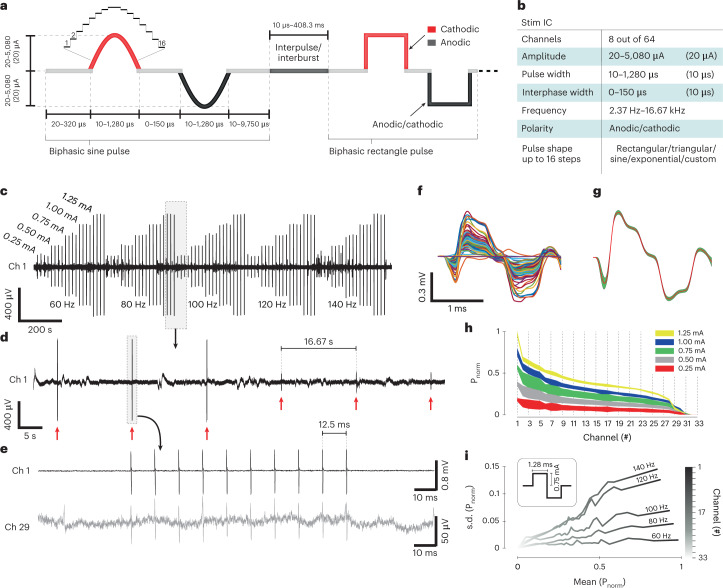
Neuro-stack as a programmable closed-loop neuromodulation system. **a**, Stimulation parameters can be customized, including frequency, amplitude (0–5,080 mA in steps of 20 mA), polarity (anodic/cathodic), timing (of pulse width, inter-phase width and inter-pulse/burst interval) and pulse shape (for example, sinusoidal or rectangular pulses shown). **b**, Key features and capabilities on the stimulation integrated circuit (Stim IC) including the number of channels (that is, eight out of 64 per Analog layer) that can be selected for stimulation, amplitude and configurable pulse shapes where amplitude in each of up to 16 steps (**a**) can be programmed for custom waveform design, frequency, polarity, pulse width (**a**, 10–1,280 μs, steps: 10 μs) and inter-phase width (**a**, 0–150 μs, steps: 10 μs). **c**, Example macro-electrode channel recorded during the delivery of macro-stimulation, which was delivered with varying combinations of amplitudes × frequencies ((0.25, 0.5, 0.75, 1.00 and 1.25) mA × (60, 80, 100, 120 and 140) Hz). Each stimulation burst contained ten biphasic rectangular pulses (pulse width = 1.28 ms), after which a delay of 16.67 s occurred before the next burst cycle. **d**, Zoomed- in view of **c** (outlined box) where six stimulation bursts (red arrows) are shown with different parameters (bursts 1–3: 1.25 mA, 80 Hz; bursts 4–6: 0.25 mA, 100 Hz). **e**, Zoomed-in view of a single burst (outlined box) from the same channel in **d** and another example channel (29). **f**, Time-aligned bipolar pulses from a stimulation burst (ten pulses, 1.25 mA, 60 Hz) from all channels (*n* = 33). **g**, Mean and s.d. values of all time-aligned bipolar stimulation pulses from an example recording channel (1). **h**, Normalized power (mean and s.d.) of the propagated stimulation pulses across channels (*n* = 33) recorded with respect to varying stimulation current (0.25–1.25 mA). **i**, SD of normalized power (SD(power/max[power])) as a function of mean normalized power (mean(power/max[power])) differentiates pulse propagation across channels with respect to varying stimulation burst frequencies (60–140 Hz, steps: 20 Hz) with a fixed pulse width (1.28 ms) and current amplitude (0.75 mA). Electrode channels are marked in shades of gray (*n* = 33). P, power. Source data

**Fig. 4:**
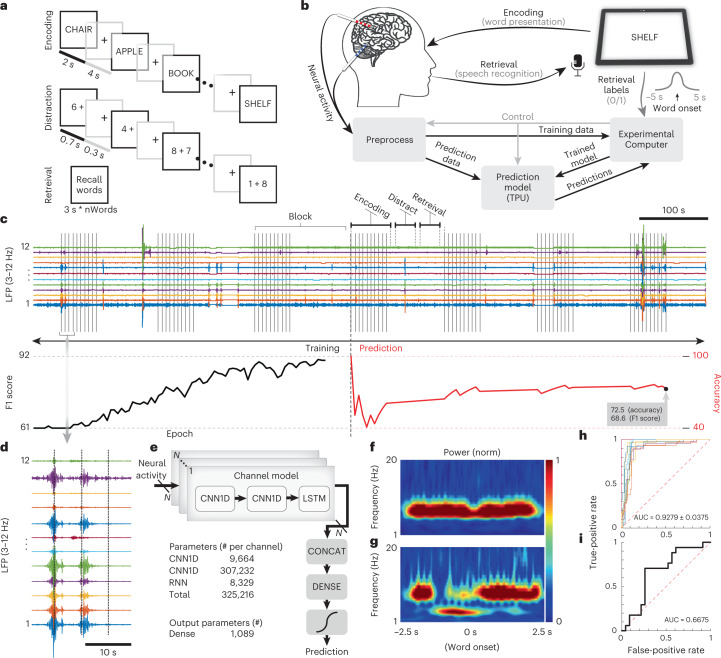
Decoding memory performance with the Neuro-stack system. **a**, Neural activity was recorded during completion of a verbal memory task, which included three phases: (1) Learning (encoding), during which a list of words were presented (2 s each, 0.8-s ISI); (2) Distraction, during which numbers were presented serially (0.7 s each, 0.3-s ISI), and participants were instructed to respond odd/even; and (3) Recall (retrieval), where previously presented words were recalled. **b**, Neuro-stack recording setup and processing pipelines used during the memory task. A tablet was used to present words during encoding and record to identify in real time the spoken words recalled during retrieval (using speech recognition). Minimally processed data were then fed into an external computer with synchronized retrieval results. The neural network model (Model, **e**) was trained in real time to predict retrieval performance based on neural activity during encoding. The model was then ported to the TPU to perform real-time predictions. **c**, Filtered theta (3–12 Hz) activity from the left hippocampus (LHC) is shown because it was the most critical feature used by the trained neural network model to predict memory (top). Vertical lines mark the onset of each word (10) during seven repetitions (blocks) shown of the memory task. Decoding performance (accuracy) is shown (bottom) during the first three blocks, which were used to train the neural network (Training) and the associated F1 score. The last four blocks were used to predict memory performance (Predict) and the associated accuracy. Training and Prediction graphs are not aligned with the task flow in real time (**c**, top) for illustration purposes. **d**, Zoomed-in-view of example theta activity shown in **c**. **e**, The neural network model (2 × CNN1D + LSTM + Dense network) parameters. **f**, Time–frequency representation of the first most significant filter (from the trained CNN layer activation filter), which checks for theta power during encoding. **g**, Time–frequency representation of the second most significant filter (trained CNN layer activation filter), which checks for temporal patterns in theta activity with respect to the onset of word presentation. **h**, Overlapping ROC curves calculated from the offline base model performances across ten participants (colors). **i**, ROC curve from the online prediction phase of the verbal memory study using a single participant’s data recorded on the Neuro-stack. AUC, area under the curve. Source data

**Extended Data Fig. 1:**
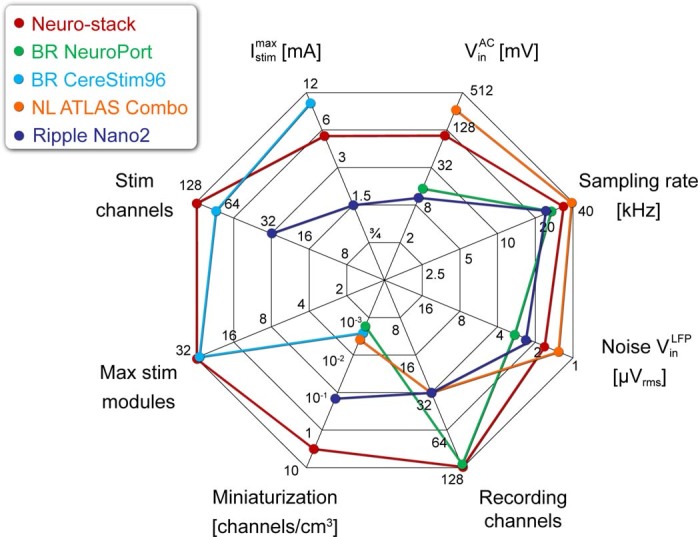
Comparison of Neuro-stack with commonly used bedside intracranial recording and stimulation systems used in humans. Neuro-stack capabilities as compared to existing human intracranial recording and stimulation systems. Characteristics shown include the device sampling rate, noise of the Sense IC (Noise V_in_^LFP^), number of recording channels that can be used, linear input dynamic range (V_in_^AC^), maximum stimulation current (I_stim_), number of channels that can be used for stimulation (Stim channels), and total number of stimulation channels that can be used simultaneously (Max stim modules). The main advantages of the Neuro-stack come from the miniaturization of the electronics per channel (channels/cm^3^) that allow for its small size and wearability and its integrated full-duplex capability that incorporates both stimulation and sensing (red line). BR: Blackrock, NL: Neuralynx.

The Neuro-stack (Fig. 1a,b) provides a bidirectional neuromodulation platform for single-unit, LFP and iEEG recording and stimulation of deep brain areas for basic and clinical neuroscience studies. Compared to larger existing devices (Extended Data Fig. 1) that are used bedside and carried on a cart, the Neuro-stack’s small handheld size enables concurrent stimulation and recording of real-time electrophysiology during freely moving behavior (Fig. 2) by connecting to commonly used implanted macro-electrodes and micro-electrodes (Fig. 1c,d). Apart from its small form factor and unique on-body wearability, the Neuro-stack can support:

1. Recording from up to 256 contacts for a total of 128 monopolar or bipolar recordings with a sampling rate of up to 6,250 Hz. Furthermore, sensing from up to 32 monopolar recordings at 38.6 kHz allows for the recording of single-unit and LFP activity simultaneously.
2. Flexible and programmable stimulation (Fig. 3) allowing for delivery of bipolar/monopolar stimulation to any 32 out of 256 contacts simultaneously. Stimulation engines are current controlled and allow the user to program current amplitude, frequency, timing, pulse shape and other parameters (Fig. 3 and Supplementary Table 3).
3. Closed-loop neuromodulation. The Neuro-stack has built-in (in hardware) theta (3–8 Hz) power detection and the ability to trigger stimulation at a predefined phase of theta activity for PLS. Future hardware upgrades to the PLS integrated circuit (IC) could expand detection to frequencies outside of the theta band depending on user needs. Furthermore, sensing of neural activity is concurrent with stimulation for unrestricted (full-duplex) closed-loop capabilities. Resources for designing custom closed-loop stimulation algorithms are available at both the embedded hardware and external software levels.
4. Software support that comes in two formats. First, a graphical user interface (GUI) running on a Windows-based tablet or laptop is available for manual research purposes (Fig. 1a). Second, a full-access application programming interface (API) library written in C++ allows custom, programmable recording and open/closed-loop stimulation capabilities for research studies (Extended Data Fig. 4).
5. Tensor multiplication accelerator (Edge TPU; Fig. 2a and Extended Data Fig. 4, middle) that is integrated with the Neuro-stack, enabling an extended range of applications, such as real-time inference for neural decoding (Fig. 4) or closed-loop stimulation.
6. Wired or wireless mode. The Neuro-stack can be externally controlled and powered via a USB cable or remotely controlled through a secure local network using a battery-powered configuration (Fig. 2a and Extended Data Fig. 4). This flexibility allows researchers to perform single-unit and LFP/iEEG recording and stimulation during either stationary or ambulatory (freely moving) behavioral tasks.

The central hardware component of the Neuro-stack platform (Fig. 1a,b) consists of three printed circuit board (PCB) layers: (1) Analog, (2) Digital and (3) Communication. Each layer is embedded with one or several dedicated IC chips. The analog layer (Fig. 1b, bottom) contains mixed-signal sensing IC (Sense IC and Spike IC) and stimulation IC (Stim IC) chips, which were previously developed as part of the DARPA SUBNETS program^38–41^. A single Sense IC (one per analog layer) accepts neural activity from up to 64 electrode contacts fed into voltage-controlled oscillators (VCOs), which serve as analog–digital converters (ADCs). Each VCO ADC supports 6,250/*N* Hz sampling frequencies, where *N* = 1, 2, 4, 8, …, 128 and a 100-mV_pp_ linear input dynamic range with 12/21 (macro/micro) bits of resolution, ensuring that the underlying neural signal is captured in the presence of large artifacts (for example, from stimulation). The Sense IC contains digital non-linearity correction to account for non-linear amplification across the input range. Moreover, it also contains a digital logic for adaptive stimulation artifact rejection (ASAR) that subtracts a template stimulation artifact extracted from adjacent channels^41^. The total power consumption per channel is 8.2 µW. A single Spike IC (one per analog layer) accepts neural activity from up to eight micro-wire contacts and supports sampling rates of up to 38.6 kHz, with a 160-mV_pp_ linear input dynamic range^42^. A single Stim IC contains eight engines that can drive current through any individual or combination of 64 electrode contacts. Stimulation output current, amplitude, frequency and waveform shapes are configurable (Fig. 3a,b), which allows for stimulation using previous protocols^43–45^ as well as exploration of novel protocols. These capabilities also enable increased degrees of freedom (timing and PLS; Fig. 3) compared to currently available intracranial neurostimulation systems (Supplementary Table 3).

The Neuro-stack’s digital layer (Fig. 1a,b, middle) routes signals between the analog and communication layers and contains a custom IC chip (PLS IC) for closed-loop stimulation based on the detected oscillatory (for example, theta) phase in the recorded neural signal coming from the analog layer to enable PLS^46,47^. A field-programmable gate array (FPGA, Xilinx Spartan-6 board) serves as a communication layer (Fig. 1a,b, top, and Extended Data Fig. 4) between external devices and custom ICs (Fig. 1b).

The Neuro-stack uses the serial peripheral interface (SPI) at 12 MHz (Sense and Stim ICs) and serial shift register (PLS and Spike ICs) for internal communication between layers and IC chips and a USB interface for external communication and power supply. The device is assembled by physically stacking the described layers (Fig. 1a,b). One Neuro-stack supports up to four analog layers at a time, for up to 256 micro-wire (LFP) electrode contacts (64 per layer) and up to 32 micro-wire (single-unit) electrode contacts (eight per layer).

A ready-to-use GUI is available (connected to the Neuro-stack via USB) and allows for real-time multi-channel monitoring and control of recording and stimulation (Fig. 1a). A platform-agnostic API library written in C++ that allows for custom applications and experiments is also provided. To allow ambulatory experiments, the Neuro-stack can be wirelessly controlled using the Coral Development Board (CDB; Extended Data Fig. 4, tensor processing unit (TPU) in Fig. 2a) and an ARM-based single-board computer, running a Mendel Linux distribution. Similar microprocessors with wireless capabilities, such as a Raspberry Pi, can also be used for this purpose. Our Neuro-stack setup included an ARM-compiled Neuro-stack API, which supports wireless applications through a secure local Wi-Fi (2.4-GHz or 5-GHz) network created using the included API library. Only a device that uses a secure (X.509 certification) connection to a local server can control the Neuro-stack. The CDB contains an onboard TPU (Fig. 2a), which can make real-time inferences for neural decoding or closed-loop applications (for example, see ‘Stationary verbal memory task’ subsection and Fig. 4).

### In vitro sensing and stimulation

The Neuro-stack IC chips (that is, Stim, Sense, Spike and PLS) were validated in vitro^38,39,41,42,46^ and some (Sense and Stim) as part of an implantable system^39^ before moving to human in vivo studies. To validate sensing capability, pre-recorded analog neural data were fed via an NI PXI System (digital-to-analog converter) through a PBS solution. An oscilloscope was used to observe true signals at front-end (FE) inputs and a computer to control and power the Neuro-stack (Extended Data Fig. 5 and Methods). The captured signals were of satisfactory quality (Sense and Spike IC; Extended Data Fig. 6a,b). PLS was also tested using the same in vitro setup (Extended Data Fig. 5). For 300 s of LFP data, the results showed 400 detections within the theta band (3–8 Hz) and triggered stimulations with a circular variance of 0.3 (ref. ^47^).

Measurements of stimulation and synchronization delivery delays were also characterized. First, the round-trip delay, important for closed-loop stimulation, was measured from sensed input to stimulation output by feeding a train of 50 pulses into the sensing FE. The pulse rising edge detection triggered stimulation on the CDB software side (connected to the Neuro-stack via USB; Extended Data Fig. 6c). Input/output observations by the oscilloscope showed a 1.57 ± 0.19-ms round-trip delay (Extended Data Fig. 6d). This result was consistent with the PLS-based round-trip delay of 1.7 ± 0.3 ms measured from the sensed input to stimulation output^47^. Second, synchronization with external devices was done by time-stamping neural samples using the CDB; accuracy depended on the system latency through hardware and software. We applied the same approach as the round-trip delay with the addition of sending a test pulse on a general purpose pin once the sample reached the time-stamping step (Extended Data Fig. 6c), which resulted in a delay, measured from sensed input to CDB output, of 0.56 ± 0.07 ms (Extended Data Fig. 6e). For more details, see Methods (‘In-vitro testing’ subsection).

### In vivo sensing and stimulation

Twelve participants with indwelling macro-electrodes and micro-electrodes implanted for pharmacoresistant epilepsy volunteered for the study. Each Behnke–Fried macro–micro depth electrode (Ad-Tech Medical) contained 7–8 macro-contacts and nine (eight recording and one reference) 40-µm-diameter platinum–iridium micro-wires^48^ inserted through the macro-electrode’s hollow lumen. Neural activity was recorded from macro-wire and micro-wire contacts using the Neuro-stack during wakeful rest (Methods, ‘Participants’ subsection) and from various brain regions (Supplementary Table 2 and Methods, ‘Electrode localization’ subsection). Neuro-stack setup was done bedside (Fig. 1c,d) or on-body during ambulatory movement (Fig. 2a), where the system was connected to implanted electrodes using a custom-built connector (that is, touchproof Cabrio and Tech-Attach connectors for macro-electrodes and micro-electrodes, respectively).

iEEG data were recorded simultaneously with the Neuro-stack using commercially available recording systems (that is, Nihon Kohden) for comparison purposes. Raw iEEG activity traces from an example participant are shown using simultaneous Nihon Kohden and Neuro-stack recordings (Fig. 1e,f), together with time–frequency power spectrum data (frequency band: 1–200 Hz, sampling frequency: 500 Hz; Fig. 1g), fast Fourier transform (FFT) and Welch-based power spectral density (PSD) plots (frequency band: 1–200 Hz, sampling frequency: 500 Hz; Fig. 1h,i). Further comparison metrics^49^ are provided in the Methods (‘Concordance iEEG comparison’ subsection) and Supplementary Table 1.

Stimulation was performed in three participants (P4, P5 and P10; Supplementary Table 2) to test stimulation artifact propagation across channels and assess associated statistics. In the first two participants, bipolar macro-stimulation was applied to the left hippocampus (amplitude: 0.5 mA; pulses/burst: 11; waveform shape: rectangular; pulse width: 1 ms; frequency: 100 Hz). After successful delivery was observed in surrounding channels, a series of bipolar macro-stimulation bursts with varying parameters was delivered in the third participant (P10) (Fig. 3c). Stimulation delivery (Fig. 3c, entire session; Fig. 3d, multi burst; Fig. 3e, single burst level) was observed on 40 nearby recording channels, obtained using the Sense IC (sampling rate: 6,250 Hz). Overlayed pulses from an example burst with the same parameters showed successful delivery across all channels (Fig. 3f). Furthermore, all pulses from the same burst showed consistent artifacts in the channel adjacent to the stimulation site (Fig. 3g). Higher stimulation amplitudes resulted in lower variability in delivered power (Fig. 3h), whereas higher burst frequency resulted in higher variability across channels (Fig. 3i). Note that stimulation artifacts were not caused or affected by channel saturation (Fig. 3f), with absolute voltage levels much lower than the 50-mV cutoff. Thus, these results (Fig. 3h,i) suggest that stimulation parameters, not just underlying neural activity, contribute to stimulation artifact waveform uncertainty. Full quantification of the impact of different stimulation parameters on stimulation artifact statistics should be addressed in future work.

### Ambulatory walking task

**Extended Data Fig. 2:**
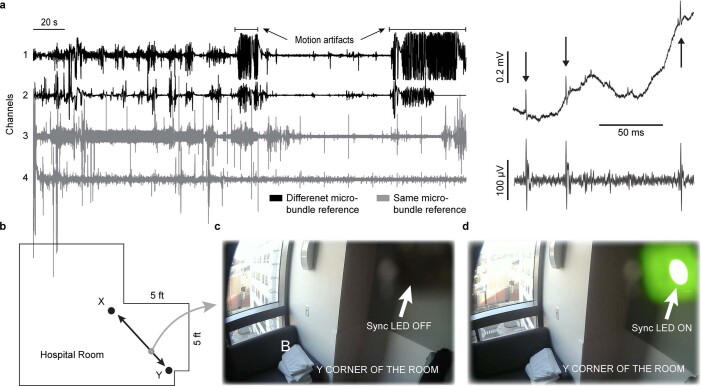
Overview of the ambulatory walking task. **a**, Motion artifacts from four example Neuro-stack recording channels from an example participant during walking behavior when referencing is done using a separate (black, channels 1-2) or the same (grey, channels 3-4) micro-wire bundle. Single-unit activity (example spikes in raw and filtered data shown to the right) that can be extracted using waveform shape and temporal differences as compared to the motion artifact. **b**, Shown is a top-down view of the hospital room layout in which an example participant completed the walking task, during which they were asked to walk back and forth between points X and Y repeatedly. Points X and Y were placed within a small area of the hospital room (~25 ft^2^). **c**, Example screenshots from the world-view camera as an example participant approached point Y from X (gray arrow shown in **b** that was captured via an eye-tracking headset. **d**, Same as **c** but a screenshot when the LED pulse that is used for synchronization (sync LED) was turned on versus off (**c**). Source data

**Extended Data Fig. 3:**
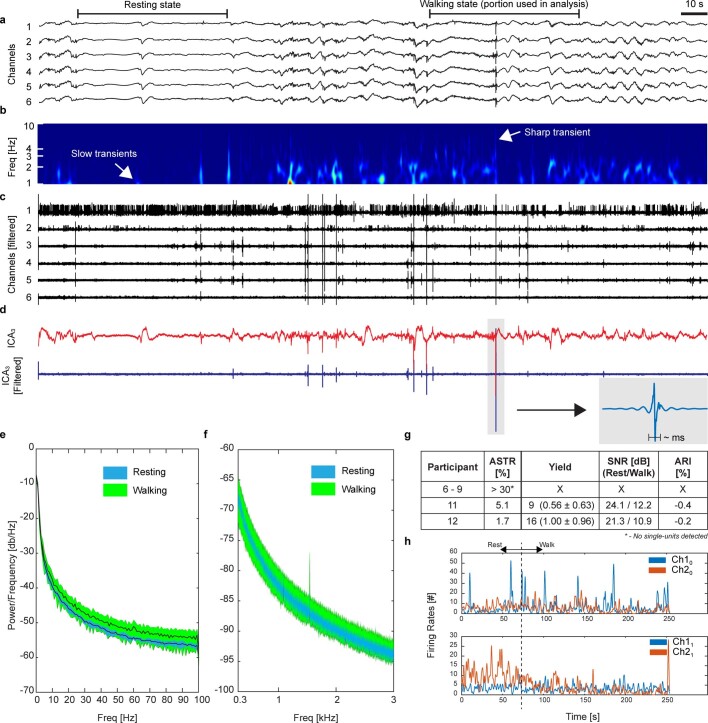
Analysis of recording quality and motion artifacts. **a**, Six example channels (P6-9, P11-12; Supplementary Table 2) of raw recordings from micro-electrodes during wakeful rest and the walking task to illustrate visible slow transient and sharp motion artifacts. **b**, Time-frequency domain (using BOSC) of channel 1 from (**a**), showing impact of slow and sharp transients on the 1–10 Hz frequency (Freq) band. **c**, Same six channels from (**a**), but filtered (300–3000 Hz). **d**, Independent component analysis (ICA) showing the 3^rd^ independent component (ICA_3_) with isolated sharp motion artifacts, which are more problematic than large and slow transients. Red: unfiltered; blue: filtered [300–3000 Hz]). **e**, Power spectral density (power/frequency, mean ± SD; frequency range: 1–100 Hz) over all channels for two conditions (wakeful rest and walking states) matched in time duration. Data from both conditions were preprocessed using a high-pass filter (cut-off 1 Hz) and sharp artifact detection (based on thresholding of the filtered ICA_3_) and removal. **f**, Power spectral density (power/frequency, mean ± SD; frequency range: 300–3000 Hz) over all channels for the same two conditions match in time duration from (**e**). Data from both conditions were preprocessed using a high-pass filter (cut-off 1 Hz) and sharp artifact detection (based on thresholding of the filtered ICA_3_) and removal. **g**, Metrics quantifying the motion artifact removal process and quality of the recordings including artifact-to-signal time ratio (ASTR), yield (total number of sorted units and mean ± SD per channel), signal-to-noise ratio (SNR), and artifact removal impact (ARI) on spike sorting. Single-units were detected in participants P11-12. **h**, Example firing rates over the entire walking task as participant (12) transitioned from the stationary resting state to walking. Shown are two example isolated single-unit clusters from channels 1 and 2. Source data

We used the wireless Neuro-stack setup (Fig. 2a) in six participants (P6–9, P11 and P12; Supplementary Table 2) while they walked in their hospital rooms to record single-neuron activity (Supplementary Table 2) synchronized with world-view and eye-tracking cameras (Fig. 2b and Extended Data Fig. 2c,d). The first four participants walked freely around the room, during which motion artifacts (slow and sharp transients; Extended Data Fig. 3a–c) were examined. To assess quality of recordings, we calculated the artifact-to-signal time ratio (ASTR), yield, signal-to-noise ratio (SNR) and artifact removal impact (ARI) on spike sorting. Slow transients occupied frequency bands lower than 4 Hz and were removed by filtering, whereas sharp transients were detected using independent component analysis (ICA; Extended Data Fig. 3d) and removed by linear interpolation. Although the use of a nearby electrode (same bundle) as a reference reduced common noise artifacts using the FE amplifiers (Extended Data Fig. 2a) and allowed some single units to be isolated (Extended Data Fig. 2a, right) in the first four participants (P6–9), the large ASTRs prevented stabile recordings, successful spike sorting and single-unit cluster isolation. However, after copper shielding of the Neuro-stack’s electronics and headstages, fixation of headstages to the participants’ head and reinforcement of each channel connection, the ASTRs were sufficiently low (Extended Data Fig. 3g) in the next two participants (P11 and P12). Example raw 12-channel neural activity recorded from P11 (Fig. 2c) and P12 (Extended Data Fig. 3a) during walking is shown. Despite a lower SNR during walking compared to rest (Extended Data Fig. 3g), spike sorting^50^ of the bandpass-filtered (300–3,000 Hz) (Fig. 2d) and artifact-free data successfully isolated single-unit clusters (Fig. 2e–g). Recordings were stable and showed non-zero firing rates (for example, Extended Data Fig. 3h) across the task, which included both rest and walking periods (Extended Data Fig. 3a). Furthermore, artifact removal had no significant impact on the results of spike sorting (ARI_11_ = −0.4 ± 1.6%, *P* = 0.287; ARI_12_ = −0.2 ± 1.4%, *P* = 0.354), and average (±s.e.m) single-unit yield across channels (P11 = 0.56 ± 0.63, P12 = 1.00 ± 0.96 units per channel; Extended Data Fig. 3g) was similar to previous studies in stationary participants. LFP activity from the same MTL micro-electrodes recorded in P11 and P12 during the walking task (Fig. 2h) showed that theta activity (3–12 Hz) was significantly increased near the wall (boundary) compared to the inner room area (Fig. 2i; *P* < 0.001) in line with previous findings^35^.

### Stationary verbal memory task

Neuro-stack’s ability to record neural data in real time and decode behavioral performance was tested in a participant (P7) while they completed a verbal memory task (Fig. 4a). During the task, the participant was instructed to learn (encode) a list of ten words and then verbally recall as many as possible after a delay (30 s). During the delay, a non-mnemonic (distraction) task was completed that involved identifying whether the sum of two random numbers (1–9) was odd or even. Encoding, distraction and recall blocks were repeated nine times while the Neuro-stack recorded LFP activity from 16 micro-wire channels, which was used to decode memory performance in real time using artificial neural networks (ANNs). The goal of decoding/classification was to predict the binary outcome of verbal memory (subsequently remembered/forgotten) based on LFP activity during encoding.

**Extended Data Fig. 4:**
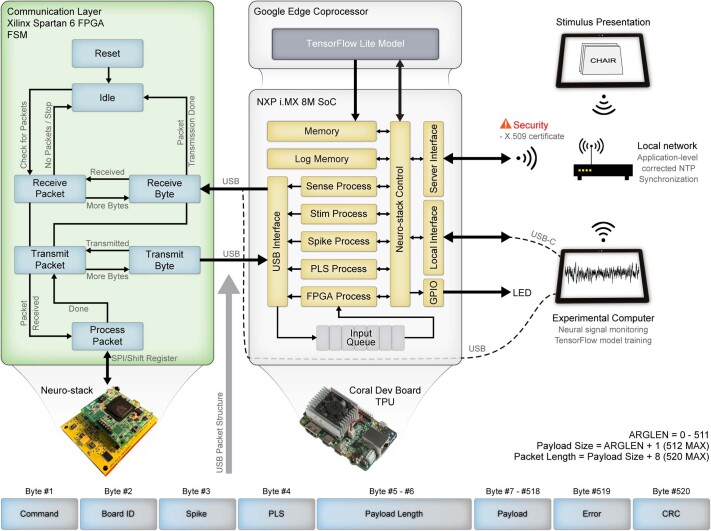
Neuro-stack experimental setup. A simplified block diagram showing Neuro-stack setup including the (LEFT) Communication Layer, which uses a field-programmable gate array (FPGA, Xilinx Spartan 6 model) with a finite-state machine (FSM) built within it to allow for receipt of external messages (packets) via USB (for example, stimulation command) and streaming of the neural data. These packets are then processed and converted to serial peripheral interface (SPI) or Shift Register packets, and transmitted to the Neuro-stack integrated circuits (ICs, SPI: Stim and Sense IC; Shift Register: Spike and PLS IC). This FSM is bi-directional and thus also processes SPI packets received from the Neuro-stack and converts them into USB packets, which are then transmitted to the external Coral Development Board (Coral Dev Board) device. The FSM always begins with a Reset state after a reboot, and then enters an Idle state in which it waits for incoming packets. Once a packet is available, the FSM receives it byte by byte (Receive Byte) until the complete message is transferred (Receive Packet). The received packet is then being processed (Process Packet), converted into the appropriate interface (for example, USB to SPI), and transmitted to the Neuro-stack ICs (via SPI or Shift Register). Similarly, after the processing is done, the response packet from the ICs enters a state during which it can transmit the packet (Transmit Packet) byte by byte (Transmit Byte) externally. Once the transmission is done, the FSM goes back to the Idle state and waits for new packets unless the streaming of the neural data is taking place, in which case the FSM enters Process Packet state indefinitely until the recording is stopped. (CENTER) Coral Dev Board that can directly communicate with the Neuro-stack Communication Layer (via USB connection) using an Application Programming Interface (API, shown here) or a device (for example, Experimental Computer) with an installed GUI via USB (Fig. 1a). The Coral Dev Board contains a regular ARM-based central processing unit (NXP i.MX 8 M SoC) and a Google Edge Coprocessor, a tensor processing unit (TPU). The Neuro-stack API library runs on the ARM processor and contains a real-time pipeline for handling control and data flow to and from the Neuro-stack for each IC and Communication Layer (or FPGA). The Input Queue handles streams of both neural data and acknowledgment receipts from the Communication Layer and redirects them to the appropriate block on the Coral Dev Board responsible for each ICs (for example, Sense, Stim,… Process). The Neuro-stack Control block contains all of the API functions, which are then multiplexed to additional layers responsible for wireless (via Server Interface) or wired (via Local Interface) communication with the Experimental Computer. Additionally, the Neuro-stack Control block also contained functions for controlling the TPU, such as loading/saving the machine learning model (TensorFlow Lite Model) to/from the Memory block, redirecting the data streams directly towards the TPU, and receiving the TPU’s output once it is ready. The incoming neural data streams can also be stored locally in Log Memory or transferred to external storage on the Experimental Computer through the Neuro-stack Control block. Furthermore, an LED light can be triggered (to turn on/off) through available general-purpose input/output (GPIO) pins for synchronization purposes. These triggered on/off events are internally temporally aligned with the incoming neural data in order to synchronize it with data from eye-tracking cameras. (RIGHT) A local network can be created either by using a separate access point (shown here, for example, router, hotspot, etc.), or by the Coral Dev Board, which contains a network controller that can support access point topology and thus can create its own local network. This wireless mode means that a server is created on the Coral Dev Board to allow for other devices, such as the Stimulus Presentation device (for example, iPad used to present the verbal memory task) or Experimental Computer (for example, to view neural data in real-time) to access the Neuro-stack API functions. Security warning points to the importance of a safe wireless connection, implemented by X.509 certificate authentication. Wired mode is also supported through Local Interface block (for example, Experimental Computer connected via USB-C). All devices connected to the local network use Network Time Protocol (NTP) to log events with timestamps fetched from a common server in order to synchronize them. (BOTTOM) The structure of the USB packets sent from the Coral Dev Board to the Neuro-stack Communication Layer, which contains up to 520 bytes that describe the type of Command, Board ID to address specific analog layer, Spike byte, phase-locked stimulation (PLS) byte, and Payload for additional information where its length (Payload Length) depends on the type of command. The packet also contains bytes for error codes (Error) and a cyclic redundancy check (CRC) to detect accidental changes in the raw packets. The communication layer extracts a relevant portion of the USB packet, converts it to desired interface (SPI or Shift Register), and transmits it to the addressed IC. Source data

The TPU device (Extended Data Fig. 4) was integrated with the Neuro-stack and used to embed a neural network model that was small enough to be successful with using solely on-system computation. ANNs were pretrained on multi-channel raw (downsampled) LFP data previously acquired using a Blackrock Neuroport recording system. Offline pretraining performance successfully differentiated remembered from forgotten words during recall with a test F1 score (*F*_1_ = 2 × (*P* × *R*) / (*P* + *R*); *P*, precision, *R*, recall) of 88.6 ± 5.5% and a test accuracy of 91.7 ± 3.3%. The model was built and trained in a Keras (TensorFlow back-end) framework after detailed comparison with commonly used machine learning methods (support vector machine (SVM) and principal component analysis (PCA) plus SVM, various neural network architectures; Supplementary Table 4). The decoder consisted of an input 2 × CNN1D + LSTM (one-dimensional convolutional neural network + long short-term memory) layers that extracted multi-channel LFP features and an output Dense (fully connected network) + Classifier layers (Fig. 4e). For further details, see Methods.

During the memory task, the offline model’s output layers were retrained in real time on an external computer. The trained model was then translated to TensorFlow Lite and ported to the Edge TPU, to predict memory during the last four task blocks (Fig. 4b). The training phase and improving accuracy/loss metrics are presented in Fig. 4c. The online test (prediction) phase resulted in an F1 score of 69% (Fig. 4c, bottom). Average total theta power of the data (Fig. 4d) and a time–frequency heat map of the second CNN1D layer activation filters confirmed that theta multimodal activity timed to the population activations in the left and right hippocampus was used by the model to separate the two classes (Fig. 4f,g). The model’s above-chance performance was confirmed by receiver operating characteristic (ROC) curves (0.5 threshold) for both the offline base model (Fig. 4h) and the online Neuro-stack prediction phase (Fig. 4i). Participant’s memory performance during the testing phase of the online study was 42.5% (summary of recall performances in both training and testing datasets is shown in Extended Data Fig. 7c).

## Discussion

We present the Neuro-stack, a novel miniaturized recording and stimulation system that can interface with implanted electrodes in humans during stationary (bedside) or ambulatory behaviors. The Neuro-stack presents a unique system fully developed and tested in an academic environment (Methods, ‘Neuro-stack development in an academic setting’ subsection), which can deliver closed-loop PLS (to up to 32 channels simultaneously) during single-unit and LFP recording. Full-duplex and PLS capability have been a key challenge in the development of implantable neuromodulation devices due to small margins in devices’ input dynamic range compared to externalized systems. Current human implantable systems require mitigation before recording of neural activity when stimulation is on (for example, amplifier blanking and differential recordings). The Neuro-stack’s larger dynamic (FE) input range and its amplifier’s digital non-linearity correction (NLC) allows for capturing of large-magnitude stimulation artifacts in the absence of amplifier saturation or neural signal degradation. The Neuro-stack also includes a digital ASAR IC^41^, which could provide improved ability to sense multi-channel single-unit/LFP neural activity (with 160-mV_pp_ single-unit and 100-mV_pp_ LFP linear input range) concurrent with stimulation. However, future studies will be needed to characterize advantages and effectiveness of added ASAR capability. Altogether, the Neuro-stack offers an advantage over existing systems in that it provides unrestricted full-duplex with PLS capability during single-unit/LFP recording in the presence of large stimulation artifacts.

A second major advantage of the Neuro-stack is its smaller handheld size that enables it to be carried on-body and wirelessly controlled. These features allowed us to record single-neuron activity during walking, which, to our knowledge, are the first recordings of their kind in humans. Future studies using the Neuro-stack could determine the neural mechanisms underlying human freely moving behaviors to identify, for example, spatially selective neurons and their modulation by cognition (for example, hippocampal place or entorhinal grid cells^51^) that have been discovered in freely moving animals. Doing so would bridge decades of findings between animals and humans and potentially lead the way toward scientifically informed therapies for hippocampal–entorhinal-related dysfunctions (for example, Alzheimer’s disease). Although we did not identify spatially selective single units, possibly due to the restricted spatial environment in which walking took place, future studies using the Neuro-stack over longer distances (for example, hallways) may be able to identify these neurons in humans.

A third advantage of the Neuro-stack is its API that allows fast and flexible prototyping of behavioral experiments with a range of back-end functions that accurately align behavioral and neural events. We demonstrated how an offline neural network model can decode verbal memory performance in a participant with accuracy levels that exceed previous reports^18^, whereas a similar real-time result was obtained using the Neuro-stack’s API integrated with a TPU. Future studies with larger sample sizes will confirm whether our reported decoding accuracy can be improved and generalized across participants. It should be noted that we tested the decoding algorithm in a participant using the model pretrained with recordings from a different device with different noise levels (Fig. 1g); hence, performance could increase as more Neuro-stack data are incorporated into the pretrained model. Given the increasing benefit of using machine learning approaches^52–54^ in neuroscience studies, the Neuro-stack could be useful for validating decoding models and testing novel closed-loop stimulation therapies (for example, to improve memory in patients with severe memory impairments).

Future studies could also determine which stimulation parameters are most beneficial for restoring cognitive or behavioral functions given the Neuro-stack’s highly flexible programmability compared to existing human-approved stimulators. For example, continuous adjustments of custom pulse shapes and/or timing of stimulation relative to ongoing neural activity could allow for the development of more effective therapies. Given the wireless and wearable nature of the Neuro-stack, studies could also determine whether closed-loop stimulation protocols effectively translate to more naturalistic behaviors during everyday experiences that occur during mobility.

Although the Neuro-stack offers several advantages over currently available systems, there are limitations that warrant discussion. First, the Neuro-stack can only support a maximum of 32 single-unit recording channels. Other existing bedside systems can allocate more than 128 channels solely for single-unit recordings. The use of multiple Neuro-stack devices, however, would address this issue and increase single-unit channel count substantially. Second, although the Neuro-stack is small enough to be carried on-body and allows for full mobility, its connection with implanted electrodes is still wired. Thus, significant movements can result in motion artifacts. However, single-unit spike waveforms can still be detected and isolated during walking behavior as we show using techniques such as differential recordings between nearby contacts as well as proper wire isolation and fixation. Lastly, the Neuro-stack can only be used in research studies with patients who have externalized electrodes implanted during clinical (for example, epilepsy) monitoring. Because these patients need to be continuously tethered to bedside intracranial recording systems to assess for symptomatic episodes (for example, seizures), this limits the amount of time a patient can be freely moving. However, ambulatory studies can be completed after clinical data have been captured, as was done in the current study, on the last day of the patient’s hospital stay before electrode de-plantation surgery, or during circumstances where continuous monitoring may not be necessary (for example, depression or chronic pain studies^36,55^). Furthermore, proper precautions and safety measures should be implemented, such as waiting to complete studies until patients with epilepsy are back on anti-epileptic medications to minimize risks associated with seizures during ambulatory tasks.

Although Neuro-stack is much smaller than other external systems, an even smaller version could be tested in future studies, because its IC chips are all implantable^38–42,46^ and require a combined area of 113 mm^2^ (four analog layers). An implantable version of the Neuro-stack^39^ but with its added single-neuron and closed-loop stimulation capabilities thus presents an exciting avenue toward a completely wireless intracranial single-unit and LFP recording system that would not be susceptible to motion artifacts. This would present a considerable advancement over current FDA-approved chronic neurostimulation devices in that it would allow for single-neuron and multi-channel (current state-of-the-art is four channels) recordings, bidirectional recording and PLS stimulation (full-duplex) capability and the ability to use advanced strategies for decoding (for example, neural network models) behavior or disease-related states. Altogether, these capabilities would provide cognitive and clinical neuroscience studies with a promising future pathway toward determining the deep brain mechanisms of naturalistic behavior in humans and developing more effective closed-loop intracranial neuromodulation strategies for individuals with debilitating brain disorders.

## Methods

Equipment designed and used in this research was approved by the University of California, Los Angeles (UCLA) institutional review board (IRB). The research protocol, informed consent and in vivo studies in human participants described in this work were also approved by the UCLA IRB.

### Neuro-stack hardware design

The Neuro-stack was built from four implantable and previously reported application-specific IC chips. The Sense IC contains 32 low-noise, high-dynamic-range LFP sensing FEs, which can be duplexed to 32 electrodes for single-ended recording with respect to the reference electrode or to 32 pairs of electrodes for differential recording, matching up to 64-electrode probes (or 8 × 10-electrode probes, where the 9th is a reference and the 10th is a ground contact). After linearization in the NLC module, the recorded output can be optionally sent to four ASAR engines, which use adjacent recording channels to adaptively learn the shape of the stimulation artifact to improve stimulation artifact removal in real time. The signal processing chain of FE + NLC + ASAR provides the ability to sense neural activity concurrent with stimulation. Each of the steps in this chain can be configured and included/bypassed in the pipeline. The Sense IC provides a three-wire SPI interface. It also downstreams the commands to control the Stim IC. The controller integrated into the Sense IC implements the state machine for SPI communication, schedules the data for the sensing output and features the capability of individual control of every FE/NLC/ASAR module^38–41^. The PLS IC is a previously developed digital chip that supports 16-channel detection of the power at selectable frequencies within theta band (3–8 Hz) and triggers configured stimulation at a specified phase of the detected oscillation^46,47^.

We designed a layout and manufactured a digital (Fig. 1b, middle) and an analog (Fig. 1b, bottom) PCB using specialized software (Altium Designer 14.0) where each board consisted of two PCB layers. The Sense, Stim and Spike IC footprints were placed on the analog layer and the PLS IC footprint on the digital layer. The SPI interface was routed from the analog layer input/output connector to the Sense IC and from the Sense IC to the Stim IC (Fig. 1b, right). We used an SPI with three wires: clock, master input/output slave (MISO) and master output/input slave (MOSI). Two-wire shift register interfaces were routed from the analog/digital layer input/output connector to the PLS IC/Spike IC (Fig. 1b, right). The sensing and stimulation FEs were routed to the two Omnetics PS1-16-AA connectors to which electrodes are connected. The digital and analog layer input/output connectors are compatible and can be stacked on top of each other. On the top connector, we placed the Xilinx Spartan-6 (XC6SLX150-2FGG484C) FPGA board to serve the role of the communication layer (Fig. 1a, top). The FPGA is configured to support four SPI interfaces and five shift registers, thus allowing up to four analog layers to be stacked together. We used a two-analog layer setup for all in vitro and in vivo experiments. Because we used separate SPI interfaces for each analog layer IC, the 4th wire (select) on the SPI was not needed in the PCB design. The FPGA contains a finite state machine (FSM) that converts USB input (FTDI controller) into SPI (SPI controller) packet stream and vice versa. For FPGA programming, we used the Xilinx ISE 14.2 software (Verilog language). In brief, the FSM always begins with a Reset state after a reboot and then enters an Idle state in which it waits for incoming packets. Once a packet is available, the FSM receives it byte by byte (Receive Byte) until the complete message is transferred (Receive Packet). The received packet is then being processed (Process Packet), converted into the appropriate interface (for example, USB to SPI) and transmitted to the Neuro-stack ICs (via SPI or Shift Register). Similarly, after the processing is done, the response packet from the ICs enters a state during which it can transmit the packet (Transmit Packet) byte by byte (Transmit Byte) externally. Once the transmission is done, the FSM goes back to the Idle state and waits for new packets unless the streaming of the neural data is taking place, in which case the FSM enters the Process Packet state indefinitely until the recording is stopped (Extended Data Fig. 4, left). Stacked layers were placed inside a plastic enclosure (Fig. 1a) and wrapped from the inside with copper foil shielding tape to reduce the impact of the noise. Custom headstages (Fig. 1b,d) were built on a protoboard by placing two 5 × 2 connectors on each, which were internally routed to the Omnetics connector.

Neuro-stack’s communication layer uses a USB interface for external connections and a specific communication protocol that can address, configure and start/stop each IC. The protocol is described by a packet structure (up to 520 bytes) that captures Command (such as Reset, Start/Stop, Read/Write configuration registers, etc.), Board ID (to select analog layer), Spike and PLS commands and optional Payload (varies in length (Payload Length) depending on the command). The FPGA’s FSM processes the input packet and decides which IC is to be addressed and forwards relevant bytes to it. The protocol also includes safety error and cyclic redundancy check bytes (Extended Data Fig. 3, bottom). Every command returns its specific acknowledgment receipt, indicating that the execution of the command was successful.

### Neuro-stack software design

The Neuro-stack GUI (Fig. 1a) was built as a Universal Windows Platform application using Visual Studio (2017) and the Visual C# language. The application can be installed on any Windows (8.1 or higher) machine. We specifically used Surface Pro 5 for running the GUI application. The application uses a USB connection to directly communicate with the Neuro-stack (Fig. 1a) to enable viewing and configuration of real-time neural data, the configuration of PLS and other stimulation parameters and manually triggered delivery of stimulation.

As an alternative to the GUI, the Neuro-stack API is a library of functions built in C++ that the user can call in custom-design experiments. The API combines all core and backhand GUI functions into a faster and more resource-efficient implementation. It is built as a multi-thread real-time software pipeline, which threads mirror hardware blocks (for example, Sense Process controls the Sense IC and Stim Process controls the Stim IC; Extended Data Fig. 4, middle). Processes responsible for each IC run in parallel and asynchronously forward commands to their associated IC, or they await a command receipt or a recorded neural sample via the Input Queue (Extended Data Fig. 4, middle). Neural samples are time-stamped using network time protocol (NTP, ref. ^56^) in the Sense and Spike Process threads upon their arrival. They are sent together with a sample value either to an external device or stored in log memory (Extended Data Fig. 4, middle), which was used for synchronization. The library can be compiled for commonly used Linux, Windows, macOS or ARM-based target devices. We used the ARM-based (NXP i.MX 8M SoC) CDB to run the Neuro-stack API. To use all CDB capabilities, we complemented the library with functions that can store/save the TensorFlow Lite model and run inference on recorded neural samples using the CDB’s onboard TPU. CDB supports both wired (USB-C) and wireless (using a local network access point and a TCP/IP server with a X.509 certificate authentication) interfaces with external control capability and use of a real-time monitoring device (for example, Experimental Computer). X.509 is a digital certificate that uses public key infrastructure. We used self-signed certificates because we used only one Experimental Computer to connect to the Neuro-stack. We used a MacBook Pro (2015) laptop as an Experimental Computer, which ran a client Python 3.6.9 script for triggering sensing, stimulation, TPU-specific commands and transferring/storing/monitoring neural activity by using the Neuro-stack API running on the CDB (Extended Data Fig. 4).

For in vivo resting state neural recording experiments, we used the GUI application to control the Neuro-stack (Fig. 1). For in vitro testing, in vivo macro-stimulation (Fig. 3), behavioral stationary (Fig. 4) and ambulatory experiments (Fig. 2), we used the Neuro-stack API and CDB wireless configuration (Extended Data Fig. 3).

### In vitro testing

**Extended Data Fig. 5:**
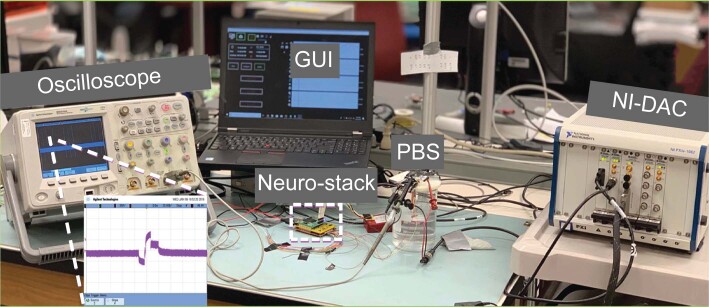
Neuro-stack in-vitro validation setup. Setup for in-vitro validation of recordings and stimulation includes the Neuro-stack platform and GUI, a phosphate-buffered saline (PBS) solution, a NI-DAC (National Instruments Digital to Analog Converter) device for conversion of pre-recorded neural signals, and an oscilloscope for monitoring the signal and measuring delivery time (of synchronization and stimulation pulses). The oscilloscope shows a single pulse of stimulation that was delivered using the Neuro-stack^26^. Source data

**Extended Data Fig. 6:**
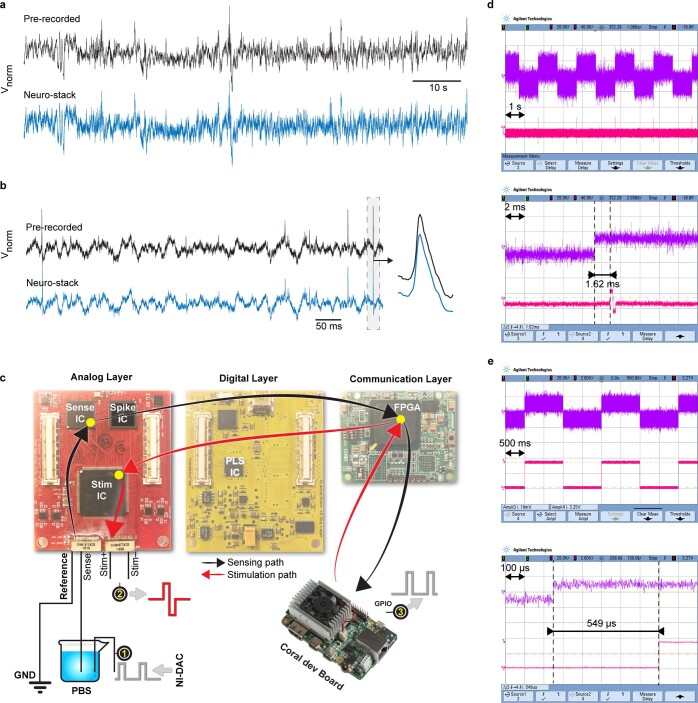
Neuro-stack in-vitro validation results. **a**, Pre-recorded and normalized (V_norm_) LFP signal (black) fed into the Neuro-stack Sense IC front-end and the resulting Sense IC recording (blue). Presented results were filtered to remove 60 Hz noise. **b**, Pre-recorded and normalized (V_norm_) single-unit signal (black) fed into the Neuro-stack Spike IC front-end and resulting Spike IC recording (blue). On the right is a zoomed-in comparison of an example spike-unit waveform. Presented results were filtered to remove 60 Hz noise. **c**, Validation setup from Extended Data Fig. 4 in more detail, showing in-vitro signal sensing path (black arrows; NI-DAC - PBS - Sense IC - FPGA - CDB) and for in-vitro stimulation path (red arrows; CDB - FPGA - Stim IC - PBS). The round-trip delay was measured from point 1 (signal generator) to the CDB (black arrows), and back (red arrows) to point 2 (stimulation output). The test sensing signal was a pulse train fed into the PBS, which triggered stimulation once detected on the CDB. The sensing system delay was measured from point 1 (signal generator) to point 2 (GPIO output). The test signal was again a pulse train, which triggered the GPIO pulse once detected on the CDB. The output stimulation current was converted to voltage using resistors and was observed on the oscilloscope. **d**, The round-trip delay observed on the oscilloscope (Point 1 and 2; zoomed-in at the bottom). **e**, The system sensing delay observed on the oscilloscope (Point 1 and 3; zoomed-in at the bottom). Source data

In vitro studies involved the use of an oscilloscope, a PBS solution, a National Instruments Digital-to-Analog Converter (NI-DAC) and the Neuro-stack (using both wired and wireless configurations; Extended Data Fig. 5). Testing of the Sense and Spike ICs involved feeding 100 s of pre-recorded LFP/single-unit data through the NI-DAC. The analog signals were observed using an oscilloscope and recorded by a single channel using the Neuro-stack. For visualizing results, a time domain comparison was used for Sense IC and Spike IC (Extended Data Fig. 6). The Stim IC was tested as part of closed-loop delay measurements and in previous reports^39^. Delivered stimulation was captured by the oscilloscope and on one channel using the Neuro-stack (Extended Data Figs. 5 and 6). The PLS IC was tested in vitro as part of a previous study^46,47^.

The round-trip delays were measured by sending a pulse train (50 pulses, 20-mV amplitude, 1-s pulse width, duty cycle 50%) from the NI-DAC to one channel recorded using the Neuro-stack. The modified software on the CDB continuously pooled incoming samples and detected the increase from 0 (rising edge) in these incoming values. Once detected, the rising edge triggered one pulse of stimulation. The delay (mean ± s.d. for 50 pulses) was measured on the oscilloscope by capturing both the recording input and stimulation output rising edges and their time difference (Extended Data Fig. 6d).

The Neuro-stack system and software latency from the recording input to the Sense Process thread on the CDB was measured using the same pulse train process, but, instead of triggering stimulation, the detected rising edge triggers a 1-s pulse to the CDB general purpose input/output (GPIO) pin. We used the oscilloscope to observe the recording input and GPIO output and measure the time difference between the rising edges (Extended Data Fig. 6), which was equivalent to the system latency (mean ± s.d. for 50 pulses).

### In vivo testing

#### Participants

Research participants were 12 patients (mean age 24.15 years, nine females; Supplementary Table 2) with pharmacoresistant epilepsy who were previously implanted with acute stereo EEG depth electrodes for seizure monitoring. Participants volunteered for the research study during their hospital stay by providing informed consent according to a research protocol approved by the UCLA IRB.

In each patient, 8–12 flexible polyurethane depth electrodes (1.25-mm diameter) were implanted solely for clinical purposes and before completion of the research study. Each depth electrode terminated in a set of eight insulated 40-μm platinum–iridium micro-wires (impedances 200–500 kΩ).

#### Electrode localization

Electrodes were localized to specific brain regions using methods that have been previously used^57^. In brief, a high-resolution postoperative computed tomography (CT) scan was co-registered to a preoperative whole brain magnetic resonance imaging (MRI) and high-resolution MRI using BrainLab stereotactic localization software (https://www.brainlab.com/) and FSL FLIRT (FMRIB’s Linear Registration Tool^58^). MTL regions, including the hippocampus and entorhinal cortex, were delineated using Automatic Segmentation of Hippocampal Subfields (ASHS^59^) software using boundaries determined from MRI visible landmarks that correlate with underlying cellular histology. White matter and cerebrospinal fluid areas were outlined using FSL FAST software^60^. Macro-electrode and micro-electrode contacts were identified and outlined on the postoperative CT. For a list of localized brain regions in all participants, see Supplementary Table 2.

### Data acquisition and stimulation

For all in vivo validation sessions, a Neuro-stack with two analog layers was used, which allowed for up to two micro-electrode bundles (16 channels) and eight macro-electrodes (32 bipolar or 64 monopolar channels). All micro-electrode and macro-electrode recording sessions were sampled at 38.6 kHz and 6,250 Hz, respectively. Base recordings were done without hardware decimation, non-linear correction and artifact rejection on the Sense IC. Refer to the ‘Data analysis and statistics’ subsection for details about data analyses.

Macro-stimulation was performed in three participants while they rested in their hospital beds. In the first two participants, three stimulation bursts (0.5 mA) were delivered to a single bipolar electrode channel. In a third participant, we performed stimulation propagation mapping, where macro-stimulation was delivered to a single bipolar channel (Fig. 3c,d), and recording was done in the other 40 channels. The parameter test space included (amplitude, frequency) combinations of (0.25, 0.50, 0.75, 1.00 and 1.25) mA × (60, 80, 100, 120 and 140) Hz where every combination was repeated four times for a total of 100 bursts (Fig. 3c) with the following parameters (pulse width: 1.28 ms; interphase width: 150 μs; rectangular pulse shape; interburst delay: 16.67 s). The desired burst frequency was achieved by setting the inter-pulse delay appropriately.

Rectangular pulses recorded in all 40 channels were identified by using cross-correlation across all channels against a template waveform of the delivered stimulation pulse, which was later used for alignment (Fig. 3f,g) and calculating statistics of propagation in 33 out of 40 channels (seven channels did not have artifacts) with respect to varying amplitudes (Fig. 3h) and frequencies (Fig. 3i). For statistical calculations of the propagated power, all pulse waveforms across channels were normalized using the same value of the largest pulse that was propagated.

### Ambulatory walking task

Single-unit data were recorded in six participants during an ambulatory walking task. Four of the participants (P6–9; Supplementary Table 2) were instructed to walk around their hospital room freely and visit prominent ‘landmarks’, such as locations near windows, doors and tables. A separate group of two participants (P11 and P12) was instructed to walk repeatedly (ten times) from one position to another position in the room using a linear path (Fig. 2h). Ambulatory movement and position were tracked using an eye-tracking headset (Pupil Labs Core device^61^), which contained inward-facing eye cameras (sampling rate: 200 frames per second) and an outward-facing world-view camera (sampling rate: 120 frames per second). The recordings were performed using Pupil Capture software (version 2.3). The Neuro-stack was connected to two micro-wire electrode bundles (Behnke–Fried, Ad-Tech Medical) to record from 18 micro-wire contacts (16 recorded single-unit activity, and two served as reference contacts). Recordings with respect to local references (same bundle) were recorded at a sampling rate of 38.6 kHz.

During the walking task, the participants wore an eye-tracker headset and a small backpack (Fig. 2a), which carried the Neuro-stack, the TPU (CDB) using the wireless configuration (Extended Data Fig. 4) and a Voltaic V75 USB Battery Pack. The researcher used an Experimental Computer running an application (Python) to start/stop recordings and view in real time the neural data. Both the Neuro-stack and eye-tracker were connected to the same local network from which the NTP time-stamps were fetched. For a redundant method of synchronization, a miniature LED was attached to the corner of the world-view camera on the eye-tracking headset (Fig. 2a and Extended Data Fig. 2d). The LED was programmed to turn on for 50 ms every 20 s during the experimental walking task, which was not visible to the participant and was also NTP time-stamped.

### LFP analysis

After spike sorting and artifact detection (see ‘Data analysis and statistics’ subsection), we performed LFP extraction and analysis and aligned the data with behavior. Single units and artifacts were removed from the data using linear interpolation of ±1 ms around the detected samples. Spike-free and artifact-free data were downsampled to 386 Hz (using MATLAB’s multistep ‘resample’ function). A high-pass infinite impulse 8th order, 1-Hz cutoff filter (MATLAB’s ‘designfilt’ function) was then applied to the downsampled data.

The BOSC toolbox^62^ was used for time–frequency analysis. The base wavelet included six waves, and the transformation was performed on the 3–90 Hz range (0.25-Hz increments for <30-Hz, 1-Hz increments for ≥30 Hz). The sum of the power time-series over frequency increments resulted in a bandpower range (for example, 3–12 Hz) in time. The bandpower was then normalized, by *z*-scoring each time-series for a frequency band over the entire time-series data, separately for each recording channel.

### Position extraction during the walking task

Each participant’s location in the room during walking was estimated using the Pupil Labs world-view video, Pupil Player software (version 2.3) and pretrained models for optical flow extraction from video frames^63^. First, for each walking trial across the room, the turning frames (points X and Y; Fig. 2h) were identified. Points A and B (Fig. 2h) used for the boundary analysis were determined as the video frames that were, respectively, located at 1/3 and 2/3 of the time that was necessary to cross from X to Y. Bandpower time-series were then separated into two conditions: inner (A to B; Fig. 2h) and boundary (B to Y; Fig. 2h). To correct for a different number of data samples within each of the two conditions, statistical analysis was performed on mean bandpower values from 500 iterations of randomly sampled data from the larger dataset using the length of the lower dataset (MATLAB’s ‘datasample’ function).

### Statistical analyses

A statistical comparison between inner and boundary positions on LFP bandpower from the walking task was completed using a two-sided paired permutation test with 10,000 permutations^35^. We assumed that random sampling of data points between conditions in time, after which the bandpower across channels was calculated satisfied the exchangeability, which is the only condition of a non-parametric permutation test. The *P* value was calculated as the sum of the random differences that were larger than the observed mean difference between the bandpower vectors from all channels, which was then divided by the total number of samples in the distribution. Figure 2i was plotted using MATLAB’s ‘boxplot’ function.

### Stationary verbal memory task

#### Behavioral task

Verbal memory performance was decoded using the Neuro-stack in a single participant (P7; Supplementary Table 2). The memory task began with an encoding period, where the participant was instructed to learn a list of ten words that were randomly selected and serially presented in an audio and visual format on an iPad Pro (3rd generation) screen (Fig. 4b and Extended Data Fig. 4, top right). During encoding, each word was presented for 2 s with an inter-trial fixation period of 4 s. Words were drawn from clusters of six and seven of the word norms and were all 4–8-letter nouns that were rated as highly familiar (range 5.5–7 on a 1–7 scale), moderate to high on concreteness and imagery (range 4.5–6 on a 1–7 scale) and moderate in pleasantness (range 2.5–5.5 on a 1–7 scale)^64^. After the encoding period, participants completed a distractor task where they were instructed to determine whether a presented number (1–9) was odd or even. The distractor task was then immediately followed by a verbal recall period where participants were cued to verbalize as many words as they could remember during a 30-s period. During the experimental paradigm, encoding, distractor and retrieval periods were repeated ten times. Memory performance was calculated as the proportion of previously encoded verbalized words that were recalled.

#### Base neural network model

**Extended Data Fig. 7:**
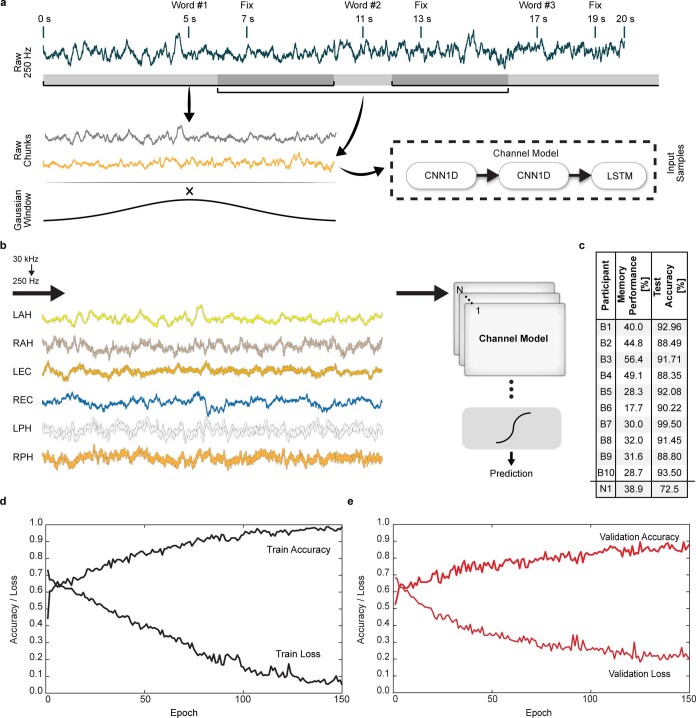
Offline base neural network model. **a**, Neural network model training procedure extracted overlapping 10 s windows of data around the word onsets during the verbal memory task and created raw chunks for each brain region before inputting them into the corresponding channel model. **b**, Downsampled (30 kHz to 250 Hz) windows of LFP data from 6 brain regions: Left/Right Anterior Hippocampus (LAH/RAH), Left/Right Entorhinal Cortex (LEC/REC), and Left/Right Posterior Hippocampus (LPH/RPH), which were range [-1,1] normalized and multiplied with a Gaussian, centered around the word onset. Each channel was then fed into a dedicated Channel Model (N = 6) and concatenated results were then classified. Note that data from all channels from each micro-wire bundle were merged together and then divided into 6 brain region categories. Data was then split into training (50%), validation (25%), and test (25%) data sets for each participants. Training and validation data sets from all participants were merged, shuffled, and used for training and 5-fold validation of the base model. **c**, Shown are 10 participants recorded using a Blackrock system (B1-B10) and the participant recorded using the Neuro-stack (N1 or participant P7 from Supplementary Table 2). Total memory performances for each participant are shown including both training and test samples (2^nd^ column), and accuracy of the base model on the test dataset for each participant (3^rd^ column). Test accuracy for participant N1 is from the online model, which was retrained in real-time during the completion of the verbal memory task. **d**, Train- ing accuracy and loss of the base model. **e**, Validation accuracy and loss, averaged across 5 folds. Source data

For the online binary classification of incoming neural data into remembered/forgotten words, we used a pretrained base neural network model. The base model architecture included two CNN1Ds (1st with 32 nodes, 2nd with 64 nodes) and an LSTM neural network layer with 64 nodes. The L2 regularization was used in the CNN1D and Dense layers and was proportional to the square of the weight coefficients’ value. Moreover, the training dropout technique^65^ was applied after each layer with a 0.2 rate, except for the LSTM, which used a 0.1 rate and a recurrent dropout (0.5 rate). The complete structure of one branch is presented in Fig. 4e. The branches were structurally identical for all brain regions but had different weights after training. The model was pretrained offline using data from six MTL regions (left/right anterior hippocampus, left/right posterior hippocampus and left/right entorhinal cortex) from ten participants who performed the exact same verbal memory task (Fig. 4a) previously using a Blackrock Neuroport system to record neural data. LFP data (sampling rate 250 Hz, batch size 512) were extracted around the verbal memory task word onsets (same Gaussian window as before) and fed into the model for training (Extended Data Fig. 7a). The data from all participants were divided into training (50%), validation (25%) and test (25%) sets. Then, training and validation datasets were combined, shuffled and used for training of the base model (Extended Data Fig. 7c). Because the number of forgotten (words) trials (forgotten class samples) compared to remembered trials (remembered class samples) was always fewer, we randomly selected an equal number of remembered class samples to balance the training dataset. Binary cross-entropy was used for the loss function, with root mean square propagation for the optimizer (learning rate of 0.001). Five-fold cross-validation (Extended Data Fig. 7d, average across folds) was used for validation using the presented hyperparameters. Hyperparameter optimization of the final decoding model (Fig. 4e) was done during the validation phase and with respect to the F1 score (0.5 threshold).

#### Transfer learning and online prediction

**Extended Data Fig. 8:**
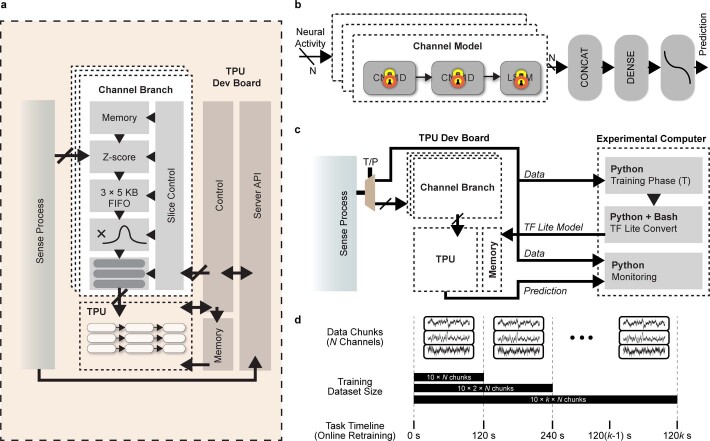
Online embedded Transfer Learning used for retraining and prediction. **a**, Neural network TPU embedded implementation showing the structure of the software responsible for the TPU inference, which consisted of channels from particular brain regions (channel branch). Each channel branch accepted incoming neural data, preprocessed it and then forwarded the output to the Edge TPU once inference was externally triggered. **b**, Online (partial) retraining of the neural network model locked the channel model coefficients. This transfer learning kept coefficients of the base channel models fixed and then retrained all layers after LSTM nodes. **c**, Transfer learning embedded implementation with (partial) external retraining and embedded inference. During the online retraining phase neural samples were directly forwarded over the wireless network to the experimental computer, where Python and Bash scripts automatically performed training and conversion to the TensorFlow Lite model. The model was then automatically transferred to the TPU’s memory, ready to be triggered during the prediction phase. Channel branches were equivalent to those described in (**a**). **d**, Real-time transfer learning retrained the model with shuffled data (old and new). During the online training phase all incoming chunks were used for training as such that whenever new chunks were received, they were shuffled with the previous ones from the same session and used them for retraining.

During the verbal memory task, we used the Neuro-stack in a wireless configuration (Extended Data Fig. 3) together with both the Experimental Computer and Stimulus Presentation device (iPad). We used the Sense IC to record 16 channels from two (left/right hippocampus) micro-wire bundles. Stimulus presentation on the iPad was implemented as a game using Xcode 11.2.1 and Swift 5.0.1 programming languages. For network communication, we used two transmission control protocol (TCP) channels (Fig. 4b and Extended Data Fig. 4; (1) Experimental Computer—CDB and (2) Experimental Computer—iPad). The background processing of the task’s data was divided into two phases: (1) training and (2) prediction, which consisted of five and four blocks of the verbal memory task cycle, respectively (Fig. 4c, presented seven blocks only—three training and four prediction). The purpose of the training phase was to personalize the model for the participant. Only the last two Dense layers from the model were used for retraining and embedding selected filters into the prediction model. The training phase involved downsampling and filtering of raw data (0.1–250 Hz), packing the data separately for each observed brain region (Preprocess step) and transmitting packages from the Neuro-stack externally to the Experimental Computer where the model retraining took place (Fig. 4b and Extended Data Fig. 8a). The words were presented using an iPad Pro tablet, which also used a built-in speech recognition algorithm to supply real-time outcomes (that is, remembered or forgotten) to the Experimental Computer. The word onset events were isolated and weighted using a Gaussian window where one standard deviation was 2.5 s, and cutoffs were made at −5 s and 5 s (before and after word onset), thus giving data around the word onset higher priority. The retraining of the model took place during every Distraction phase (30 s) of the verbal memory task. Once retrained, the model was automatically converted (Python 3.6.9 and Bash scripts) on the Experimental Computer from TensorFlow 2.2 to TensorFlow Lite and uploaded wirelessly to the Edge TPU (Extended Data Fig. 8c). During the prediction phase, the same format of preprocessed data was rerouted to the Edge TPU, where prediction took place. The predictions from TPU and labels from the iPad were transmitted to the Experimental Computer for performance assessment after each word trial (Fig. 4b).

Transfer learning was used to decode performance on the verbal memory task in a single participant. During the training phase with Neuro-stack, we used the same training parameters except that CNN1D and LSTM layer coefficients were fixed (Extended Data Fig. 8b), and only Dense coefficients were adjusted. Also, we used only two model branches out of six that were previously trained on the Blackrock-acquired data (hippocampal channels only) to match the left/right hippocampal electrode placement in the single participant who performed the verbal memory task Neuro-stack experiment. During the online training phase, all incoming windows (chunks) of LFP data were continuously combined with previous chunks, shuffled and used for retraining, whereas the new retraining iterations done after each learning block updated the coefficients saved from the previous learning blocks (Extended Data Fig. 8d). This way, the retraining process used a semi-shuffled dataset as the training set and then sequentially updated it with incoming data. Participants (Blackrock: B1–B10; Neuro-stack: N1 (P7)), their memory performance during verbal memory task and test accuracies using offline (B1–B10) and online (N1) models are shown in Extended Data Fig. 7c.

#### Visualization

To get insight into the trained convolutional portion of the network model during inference, we observed its filter activations by visualizing the patterns that the filters were meant to respond to. Specifically, we applied gradient ascent at the input chunk values so as to maximize the response of a specific filter. The starting input chunk was 10 s with all samples having a value of 0. The resulting chunk was the one that the chosen filter was maximally responsive to. This was performed at the output of every filter in the second CNN1D layer for all channel models. The process aimed to build a loss function that maximized the output of each filter and then to apply stochastic gradient descent, which adjusted the input chunk values so that the filter output values were maximized. The loss function used was an average of the output for a given filter, and the gradient was with respect to the channel model input chunk. We also used L2 normalization during gradient descent. Once completed, the resulting input chunk was transformed into the time–frequency domain using continuous wavelet transform with complex Morlet base, to visualize whether the CNN1D layers were using specific oscillatory bands known to be signatures of verbal memory encoding. Illustrated are filters from the middle hippocampal branch, which maximally responded to theta bands (4–8 Hz) around the word onset (Fig. 4f,g). Association between hippocampal and MTL theta activity and memory function has been well established^18,66^. Note that these results (Fig. 4f,g) do not necessarily suggest that neural 10 s of data with strong theta power around the word onset is predictive of successful encoding. Rather, these results suggest that filters with a time–frequency transfer function that isolates theta activity (Fig. 4f,g) contributed to the model’s final decision, ultimately made by layers that followed the second CNN1D layer, which could have been either a remembered or a forgotten word.

#### Other classification methods

The above-described neural network model was chosen after an extensive trial-and-error process during which multiple classification algorithms were tested on the same dataset. Specifically, before using the neural network model, the data were classified using shallow methods, such as SVM. As part of the feature engineering process, we supplied SVM models with raw, power and phase data in 0–250-Hz range chunks of 7 s (word onset at 3.5 s) or in a sequence of 1-s sliding time windows (with no overlap). Before choosing the final decoding model, we also tested several convolutional neural network (CNN) and recurrent neural network (RNN) architectures. Summary of the accuracies for each of these decoding methods is presented in Supplementary Table 4.

### Neuro-stack development in an academic setting

The Neuro-stack is a neural interface that was designed, validated and tested in human participants all at an academic center, unlike other existing devices discussed (for example, NeuroPace, Medtronic and Blackrock Microsystems), which were developed in commercial environments. The Neuro-stack development was informed by in vivo human testing (for example, wearability, pre-existing stimulation protocols and available online electrophysiological data processing), all of which were made possible by research and close collaboration among academic researchers across the Departments of Electrical and Computer Engineering, Neurosurgery and Neurology and the Neuroscience Interdepartmental Program at UCLA.

As mentioned previously, much of the hardware (Sense, Spike and Stim ICs) integrated in the Neuro-stack was developed for the implantable SUBNETS system through a multi-institutional effort that was initiated, supported and funded by DARPA. The SUBNETS system and its components were developed following standard operating procedures and FDA guidance for active implantable medical devices incorporating requisite International Organization for Standardization (ISO) standards. In addition to the Sense IC for LFPs and Stim IC for the SUBNETS program, an additional Sense IC for spikes and a PLS IC were developed, conforming to the same guidelines. The technology incorporated in the Neuro-stack was developed in two fabrication cycles (1st iteration^40^ and 2nd iteration^39^), with a series of tests involving benchtop verification with in vitro validation. In the final version of the system, Sense, Spike and PLS ICs were designed and fabricated at Taiwan Semiconductor Manufacturing Company using a 40-nm complementary metal-oxide semiconductor (CMOS), whereas the Stim IC was fabricated at X-FAB using a high-voltage 180-nm CMOS. Neuro-stack assembly and software development were internally verified and validated at UCLA to meet safety requirements set by the FDA and the UCLA IRB. The functionality of each hardware and software component was, thus, thoroughly tested and documented before obtaining IRB approval. This included testing of recording functionalities at specified parameters for a given channel, at a defined sampling frequency and under a specific amplifier configuration and not others. Likewise, validation of stimulation capability was done to ensure that software control and triggering of stimulation delivered current with the exact programmed parameters, as per ISO 14708-1 and ISO 14708-3. Given that stimulation requires additional safety checks, a separate condition, which enables stimulation, needed to be checked at the firmware level to ensure that delivery could happen only during triggered stimulation trials and not others. This ensured a redundant check in cases where an altered command would be read by the firmware as a stimulation command. Furthermore, all commands sent to the firmware contained an 8-bit cyclic redundancy check (CRC) code to reduce the probability of an incorrect command delivery. During validation and human in vivo testing, only one password-protected and encrypted experimental computer containing a signed certificate was used to control the Neuro-stack, thus simplifying the necessary security infrastructure required for a commercial medical device.

Leakage currents of all channels were verified independently of software and hardware designers by a clinical engineer in an idle, active recording and stimulation mode of operation. Furthermore, all hardware and software documentation, including but not limited to design history, schematics, code and in vitro validation tests, were reviewed and approved by an independent clinical engineer at the UCLA Ronald Reagan Medical Center as precursor to the IRB review process.

### Reproducibility

The Neuro-stack was completed as part of a multi-institutional effort involving a large group of academic researchers over several years. The Neuro-stack integrates hardware (IC) components already developed as part of a previous program (DARPA SUBNETS) with additional development of firmware and software to enable practical research applications with human participants who have intracranial electrodes already implanted. Thus, reproduction of the Neuro-stack would first require replication of individual application-specific integrated circuits (ASICs) reported in previous work (^38–42,46^). It would take additional effort and resources for UCLA to manufacture and distribute the devices and provide user manuals and customer support. Additionally, revisions of the technology aimed at manufacturing economy of scale (for example, integration in 180-nm technology) and miniaturization for dual external and future implant use would be desired to increase access and utility of the technology in clinical research.

### Data analysis and statistics

All custom data analyses were performed using MATLAB (2021b; Wavelet, Signal Processing, Statistics and Machine Learning Toolboxes) and Python 3.6.9.

#### iEEG power spectrum extraction

Unless specified otherwise, all time–frequency power scalograms were obtained using continuous wavelet transform (CWT) (MATLAB ‘cwt’ command) performed on z-scored time-domain data (each channel normalized separately). The base wavelet chosen was the complex Morlet with a symmetry parameter (gamma) equal to 3 and a time–bandwidth product equal to 60. The wavelet coefficients were calculated at 70 logarithmic frequency points from 1 Hz to 125 Hz, after which the squared absolute value of the coefficients resulted in a power scalogram.

All frequency power spectrums were obtained using FFT (MATLAB ‘fft’ command). The FFT length chosen was the largest power of 2, less than the length of the observed iEEG trace. The coefficients were then normalized with the trace length. Finally, the squared absolute value of the spectral coefficients multiplied by 2 (one-sided FFT) resulted in the power spectrum. The smoothed PSD plot (Fig. 1i) was calculated using MATLAB’s ‘pwelch’ command.

#### Motion artifact detection and removal

Artifacts during the walking task due to movement of the participant and/or Neuro-stack cables were present in two forms: large slow and large sharp transients (Fig. 2a and Extended Data Fig. 3a,b). Although almost all slow transients occupied a frequency range below 1 Hz, sharp transients affected both the LFP (Extended Data Fig. 3b) and single-unit (Extended Data Fig. 3c) frequency bands of interest. To isolate sharp transients, we used ICA. Three components were chosen after an exploratory phase with a criterion of computing a component that did not include single units after spike sorting (component ICA_3_; Extended Data Fig. 3d). Filtered (300–3,000 Hz) ICA_3_ clearly showed much larger (>1 mV) transients than the usual single-unit waveforms, although they could have the same time resolution (Extended Data Fig. 3d). Sharp transients were detected when the z-scored filtered ICA_3_ envelope was higher than four standard deviations. Each block of consecutive artifactual samples, including 1 ms before and 1 ms after the block, was removed using linear interpolation (MATLAB’s ‘interp1’ function). The proportion of data removed for each participant is shown in Extended Data Fig. 3g.

#### Spike sorting

We performed spike sorting using Wave_clus 3 (ref. ^50^). Preprocessing included the use of a notch filter to remove 60-Hz noise. Selected clusters were chosen so that more than 250 spikes were identified and that, out of these, 1% or less had ISIs of less than 3 ms. Firing rate was calculated by counting the number of spikes in non-overlapping 50-ms windows across the duration of the experiment and convolving the resulting time histogram with 50-point Gaussian window.

#### Quality of recordings

To quantify the quality of single-unit recordings, we observed firing rate stability and calculated yield, SNR, ASTR (percentage of artifactual samples in the observed multi-channel time-series) and ARI on spike sorting. Yield was defined as the number of successfully extracted units after spike sorting per electrode. Stability was visually observed through range-normalized firing rates of each clustered unit (for example, Extended Data Fig. 3h). SNR was defined as the maximum unit amplitude of the average waveform for each sorted unit divided by three average standard deviations of the background noise (obtained from 300–3,000-Hz range; Extended Data Fig. 3f). SNR was calculated during two separate conditions: wakeful rest and walking (Extended Data Fig. 3a). ASTR was defined as the number of data samples removed after the artifact removal process (see ‘Motion artifact detection and removal’ subsection) divided by the total number of samples for each channel. For ASTR, we calculated the average value (Extended Data Fig. 3g) across all channels with the standard deviation being less than 1% of the mean as identical motion artifacts were present in all channels. ARI was defined as a mean (across units) percent change in the number of spikes before and after motion artifact removal (Extended Data Fig. 3g).

#### Concordance iEEG comparison

Figure 1e–i provides visual comparisons of iEEG recordings acquired from the Neuro-stack and Nihon Kohden systems in time, frequency and time–frequency domains. To provide a more systematic comparison of the presented data (Fig. 1e), we also used additional metrics, such as Pearson correlation, Hjorth parameters (activity, mobility and complexity), artifact spike count, 60-Hz power and kurtosis (Supplementary Table 1). These metrics were obtained from ref. ^49^ and defined as follows:

$$\begin{array}{c} {{\mathrm{Pearson}}\,{\mathrm{correlation}}\left( {\rho \left( {A,B} \right)} \right)} = {\frac{1}{{N - 1}}\mathop {\sum}\limits_{i = 1}^N {\left( {\frac{{A_i - \mu _A}}{{\sigma _A}}} \right)\left( {\frac{{B_i - \mu _B}}{{\sigma _B}}} \right)} } \\\qquad\ \,{{\mathrm{Activity}}} = {{\mathrm{var}}(\,y(t))} \\\qquad\ \ \, {{\mathrm{Mobility}}} = {\sqrt {\frac{{{\mathrm{var}}\left( {\frac{{dy(t)}}{{dt}}} \right)}}{{{\mathrm{var}}(\,y(t))}}} } \\\qquad {{\mathrm{Complexity}}} = {\frac{{{\mathrm{Mobility}}\left( {\frac{{dy(t)}}{{dt}}} \right)}}{{{\mathrm{Mobility}}(\,y(t))}}} \\ {{\mathrm{Kurtosis}}} = {\frac{{E(\,y - \mu )^4}}{{\sigma ^4}}} \end{array}$$

The artifact spike count represented the number of sample points within an observed z-scored iEEG trace (250-Hz sampling rate) that fell outside ±6 value range. Power of 60 Hz was calculated using MATLAB’s ‘bandpower*’* function for the frequency range 55–65 Hz at a sampling frequency of 250 Hz.

### Statistics and reproducibility

Research participants were 12 patients (mean age 24.15 years, nine females) who took part in four types of experiments: (1) stationary recording, (2) ambulatory recording, (3) stationary stimulation and (4) stationary verbal. Each experiment and data analyses were previously described in corresponding sections. Participants were not offered any compensation for their involvement in this research. No statistical methods were used to predetermine sample size. Because the main aim of this study was the validation of recording and stimulation capabilities of our developed Neuro-stack system, we chose sample sizes that are similar to (or larger than) previous studies, where similar recordings were performed with other similar technical systems^34,67^.

Participants were asked to perform different experimental tasks (that is, stationary recording, recording during ambulatory walking, stationary stimulation or verbal memory) based on their physical, cognitive and clinical condition. Thus, selection of participants or assignment to different experimental tasks, sample sizes and replication decisions were determined in close collaboration with the clinical staff and were primarily based on the participant’s condition. For example, stationary stimulation required the presence of neurologists on-site for safety reasons (as per IRB and safety requirements), and only participants in good physical condition who were able to walk safely were asked to participate in the ambulatory walking task.

For these reasons, full randomization and random assignment of participants to experimental tasks was not possible in this study (Supplementary Table 2). Given that the main aim of this work was the validation of the Neuro-stack recording and stimulation system, rather than empirical conclusions regarding cognitive or behavioral effects in individuals, it is the authors’ opinion that this non-random assignment of participants to experimental tasks had minimal or no impact on the relevance of the work.

Data collection and analysis were not performed blinded to the conditions of the experiments. Data from each participant (Supplementary Table 2) were analyzed separately, but not all results or redundant conclusions about recording or stimulation signal quality were presented for each participant. Two participants (not included in this work), who attempted to perform the stationary verbal memory task, were excluded from analysis because the session was stopped at their request before sufficient results could be obtained.

### Reporting summary

Further information on research design is available in the Nature Portfolio Reporting Summary linked to this article.

## Online content

Any methods, additional references, Nature Portfolio reporting summaries, source data, extended data, supplementary information, acknowledgements, peer review information; details of author contributions and competing interests; and statements of data and code availability are available at 10.1038/s41593-023-01260-4.

## Supplementary information

Supplementary Information Supplementary Tables 1–4.

Reporting Summary

Supplementary Data 1 Editorial Assessment Report.

## Data Availability

Data are available upon reasonable request. Source data are provided with this paper.

## Source data

Source Data Fig. 1 Contains data (Figure1_source_data.xlsx) and images (Figure1_source_images) used in making Fig. 1.
Source Data Fig. 2 Contains data (Figure2_source_data.xlsx) and images (Figure2_source_images) used in making Fig. 2.
Source Data Fig. 3 Contains data (Figure3_source_data.xlsx) and images (Figure3_source_images) used in making Fig. 3.
Source Data Fig. 4 Contains data (Figure4_source_data.xlsx) and images (Figure4_source_images) used in making Fig. 4.
Source Data Extended Data Fig. 2 Contains data (EDFigure2_source_data.xlsx) and images (EDFigure2_source_images) used in making Extended Data Fig. 2.
Source Data Extended Data Fig. 3 Contains data (EDFigure3_source_data.xlsx) and images (EDFigure3_source_images) used in making Extended Data Fig. 3.
Source Data Extended Data Fig. 4 Contains data (EDFigure4_source_data.xlsx) and images (EDFigure4_source_images) used in making Extended Data Fig. 4.
Source Data Extended Data Fig. 5 Contains data (EDFigure6_source_data.xlsx) and images (EDFigure6_source_images) used in making Extended Data Fig. 6.
Source Data Extended Data Fig. 6 Contains data (EDFigure6_source_data.xlsx) and images (EDFigure6_source_images) used in making Extended Data Fig. 6.
Source Data Extended Data Fig. 7 Contains data (EDFigure7_source_data.xlsx) and images (EDFigure7_source_images) used in making Extended Data Fig. 7.

## Code availability

Code is available upon reasonable request.

## References

[1] Gardner RJ, Hermansen E, Pachitariu M, Moser EI (2022). Toroidal topology of population activity in grid cells. Nature.

[2] Musall S, Kaufman MT, Juavinett AL, Gluf S, Churchland AK (2019). Single-trial neural dynamics are dominated by richly varied movements. Nat. Neurosci..

[3] Kubska ZR, Kamiński J (2021). How human single-neuron recordings can help us understand cognition: insights from memory studies. Brain Sci..

[4] Engel AK, Moll CKE, Fried I, Ojemann GA (2005). Invasive recordings from the human brain: clinical insights and beyond. Nat. Rev. Neurosci..

[5] Quian Quiroga, R. Plugging in to human memory: advantages, challenges, and insights from human single-neuron recordings. *Cell* **179**, 1015–1032 (2019). 10.1016/j.cell.2019.10.016 31730847

[6] Rutishauser U, Reddy L, Mormann F, Sarnthein J (2021). The architecture of human memory: insights from human single-neuron recordings. J. Neurosci..

[7] Schulze-Bonhage A (2017). Brain stimulation as a neuromodulatory epilepsy therapy. Seizure.

[8] Benabid AL, Pollak P, Louveau A, Henry S, de Rougemont J (1987). Combined (thalamotomy and stimulation) stereotactic surgery of the VIM thalamic nucleus for bilateral Parkinson disease. Appl. Neurophysiol..

[9] Vidailhet M (2005). Bilateral deep-brain stimulation of the globus pallidus in primary generalized dystonia. N. Engl. J. Med..

[10] Ressler KJ, Mayberg HS (2007). Targeting abnormal neural circuits in mood and anxiety disorders: from the laboratory to the clinic. Nat. Neurosci..

[11] Nuttin B, Cosyns P, Demeulemeester H, Gybels J, Meyerson B (1999). Electrical stimulation in anterior limbs of internal capsules in patients with obsessive-compulsive disorder. Lancet.

[12] Lozano AM (2016). A phase II study of fornix deep brain stimulation in mild Alzheimer’s disease. J. Alzheimers Dis..

[13] O’Keefe J, Recce ML (1993). Phase relationship between hippocampal place units and the EEG theta rhythm. Hippocampus.

[14] Huxter J, Burgess N, O’Keefe J (2003). Independent rate and temporal coding in hippocampal pyramidal cells. Nature.

[15] Buzsáki G (2010). Neural syntax: cell assemblies, synapsembles, and readers. Neuron.

[16] Jacobs J, Kahana MJ, Ekstrom AD, Fried I (2007). Brain oscillations control timing of single-neuron activity in humans. J. Neurosci..

[17] Rutishauser U, Ross IB, Mamelak AN, Schuman EM (2010). Human memory strength is predicted by theta-frequency phase-locking of single neurons. Nature.

[18] Ezzyat Y (2017). Direct brain stimulation modulates encoding states and memory performance in humans. Curr. Biol..

[19] Suthana, N. et al. Memory enhancement and deep-brain stimulation of the entorhinal area. *N. Engl. J. Med.* **366**, 502–510 (2012). 10.1056/NEJMoa1107212 PMC3447081 22316444

[20] Inman CS (2018). Direct electrical stimulation of the amygdala enhances declarative memory in humans. Proc. Natl Acad. Sci. USA.

[21] Titiz AS (2017). Theta-burst microstimulation in the human entorhinal area improves memory specificity. eLife.

[22] Mankin EA, Fried I (2020). Modulation of human memory by deep brain stimulation of the entorhinal–hippocampal circuitry. Neuron.

[23] Mankin EA (2021). Stimulation of the right entorhinal white matter enhances visual memory encoding in humans. Brain Stimul..

[24] Kucewicz MT (2018). Evidence for verbal memory enhancement with electrical brain stimulation in the lateral temporal cortex. Brain.

[25] Ezzyat Y (2018). Closed-loop stimulation of temporal cortex rescues functional networks and improves memory. Nat. Commun..

[26] Zelmann R (2020). CLoSES: a platform for closed-loop intracranial stimulation in humans. Neuroimage.

[27] Kuo C-H, White-Dzuro GA, Ko AL (2018). Approaches to closed-loop deep brain stimulation for movement disorders. Neurosurg. Focus.

[28] Little S (2013). Adaptive deep brain stimulation in advanced Parkinson disease. Ann. Neurol..

[29] Swann NC (2018). Adaptive deep brain stimulation for Parkinson’s disease using motor cortex sensing. J. Neural Eng..

[30] Sun FT, Morrell MJ (2014). The RNS System: responsive cortical stimulation for the treatment of refractory partial epilepsy. Expert Rev. Med. Devices.

[31] Cummins DD (2021). Chronic sensing of subthalamic local field potentials: comparison of first and second generation implantable bidirectional systems within a single subject. Front. Neurosci..

[32] Stanslaski S (2018). A chronically implantable neural coprocessor for investigating the treatment of neurological disorders. IEEE Trans. Biomed. Circuits Syst..

[33] Kremen V (2018). Integrating brain implants with local and distributed computing devices: a next generation epilepsy management system. IEEE J. Transl. Eng. Health Med..

[34] Gilron R (2021). Long-term wireless streaming of neural recordings for circuit discovery and adaptive stimulation in individuals with Parkinson’s disease. Nat. Biotechnol..

[35] Stangl M (2021). Boundary-anchored neural mechanisms of location-encoding for self and others. Nature.

[36] Scangos KW, Makhoul GS, Sugrue LP, Chang EF, Krystal AD (2021). State-dependent responses to intracranial brain stimulation in a patient with depression. Nat. Med..

[37] Aghajan M (2017). Theta oscillations in the human medial temporal lobe during real-world ambulatory movement. Curr. Biol..

[38] Jiang, W., Hokhikyan, V., Chandrakumar, H., Karkare, V. & Markovic, D. 28.6 A ±50mV linear-input-range VCO-based neural-recording front-end with digital nonlinearity correction. *IEEE J. Solid-State Circuits* **52**, 173–184 (2017).

[39] Rozgic D (2018). A 0.338 cm^3^, artifact-free, 64-contact neuromodulation platform for simultaneous stimulation and sensing. IEEE Trans. Biomed. Circuits Syst..

[40] Rozgic, D. et al. A true full-duplex 32-channel 0.135cm^3^ neural interface. In *2017 IEEE Biomedical Circuits and Systems Conference (BioCAS)* (IEEE, 2017).

[41] Basir-Kazeruni, S., Vlaski, S., Salami, H., Sayed, A. H. & Markovic, D. A blind Adaptive Stimulation Artifact Rejection (ASAR) engine for closed-loop implantable neuromodulation systems. In *International IEEE/EMBS Conference on Neural Engineering (NER)* (IEEE, 2017).

[42] Chandrakumar, H. & Markovic, D. An 80-mVpp linear-input range, 1.6-GΩ input impedance, low-power chopper amplifier for closed-loop neural recording that is tolerant to 650-mVpp common-mode interference. *IEEE J. Solid-State Circuits* **52**, 2811–2828 (2017).

[43] Zangiabadi, N. et al. Deep brain stimulation and drug-resistant epilepsy: a review of the literature. *Front. Neurol.* **10**, 601 (2019). 10.3389/fneur.2019.00601 PMC6563690 31244761

[44] Koeglsperger, T., Palleis, C., Hell, F., Mehrkens, J. H., & Bötzel, K. Deep brain stimulation programming for movement disorders: current concepts and evidence-based strategies. *Front. Neurol.* **10**, 410 (2019). 10.3389/fneur.2019.00410 PMC6558426 31231293

[45] Ramasubbu R, Lang S, Kiss ZHT (2018). Dosing of electrical parameters in deep brain stimulation (DBS) for intractable depression: a review of clinical studies. Front. Psychiatry.

[46] Alzuhair, A. & Marković, D. A 216 nW/channel DSP engine for triggering theta phase-locked brain stimulation. In *2017 IEEE Biomedical Circuits and Systems Conference (BioCAS)* (IEEE, 2017).

[47] Alzuhair, A. Theta phase-specific closed-loop stimulation in implantable neuromodulation devices. Dissertation, University of California, Los Angeles (2019).

[48] Fried I (1999). Cerebral microdialysis combined with single-neuron and electroencephalographic recording in neurosurgical patients: technical note. J. Neurosurg..

[49] Kamousi B (2019). Comparing the quality of signals recorded with a rapid response EEG and conventional clinical EEG systems. Clin. Neurophysiol. Pract..

[50] Chaure FJ, Rey HG, Quian Quiroga R (2018). A novel and fully automatic spike-sorting implementation with variable number of features. J. Neurophysiol..

[51] Moser MB, Rowland DC, Moser EI (2015). Place cells, grid cells, and memory. Cold Spring Harb. Perspect. Biol..

[52] Anumanchipalli GK, Chartier J, Chang EF (2019). Speech synthesis from neural decoding of spoken sentences. Nature.

[53] Pandarinath, C. et al. Latent factors and dynamics in motor cortex and their application to brain–machine interfaces. *J. Neurosci.* **38**, 9390–9401 (2018). 10.1523/JNEUROSCI.1669-18.2018 PMC6209846 30381431

[54] Livezey JA, Glaser JI (2021). Deep learning approaches for neural decoding across architectures and recording modalities. Brief. Bioinform..

[55] Sheth SA (2022). Deep brain stimulation for depression informed by intracranial recordings. Biol. Psychiatry.

[56] Sandha, S. S., Noor, J., Anwar, F. M. & Srivastava, M. Time awareness in deep learning-based multimodal fusion across smartphone platforms. In *IEEE/ACM Fifth International Conference on Internet-of-Things Design and Implementation (IoTDI)* (IEEE, 2020).

[57] Suthana NA (2015). Specific responses of human hippocampal neurons are associated with better memory. Proc. Natl Acad. Sci. USA.

[58] Jenkinson M, Bannister P, Brady M, Smith S (2002). Improved optimization for the robust and accurate linear registration and motion correction of brain images. Neuroimage.

[59] Yushkevich PA (2015). Automated volumetry and regional thickness analysis of hippocampal subfields and medial temporal cortical structures in mild cognitive impairment. Hum. Brain Mapp..

[60] Zhang Y, Brady M, Smith S (2001). Segmentation of brain MR images through a hidden Markov random field model and the expectation-maximization algorithm. IEEE Trans. Med. Imaging.

[61] Kassner, M., Patera, W. & Bulling, A. Pupil: an open source platform for pervasive eye tracking and mobile gaze-based interaction. *Proceedings of the 2014 ACM International Joint Conference on Pervasive and Ubiquitous Computing* 1151–1160. 10.1145/2638728.2641695 (2014).

[62] Whitten TA, Hughes AM, Dickson CT, Caplan JB (2011). A better oscillation detection method robustly extracts EEG rhythms across brain state changes: the human alpha rhythm as a test case. Neuroimage.

[63] Sun, D., Yang, X., Liu, M. Y. & Kautz, J. PWC-Net: CNNs for optical flow using pyramid, warping, and cost volume. *Computer Vision Foundation* https://openaccess.thecvf.com/content_cvpr_2018/papers/Sun_PWC-Net_CNNs_for_CVPR_2018_paper.pdf (2018).

[64] Toglia, M. P. & Battig, W. F. *Handbook of Semantic Word Norms* (Lawrence Erlbaum, 1978).

[65] Srivastava, N., Hinton, G., Krizhevsky, A., Sutskever, I. & Salakhutdinov, R. Dropout: a simple way to prevent neural networks from overfitting. *J. Mach. Learn. Res.* **15**, 1929–1958 (2014).

[66] Solomon EA (2019). Dynamic theta networks in the human medial temporal lobe support episodic memory. Curr. Biol..

[67] Paulk AC (2022). Large-scale neural recordings with single neuron resolution using Neuropixels probes in human cortex. Nat. Neurosci..

