# Supplementary material for: A wearable platform for closed-loop stimulation and recording of single-neuron and local field potential activity in freely moving humans
Source: Nat Neurosci. 2023 Feb 20;26(3):517–27. doi: 10.1038/s41593-023-01260-4 (PMC9991917; doi:10.1038/s41593-023-01260-4)
Supplement: Supplementary file 1 — Supplementary Tables 1–4. [file 41593_2023_1260_MOESM1_ESM.pdf]

nature neuroscience

Technical Report

https://doi.org/10.1038/s41593-023-01260-4

A wearable platform for closed-loop stimulation and recording of single-neuron and local field potential activity in freely moving humans

In the format provided by the
authors and unedited

	Neuro-stack	Nihon Kohden
**Pearson Correlation**	0.9738	
**Activity**	0.0218	0.0222
**Mobility**	0.3435	0.2608
**Complexity**	3.8824	3.9638
**Artifact Spike Count**	1	2
**60 Hz Power**	1.4775×10^{-4}	8.5928×10^{-5}
**Kurtosis**	4.5606	4.8674

Supplementary Table 1 | Neuro-stack and Nihon Kohden comparisons.

Additional metrics were used to compare the Neuro-stack and Nihon Kohden recordings including Pearson correlation, Hjorth parameters (activity, mobility, complexity), artifact spike count, 60 Hz power, and kurtosis.

Participants	Brain Region	Macro-recording	Micro-recording	Macro-stimulation	Walking Task	Verbal Memory Task
1	Left Hippocampus	✓				
2	Left Hippocampus	✓	✓			
3	Left Temporo-Parieto-Occipital	✓	✓			
4	Right Orbitofrontal	✓	✓	✓		
5	Left Entorhinal<br>Left Hippocampus	✓	✓	✓		
6	Left Hippocampus<br>Right Hippocampus	✓	✓		✓	
7	Left Hippocampus<br>Right Hippocampus	✓	✓		✓	✓
8	Left Hippocampus<br>Right Hippocampus	✓	✓		✓	
9	Left Hippocampus<br>Right Hippocampus	✓	✓		✓	
10	Left Hippocampus	✓		✓		
11	Left Hippocampus<br>Anterior Cingulate	✓	✓		✓	
12	Left Hippocampus<br>Right Entorhinal	✓	✓		✓	

Supplementary Table 2 | Participant's electrode localization and experimental tasks completed.

For each participant, electrode localizations (brain regions) are shown as well as whether macro-recording, micro-recording, and/or macro-stimulation was done in conjunction with whether the walking and/or verbal memory task was completed. Brain regions where electrodes were placed were based on clinical criteria and included the left/right hippocampus, left/right entorhinal cortex, anterior cingulate, left temporo-parieto-occipital junction, and right orbitofrontal cortex. A total of eight participants completed the walking task with micro- and macro-electrode recordings to capture single-unit and local field potential activity. The number of channels that were recorded ranged from 2 to 40 (mean: 13.85 channels).

	Blackrock Microsystems	Neuro-stack (4 analog layers)
**Model Name**	CereStim R96 Micro Stimulator	Neuro-stack Stim Engine
**Stimulation Channels**	96	256
**Stimulation Engines**	3	32
**Type of Protection**	Class II	Class II
**Degree of Protection**	Type BF Applied Part	Type BF Applied Part
**Output Voltage**	±4.7 – ±9.5 V	±6 V
**Polarity**	Selectable Anodic or Cathodic First	Selectable Anodic or Cathodic First
**Amplitude**	1 μA – 215 μA, (1 μA)	20 μA – 5,080 μA, (20 μA)
**Pulse Shape**	Rectangular	Custom (Rectangular/Triangle/Sine/Exponential) in 16 steps (8 bits)
**Frequency**	4 Hz – 5 kHz	2.37 Hz – 16.67 kHz
**Pulse Width**	44 μs – 65,535 μs	10 μs – 1,280 μs, (10/20/40/80 μs)
**Interphase Width**	53 μs – 65,535 μs	Interphase: 0 μs – 150 μs, (10 μs)<br>Interpulse: 10 μs – 81.6 ms, (320 μs)<br>Interburst: 81.92 ms - 408.32 ms, (1.28 ms)
**Recommended Electrode Impedance**	< 100 kΩ	< 1 kΩ. High Impedance acceptable
**PC Hardware Interface**	USB A-B cable	USB mini B to type A cable or Wi-Fi
**Stim Manager PC Software Compatibility**	Windows 7 (32/64-bit) compatible	Windows 8.1 or higher
**API**	x86 & x64 library versions	Source and precompiled (ARM, Intel, AMD)
**Analog Resolution**	12 bits	12/21 bits
**External Power Supply**	PMP15M-13 Protek Power Supply<br>AC Input: 100 – 240 Vac, 0.5 – 0.3 A,<br>50 – 60 Hz DC Output: 15 V, 1 A MAX	DC Output Supplies ±6 V<br>USB 5 V
**Cable Connectors**	Samtec MIT-019-02-F-D	Omnetics PS1-16-AA and four 5 × 2 pin connectors per analog layer
**Monitor Connector**	Samtec SMM-109-02-F-D	Mini USB (data/power) or Wi-Fi
**Operating Environment**	10 ˚C to 40 ˚C, 10 to 85% R.H. (non-condensing)	10 ˚C to 40 ˚C
**Storage/Transportation Environment**	-15 ˚C to 60 ˚C, 10 to 85% R.H. (non-condensing), 500 to 1,060 hPA	-15 ˚C to 60 ˚C

Supplementary Table 3 | Comparison of the Neuro-stack and existing Blackrock CereStim stimulation engine.
Shown are detailed features of the stimulation engines, including major advantages of the Neuro-stack which has the ability to customize stimulation waveforms (Pulse Shape, Interphase Width, etc.), a more flexible API (Application Programming Interface) library, and the ability for wireless control (PC Hardware Interface) for ambulatory experiments.

#	Method	Features	Accuracy [%]
1	SVM	Power (TF)	60
2	SVM + TW	Power (TF)	60 – 90
3	SVM + TW + PCA	Power (TF)<br>Phase (TF)	65 – 90
4	GoogleNet + DENSE	Power (TF)<br>Phase (TF)	69.40 – 96.87
5	DENSE	Raw Data	72.1 – 81.2
6	CNN2D + DENSE	Power (TF)<br>Phase (TF)	63.4 – 75.0
7	LSTM/GRU + DENSE	Raw Data	75.6 – 88.3
8	CNN1D + DENSE	Raw Data	72.1 – 87.9
9	GP + DENSE	Raw Data	40 – 60
10	CNN1D + LSTM + DENSE	Raw Data	88.3 – 99.5

Supplementary Table 4 | Machine learning methods used for decoding memory using the Neuro-stack.

Accuracy comparison of the different algorithms used for decoding verbal memory performance from neural activity in real-time using the Neuro-stack. SVM: support vector machine; TW: time-windowing; PCA: principal component analysis; GoogleNet: a large Google's neural network, pretrained on time-frequency images of the electrocardiogram signals; Dense: fully connected neural network layer; CNN2D: 2D convolutional neural network layer; LSTM: long-short term memory neural network layer; GRU: gated recurrent unit neural network layer; CNN1D: 1D convolutional neural network layer; GP: gaussian process. Power (TF) and Phase (TF) refer to chunks of either power or phase time-frequency (TF) representations of the signal. Raw data refers to neural data that is minimally processed (downsampled) in the time-domain and fed directly into the decoding algorithm. See Online Methods section for more details.
