# Supplementary material for: A wearable platform for closed-loop stimulation and recording of single-neuron and local field potential activity in freely moving humans
Source: Nat Neurosci. 2023 Feb 20;26(3):517–27. doi: 10.1038/s41593-023-01260-4 (PMC9991917; doi:10.1038/s41593-023-01260-4)
Supplement: Supplementary file 2 — Reporting Summary [file 41593_2023_1260_MOESM2_ESM.pdf]

nature portfolio

Corresponding author(s): Nanthia Suthana

Last updated by author(s): Nov 2, 2022

Reporting Summary

Nature Portfolio wishes to improve the reproducibility of the work that we publish. This form provides structure for consistency and transparency in reporting. For further information on Nature Portfolio policies, see our Editorial Policies and the Editorial Policy Checklist.

Statistics

For all statistical analyses, confirm that the following items are present in the figure legend, table legend, main text, or Methods section.

n/a	Confirmed	
☐	☒	The exact sample size (*n*) for each experimental group/condition, given as a discrete number and unit of measurement
☐	☒	A statement on whether measurements were taken from distinct samples or whether the same sample was measured repeatedly
☐	☒	The statistical test(s) used AND whether they are one- or two-sided<br>*Only common tests should be described solely by name; describe more complex techniques in the Methods section.*
☐	☒	A description of all covariates tested
☐	☒	A description of any assumptions or corrections, such as tests of normality and adjustment for multiple comparisons
☐	☒	A full description of the statistical parameters including central tendency (e.g. means) or other basic estimates (e.g. regression coefficient) AND variation (e.g. standard deviation) or associated estimates of uncertainty (e.g. confidence intervals)
☐	☒	For null hypothesis testing, the test statistic (e.g. *F*, *t*, *r*) with confidence intervals, effect sizes, degrees of freedom and *P* value noted<br>*Give P values as exact values whenever suitable.*
☒	☐	For Bayesian analysis, information on the choice of priors and Markov chain Monte Carlo settings
☒	☐	For hierarchical and complex designs, identification of the appropriate level for tests and full reporting of outcomes
☒	☐	Estimates of effect sizes (e.g. Cohen's *d*, Pearson's *r*), indicating how they were calculated

Our web collection on statistics for biologists contains articles on many of the points above.

Software and code

Policy information about availability of computer code

Data collection	Neuro-stack Integrated Circuits (related papers stated in the manuscript), Custom printed circuit boards (Altium Designer 14.0); C++, Bash, Python (3.6.9), Pupil Capture (Pupil Labs, v2.3), Xilinx ISE 14.2 (Hardware description language: Verilog), Xcode 11.2.1 (Language: Swift 5.0.1), Microsoft Visual Studio 2017 (Language: C#, Universal Windows Platform development), Keras/TensorFlow and TensorFlow Lite 2.2. Operating systems: Windows 8, MacOS 10.13-11, Linux Mendel distribution, iOS 11.
Data analysis	MATLAB (2021b; Wavelet, Signal Processing, Statistics and Machine Learning Toolboxes), Python (3.6.9), Pupil Player (Pupil Labs, v2.3), BrainLab, FSL FLIRT, FSL FAST, ASHS. Operating systems: MacOS 11.

For manuscripts utilizing custom algorithms or software that are central to the research but not yet described in published literature, software must be made available to editors and reviewers. We strongly encourage code deposition in a community repository (e.g. GitHub). See the Nature Portfolio guidelines for submitting code & software for further information.

Data

Policy information about availability of data

All manuscripts must include a data availability statement. This statement should provide the following information, where applicable:

- Accession codes, unique identifiers, or web links for publicly available datasets
- A description of any restrictions on data availability
- For clinical datasets or third party data, please ensure that the statement adheres to our policy

Data is available upon reasonable request

Human research participants

Policy information about studies involving human research participants and Sex and Gender in Research.

Reporting on sex and gender	Twelve participants (mean age 24 years, three males and nine females) took part in this study. Sex or gender information was not considered nor used for study design or experimental purposes, because the goal of the study was in-vivo validation of the proposed technical system, rather than an investigation of behavioral/cognitive effects in or differences between individuals. Information regarding the participants' sex or gender data was collected only for descriptive purposes during the recruitment process, according to an approved protocol.
Population characteristics	Twelve participants (mean age 24 years, three males and nine females) with pharmaco-resistant epilepsy and implanted depth electrodes for clinical monitoring in the hospital took part in this study. Electrode placements were determined solely based on clinical criteria.
Recruitment	During or before the scheduled hospital stay, participants were contacted in-person or via phone/e-Mail. They were given a detailed explanation of the study and were asked whether they would be willing to volunteer and consent to participate in the study. Participants were not offered compensation for their involvement in this research. Given that some of our experiments involved stimulation and physical walking, the participants were part of this experimental groups were always tested a day before or on the day of deplantation, while they were on medication. The participants were always informed in advance, during consent process, and during the experimental session about the experimental procedure. For the experiment with freely-walking participants, authors, in collaboration with the clinical team, specifically asked for participants, who were in good physical condition (i.e., able to walk freely) at the time of the experiment to avoid unnecessary risks (e.g., falling) in participants that were physically less fit. Brain stimulation was performed only in adult participants (>21 years of age) for ethical and safety reasons. However, these criteria were unlikely to impact the Neuro-stack's ability to record or stimulate, nor to substantially change the data and conclusions presented in the manuscript. Allocation to other experimental groups (stationary recording and verbal memory task) was not based on specific selection criteria.
Ethics oversight	All participants volunteered for the study by providing informed consent according to a protocol approved by the UCLA Medical Institutional Review Board (IRB)

Note that full information on the approval of the study protocol must also be provided in the manuscript.

Field-specific reporting

Please select the one below that is the best fit for your research. If you are not sure, read the appropriate sections before making your selection.

☒ Life sciences ☐ Behavioural & social sciences ☐ Ecological, evolutionary & environmental sciences

For a reference copy of the document with all sections, see nature.com/documents/nr-reporting-summary-flat.pdf

Life sciences study design

All studies must disclose on these points even when the disclosure is negative.

Sample size	Twelve participants (mean age 24 years, three males and nine females) with pharmaco-resistant epilepsy and implanted depth electrodes for clinical monitoring in the hospital took part in this study. No statistical methods were used to predetermine sample size. Since the main aim of this study was the validation of recording and stimulation capabilities of our developed Neuro-stack system, we chose sample sizes that are similar to (or larger than) previous studies, where similar recordings were performed with other similar technical systems (for example: Gilron et al., 2021, Paulk et al., 2022).
Data exclusions	Data from each participant were analyzed separately, but not all results or redundant conclusions about recording or stimulation signal quality were presented for each participant. Data from two participants (not included in the manuscript), who attempted to perform the stationary verbal memory task, were excluded from analysis, these participants did not complete the whole task procedure, but asked to stop the experiment after insufficient number of trials.

Replication	All methods used to perform this study are detailed in the Methods section. The Reproducibility subsection, within the Methods section, contains a statement about the reproducibility of this work. Data were analyzed for each individual participant separately.<br>Selection of participants, assignment of participants to different experimental tasks (i.e., stationary recording, recording during ambulatory walking, stationary stimulation, or verbal memory), sample sizes per experimental tasks, and number of replications and task durations per participant were determined in close collaboration with the clinical stuff and were primarily based on the participant's clinical, cognitive, and physical condition. For example, only participants in good physical condition who were able to walk freely were asked to participate in the ambulatory walking experiment.<br>Consequently, stationary recording, ambulatory recording, stationary stimulation, and stationary verbal memory task were successfully tested in 12, 6, 3, and 1 participants respectively. Hence, with the exception of the verbal memory task (described below), all experimental tasks were replicated in multiple participants. All attempts of stationary recordings and stimulation were of high quality and deemed successful. As reported in the manuscript, mobile recordings in the first 4 participants were used to determine and adjust technical parameters and the setup, and then remained constant for successful recordings in two more participants.<br>The stationary verbal memory task was successfully completed in one participant, with two more attempts with participants (excluded from analysis) who did not wish to continue with the experiment before the artificial neural networks could be trained to provide meaningful results. While the main goal of this work was the technical system's human in-vivo validation rather than empirically characterizing neural effects related to participant's behavior and cognition, the aim of neural recordings during the verbal memory task was to showcase the technical feasibility of an automatic real-time analysis of the neural data, as an example use case of the Neuro-stack. This technical feasibility could be successfully demonstrated in one participant; however, behavioral/cognitive effects and conclusions regarding the verbal memory task were not replicated in this study and warrant future investigation.<br>In-vitro recording and stimulation results were replicated and recorded 3 times independently prior to in-vivo experiments, in addition to numerous other in-vitro tests carried out as part of acquiring IRB approval, and as part of previous publications (Rozgic et al., 2017; Rozgic., et al 2018; Basir-Kazeruni et al., 2017; Chandrakumar et al., 2017; Alzuhair et al., 2018; Alzuhair et al., 2019). The delay measurements were performed twice independently with multiple measurements (50) during each and as part of previous publications (Alzuhair et al., 2019).
Randomization	The Neuro-stack setup was the same for all tested participants, but the protocols differed for recording and stimulation tests as well as for resting state, stationary, and ambulatory tests. Recording and stimulation functionality were tested in twelve and three participants, respectively. During these tests, we varied, depending on the given participant's clinical, cognitive, and physical condition (in collaboration with their clinical team), the number of channels to record from as well as stimulation parameters (current, amplitude, etc.) in order to showcase stimulation programmability. The stationary verbal memory task was performed in one participant, and the ambulatory walking task was performed in six participants. Some of the participants were involved in multiple tests as stated in the manuscript.<br>Participants were asked to perform different experimental tasks (i.e., stationary recording, recording during ambulatory walking, stationary stimulation, or verbal memory), based on their physical, cognitive, and clinical condition. Thus, selection of participants, or assignment to different experimental tasks, sample sizes, and replication decisions were determined in close collaboration with the clinical staff and were primarily based on the participant's condition. For example, stationary stimulation required the presence of neurologists on-site for safety reasons (as per IRB and safety requirements), and only participants in good physical condition who were able to walk safely were asked to participate in the ambulatory walking task.<br>For these reasons, full randomization and random assignment of participants to experimental tasks was not possible in this study. Given that the main aim of this work was the validation of the Neuro-stack recording and stimulation system, rather than empirical conclusions regarding cognitive or behavioral effects in individuals, it is the authors' opinion that this non-random assignment of participants to experimental tasks had minimal or no impact on the relevance of the work.
Blinding	We tested a group of participants with pharmaco-resistant epilepsy, while they were implanted with depth electrodes for clinical monitoring. All experimenters were aware of this fact and were not blinded with regards to the participant's condition. Participant's condition and clinical setting influenced the allocation into the experimental groups and the Neuro-stack's setup, which is why blinding was not possible.

Reporting for specific materials, systems and methods

We require information from authors about some types of materials, experimental systems and methods used in many studies. Here, indicate whether each material, system or method listed is relevant to your study. If you are not sure if a list item applies to your research, read the appropriate section before selecting a response.

Materials & experimental systems

n/a	Involved in the study
☒	☐ Antibodies
☒	☐ Eukaryotic cell lines
☒	☐ Palaeontology and archaeology
☒	☐ Animals and other organisms
☒	☐ Clinical data
☒	☐ Dual use research of concern

Methods

n/a	Involved in the study
☒	☐ ChIP-seq
☒	☐ Flow cytometry
☐	☐ MRI-based neuroimaging

Magnetic resonance imaging

Experimental design

Design type	MRI was used only to determine the localization of electrode contacts within the brain.

Design specifications	MRI was used only for electrode contact localization. Participants did not perform any experimental task in this study during MRI scanning.
Behavioral performance measures	No behavioral performance measures were acquired or derived.

Acquisition

Imaging type(s)	Structural
Field strength	3 Tesla
Sequence & imaging parameters	Standard T1- and T2-weighted sequences.
Area of acquisition	Whole brain

Diffusion MRI ☐ Used ☒ Not used

Preprocessing

Preprocessing software	BrainLab, FSL (FMRIB Software Library) FLIRT and FAST, Automatic Segmentation of Hippocampal Subfields (ASHS).
Normalization	MRI images were not normalized.
Normalization template	MRI images were not normalized.
Noise and artifact removal	No noise or artifact removal procedures were used.
Volume censoring	No volume censoring were used.

Statistical modeling & inference

Model type and settings	No modeling of MRI data was performed.
Effect(s) tested	MRI was used only to determine the localization of electrode contacts within the brain, and not for testing.

Specify type of analysis: ☒ Whole brain ☐ ROI-based ☐ Both

Statistic type for inference (See Eklund et al. 2016)	No statistical analyses were performed using MRI data.
Correction	No correction methods were applied.

Models & analysis

n/a	Involved in the study
☒	☐ Functional and/or effective connectivity
☒	☐ Graph analysis
☒	☐ Multivariate modeling or predictive analysis
