# Supplementary material for: A wearable platform for closed-loop stimulation and recording of single-neuron and local field potential activity in freely moving humans
Source: Nat Neurosci. 2023 Feb 20;26(3):517–27. doi: 10.1038/s41593-023-01260-4 (PMC9991917; doi:10.1038/s41593-023-01260-4)
Supplement: Supplementary file 3 — Editorial Assessment Report. [file 41593_2023_1260_MOESM3_ESM.pdf]

Contents of this report

1. **Manuscript details**: overview of your manuscript and the editorial team.
2. **Review synthesis**: summary of the reviewer reports provided by the editors.
3. **Editorial recommendation**: personalized evaluation and recommendation from all 3 journals.
4. **Annotated reviewer comments**: the referee reports with comments from the editors.
5. **Open research evaluation**: advice for adhering to best reproducibility practices.

About the editorial process

Because you selected the **Nature Portfolio Guided Open Access** option, your manuscript was assessed for suitability in three of our titles publishing high-quality work in your field of research. More information about Guided Open Access can be found here.

Collaborative editorial assessment

Your editorial team discussed the manuscript to determine its suitability for the Nature Portfolio Guided OA pilot. Our assessment of your manuscript takes into account several factors, including whether the work meets the technical standard of the Nature Portfolio and whether the findings are of immediate significance to the readership of at least one of the participating journals in the Guided OA pilot.

Peer review

Experts were asked to evaluate the following aspects of your manuscript:

- **Novelty** in comparison to prior publications;
- **Likely audience** of researchers in terms of broad fields of study and size;
- **Potential impact** of the study on the immediate or wider research field;
- **Evidence** for the claims and whether additional experiments or analyses could feasibly strengthen the evidence;
- **Methodological detail** and whether the manuscript is reproducible as written;
- Appropriateness of the **literature review**.

Editorial evaluation of reviews

Your editorial team discussed the potential suitability of your manuscript for each of the participating journals. They then discussed the revisions necessary in order for the work to be published, keeping each journal's specific editorial criteria in mind.

Journals in the Nature portfolio will support authors wishing to transfer their reviews and (where reviewers agree) the reviewers' identities to journals outside of Springer Nature.
If you have any questions about review portability, please contact our editorial office at guidedoa@nature.com.

Manuscript details

Tracking number	Submission date	Decision date	Peer review type
GUIDEDOA-22-00448	Mar 24, 2022	May 11, 2022	Single-blind

Manuscript title

A wearable platform for closed-loop stimulation and recording of single-neuron and local field potential activity in freely-moving humans

Preprint: link if available

Author details

Nanthia Suthana

Affiliation: University of California, Los Angeles

Editorial assessment team

**Primary editor**	**Luis Mejia**<br>Home journal: ***Nature Neuroscience***<br>ORCID: **0000-0001-5439-6803**<br>Email: **luis.mejia@us.nature.com**
**Other editors consulted**	**David Rowland**<br>Home journal**: *Nature***<br>ORCID**: 0000-0002-2735-2730**<br><br>**Fiona Carr**<br>Home journal**: *Nature Communications***<br>ORCID**: 0000-0002-2957-1371**
**About your primary editor**	Luis joined Nature Neuroscience in 2018. He received his Ph.D. in Neuroscience from Harvard Medical School, followed by postdoctoral research at Cold Spring Harbor Laboratory in the lab of Bo Li, where he studied orbitofrontal-striatal projection neurons in value- and valence-based decisions and behaviors in mice, using in vivo optogenetics and calcium imaging. His research interests include

	systems and circuits neuroscience, and in vivo imaging and neuroscience methods. Luis is based in the New York office.

Editorial assessment and review synthesis

**Editor's summary and assessment**	The authors develop a multi-channel implantable/wearable device for programmable closed-loop stimulation and recording (single unit/LFP and iEEG) in stationary or walking humans, called 'Neuro-stack'. Further, in one patient, the authors record activity in real-time and decode performance on a verbal memory task. The editors found the report to be interesting as a proof-of-principle for portable multi-channel recording and stimulation in awake humans, promising for its ability to isolate single units and its programmable capability. The editors also wondered whether the broad advance demonstrated at present was sufficient, though novelty was deemed sufficient for external review.<br><br>As part of the Guided Open Access pilot, editors from Nature, Nature Neuroscience and Nature Communications have discussed the reviewer reports and the manuscript's suitability for the journals. After careful evaluation, our editorial recommendation is to revise the manuscript and submit back through the Guided Open Access submission portal for consideration at *Nature Neuroscience* or *Nature Communications*.
**Editorial synthesis of reviewer reports**	Your manuscript has been seen by 3 reviewers with expertise in recording and stimulation approaches in humans. While the reviewers find the work to be of interest, they have raised a number of substantial conceptual, technical and analytical critiques/questions.<br><br>To be considered further at *Nature Neuroscience*, or *Nature Communications*, you would need to fully address all the reviewer concerns and comments, including those technical points regarding further quantifications and analyses.

Editorial recommendation

Nature<br><br>Revision not invited	Following editorial assessment of the paper and reviewer reports it was felt the advance is not sufficient for further consideration at Nature.
Nature Neuroscience<br><br>Major revisions	The editors would expect to see all of the referees' points addressed with additional data/analyses.
Nature Communications<br><br>Major revisions	The editors would expect to see all of the referees' points addressed with additional data/analyses.

Next steps

**Editorial recommendation 1:**	Our top recommendation is to revise and resubmit your manuscript to *Nature Neuroscience.* We feel that the additional data requested are reasonable.
**Editorial recommendation 2:**	You may also choose to revise and resubmit your manuscript to *Nature Communications.* This option might be best if the requested data revisions are not possible/feasible at this time.
Note	As stated on the previous page, *Nature* is not inviting a revision at this time. Please keep in mind that the journal will not be able to consider any appeals of their decision through Guided Open Access.

Revision

To follow our recommendation, please upload the revised manuscript files using **the link provided in the decision letter**.

Revision checklist

- ☐ Cover letter, stating to which journal you are submitting
- ☐ Revised manuscript
- ☐ Point-by-point response to reviews
- ☐ Updated Reporting Summary and Editorial Policy Checklist
- ☐ Supplementary materials (if applicable)

Submission elsewhere

If you choose not to follow our recommendations, you can still take the reviewer reports with you.

Option 1: Transfer to another Nature Portfolio journal
Springer Nature provides authors with the ability to transfer a manuscript within the Nature Portfolio, without the author having to upload the manuscript data again. To use this service, **please follow the transfer link provided in the decision letter.** If no link was provided, please contact guidedOA@nature.com.

> *Note that any decision to opt in to In Review at the original journal is not sent to the receiving journal on transfer. You can opt in to In Review at receiving journals that support this service by choosing to modify your manuscript on transfer.*

Option 2: Portable Peer Review option for submission to a journal outside of Nature Portfolio
If you choose to submit your revised manuscript to a journal at another publisher, we can share the reviews with another journal outside of the Nature Portfolio if requested. You will need to request that the receiving journal office contacts us at guidedOA@nature.com. We have included editorial guidance below in the reviewer reports and open research evaluation to aid in revising the manuscript for publication elsewhere.

Annotated reviewer reports

The editors have included some additional comments on specific points raised by the reviewers below, to clarify requirements for publication in the recommended journal(s). However, please note that all points should be addressed in a revision, even if an editor has not specifically commented on them.

Reviewer #1 information	
**Expertise**	Recording and stimulation approaches in humans, devices
**Editor's comments**	The reviewer has provided an overall positive assessment of the paper, but has raised important conceptual comments (e.g. pertaining to stated novelty) and a number of important points regarding the technical aspects.

Reviewer #1 comments	
**Section**	**Annotated Reviewer Comments**
**Remarks to the Author: Overall significance**	General overview:<br>This is an interesting, well written paper describing a novel device, designed and built by these authors, for use in invasive human brain recording, the "Neurostack". It is designed to attach to externalized leads, either standard SEEG leads from which LFPs are recorded, or microwires through which single units may be resolved. The primary innovation over existing high channel recording systems (used by these authors and others in epilepsy monitoring units, is that the new system is small enough to be worn while the patient is freely moving). The primary innovation over existing fully implantable brain sensing/stimulation devices is the high channel count, high sampling rates, and ability to record single units. Thus this paper is probably the first demonstration of multiple single unit recording in freely moving humans, and as such it does represent an important technical achievement.<br>One point about device novelty that is overemphasized at several points in the manuscript is that the device has a "full duplex" capability. While an important feature to allow fully closed loop stimulation, there have been a number of other fully implantable full duplex devices implanted in humans (described further below). Thus the novelty of this device really lies in its capability to perform single unit recording in ambulatory humans, which no existing device can do. However (see further below), the authors do not clearly explain why it is that single unit capability provides clinical and systems neuroscience opportunities that are not afforded by field potential approaches. This work is an important technical step in the transition from large bedsite recording systems for single unit recording, to, eventually, fully implantable systems that can transmit single unit data wirelessly. An important element of this paper that is not really well described, is that it

represents device development and testing primarily in an academic medical center, with full access to human subjects, rather than exclusively in a device company where development occurs without constant interaction with clinicians or availability of human subjects. This is unusual and noteworthy and should be described. How were the circuit boards fabricated? How was device software written and documented according to good manufacturing practice standards? Device development occurring primarily in academia is a new and important paradigm and some mention of the inherent challenges, and how the authors overcame these, would add general interest to the paper.
Specific comments:
Introduction:
The authors imply that single unit recording in the human brain is a critical need but do not justify it. There has been surprising progress in understanding brain disorders using field potentials as probes of oscillatory synchronization or in the study of evoked potentials. One could even argue that it would take thousands of simultaneous unit recordings to probe phenomena related to neural synchronization and that this could better be done with LFP recordings. Certainly many arguments exist for the utility of unit recording in systems neuroscience, compared to other methods, but those should be made more explicitly in either the introduction, discussion or both.

"Finally, an additional impediment in developing new responsive neurostimulation treatments is the lack of a customizable bi-directional interface that can record simultaneously with stimulation (full-duplex) and thus "talk" with the brain at the speed of behavior and cognition" – this is a bit misleading. The first responsive neurostimulator, RNS from Neuropace, is indeed "half duplex" but since then there have been at least six different full duplex devices implanted in humans: Activa PC+S, RC+S, and Percept (Medtronic), the PINS DBS device from China, AlphaDBS from Nevronika, and the PicoStim from Bio-induction.

Further down in introduction: "Existing closed-loop implantable technologies also lack fullduplex ability, which allows for simultaneous stimulation and recording of neural tissue inclusive of unit and LFP activity". Again this is not the case, the above 6 devices implanted in humans are full duplex, albeit with important challenges from stimulation artifact and various restrictions on their availabilty. Of note the ability to provide phase locked stimulation in a mobile person is important feature of their new device so might deserve more emphasis in the intro than the full duplex aspect. No existing fully implantable can do this, at least in fully embedded mode (but can do so in a distributed mode using an external computer)

Results:
"Further, wide-band sensing from up to 32 monopolar or bipolar recordings at up to 38.6 kHz allows for the recording of single-unit and LFP activity simultaneously" – the "wide band" distinction between these and the full set of 256 channel

	recordings is not clear. In what sense are the 256 channels capable of wide band recording?<br><br>Figure 1g- this does not look like a typical power spectrum of field potential activity. Is it many power spectra superimposed? Is it from a brain structure where oscillatory activity is expected and if so is it present? The spectrograms in 1f suggest theta activity but that is not really apparent in g.<br><br>**Nature Neuroscience and Nature Communications would expect that you appropriately resolve all the points and requests.**
**Remarks to the Author: Impact**	the paper is interesting and well done as a new technology demonstration. its seems most appropriate for Nature biotechnoloy or Nature Biomedical Engineering.
**Remarks to the Author: Strength of the claims**	the work is convincing in itself, with respect to the development a device for human brain recording that both has many sophisticated capabilities, and is wearable on the patient while ambulating. The most compelling technology would be one with high bandwidth unit recording that is fully implantable and totally wireless, but such a device would be a new leap that is well beyond that described here and not in the scope of the work presented.
**Remarks to the Author: Reproducibility**	the authors do reproduce important single unit results on memory encoding (previously published by some of these same authors). The device is described in sufficient detail that it should be reproducible in principle.

Reviewer #2 information

**Expertise**	Recording and stimulation approaches in humans, clinical, memory, devices
**Editor's comments**	The reviewer has provided an overall positive assessment of the paper, but has raised important conceptual questions and a number of important points regarding the technical/ analytical aspects.

Reviewer #2 comments

Section	Annotated Reviewer Comments
**Remarks to the**	Topalovic et al. present an impressive new recording and stimulation system that

**Author: Overall significance**	allows the recording of single neuron activity and local field potential activity in freely moving humans implanted with electrodes for localization of epileptic seizures for the first time.<br><br>Strengths include the highly sophisticated and novel piece of hardware that is described, with extensive engineering efforts that went into high channel counts, stimulation capability, closed-loop processing, edge computing to process pre-trained AI models, low power consumption per channel and low footprint to make it portable. These capabilities are highly impressive and address the major open need of having high-quality high-channel count recordings from freely moving human beings (in particular single-unit, which has never been done). As a proof of principle, recordings were done in 8 freely moving subjects. On-chip decoding/learning in the verbal memory task is demonstrated. The built-in stimulation capability is significant, in particular the demonstration that the stimulus artifacts do not drive amplifiers out of range, allowing closed-loop paradigms that require recordings during stimulation. The validation performed in terms of comparing signals to those recorded simultaneously with a clinical system is a major plus.<br><br>Weaknesses: First, there is no 'proof of principle' result that would indicate that the recorded activity during freely moving behavior was related to a task, stimulus or other types of neural activity in some form, leaving it unclear whether the different data streams can be brought together to conduct such an analysis in the face of all the challenges present. For example, was theta elevated during movement? Second, it remains unclear to what extent the motion artifacts degraded ability to interpret neural activity (but this is addressable through quantification, see below).<br><br>Overall, this paper presents very significant and very novel progress relative to the state of the art and the first demonstration of ability to record single units in freely moving humans, which is a major advance. In my opinion, the weaker parts are the on-chip learning in a single patient (see below, not clear how much above chance its performance is) and the closed-loop stim that is done only in-vitro, which seem a bit of a distraction relative to the major novelty of the recording in moving humans part (i.e. Fig 2) and the significant stimulation capability that is also tested in-vivo (Fig. 3).<br><br>**Nature Neuroscience and Nature Communications would expect that you appropriately resolve all the points and requests.**
**Remarks to the Author: Impact**	Yes, I do think this paper will influence thinking in the field -- towards achieving single neuron/LFP recordings from freely moving subjects, at least in the confines of a clinic setting while implanted.

**Remarks to the Author: Strength of the claims**	Strength of claims: the claims related to ability of recording LFP and single-neurons at rest and movement, as well as open-loop stimulation are convincing, with the need for some additional quantification as stipulated below. Fig. 4 i have questions about, see below.<br><br>Major issues:<br>1. The ability to record single neurons and LFP during free moving behavior is a major novelty and advance of this paper. Therefore, I would ask the authors to quantify these statements. What is the yield, quality, and stability of the isolated neurons during free movement vs. at rest? How does the background noise compare? Same question for LFP. The example units shown in Fig. 2f-g look very impressive, but I could not see any further quantification of single unit isolation quality across all the participants that walked freely.<br><br>2. Motion artifacts. Various statements are made regarding the presence of motion artifacts (i.e. Fig. S2) and the degrees of success in removing them. Can these statements be quantified somehow? i.e. do the remaining artifacts allow the interpretation of LFP during walking (such as theta) or units? In Fig 2c, what aspect of what is shown are motion artifacts vs. real signal? Would be useful to mark. For example I see sharp high-frequency transients that are visible on all channels, are those motion artifacts?<br><br>3. Verbal memory task. This is a nice proof of principle, but I struggle with a few issues. First it isn't clearly described what is predicted. I assume it was encoding success (i.e. during encoding), but this should be indicated clearly. Were classes balanced for training? Second, what is chance performance? The subject recalled 38.9% of the words shown (Fig S6b). Given this, is what is shown above chance performance? A decoder that would always say "forget" would get an accuracy of 61.1% for this patient; the decoder achieved ~72%, is that significant?. For Fig 4, Fig. S6 c/d it is not clear what would be expected after randomly scrambling labels. Third, what has been observed in many studies is that 'encoding success' is predicted by lower theta power relative to that of encoding failure (Solomon et al. 2019, Current Biology). Is this what the authors also find? Fig. 4f,g seems to indicate the opposite assuming red=positive, but please clarify. Forth, Fig. 4c bottom its not clear to me what marks the different 'blocks', please mark. Is the performance achieved significantly larger than chance (shuffle control) at different points of time ? The F1 score can be misleading- would be more informative to plot hits/false alarms.<br><br>4. Closed-loop stimulation. To what extent can the closed-loop stimulation capability work in the presence of stim and motion artifacts? This could be addressed using the existing data by examining the accuracy of a power threshold or phase-detector that would putatively trigger the stimulation.

	Minor issues and comments:<br>1. "Behaviors that are studied in animal neurobiology are done almost exclusively in freely-moving animals" large majority of studies have been done in head-fixed monkeys and rats<br>2. "suggest that deviation of underlying neural activity is not the only cause of artifact waveform uncertainty" -> what does this mean?<br>3. "256-channel (128 monopolar/bipolar macro recordings)" -> wasn't clear to me what this meant, so if monopolar, there are 256 recording channels possible for iEEG?<br>4. Are there mechanisms in place for other researchers to be able to build this piece of hardware for themselves? It seems that given that everything is based on custom made chips, this would not be possible? This is of course inevitable and expected for custom hardware development, but it would be helpful to be clear about what could or could not be expected in terms of sharing.<br><br>**Nature Neuroscience and Nature Communications would expect that you appropriately resolve all the points and requests.**
**Remarks to the Author: Reproducibility**	See my minor issue #4<br><br>**Nature Neuroscience and Nature Communications would expect that you appropriately resolve all the points and requests.**

Reviewer #3 information

**Expertise**	Recording and stimulation approaches in humans, clinical
**Editor's comments**	The reviewer has provided an overall positive assessment of the paper, but has raised important questions and a number of important points regarding the technical/ analytical aspects.

Reviewer #3 comments

Section	Annotated Reviewer Comments
**Remarks to the Author: Overall significance**	In this report, Topalovic et al present the findings with a state of the art neurophysiological recording device that was developed partially as part of the DARPA SUBNETS and RAM programs. The authors have successfully shown proof of concept data from real life naturalistic recordings as well as during a controlled memory task recording 256 channels of iEEG activity and 32

	channels of "single unit" activity. They also show impressive data from macro-stimulation (up to 32 channels simultaneously) and neural network model for predicting theta bursts during memory retrieval.
**Remarks to the Author: Impact**	As a clinician scientist, I see this device to be a game changer in the field of Neuromodulation for various clinical settings, most clearly for patients with Epilepsy in whom this device was first tested. The device also represents a major leap in science where we can record neuronal and neural activity during naturalistic settings. One of the key stake holders in this project, Dr Nanthia Suthana has already paved the way for impactful research in this field using a much more limited RNS (NeuroPace Inc) technology.<br><br>I see a great future for this device because of its flexible programmability, combined wireless and wearable components, and the capability of recording and stimulating in closed loop manner in many channels simultaneously with sine waves (close to natural brain rhythms). This device will completely revolutionize the clinical and basic science fields of intracranial electrophysiology and neuromodulation. I congratulate the authors for the development of this great tool for science
**Remarks to the Author: Strength of the claims**	Minor Suggestions:<br>1) In Line 137: The Neuro-stack has built-in (hardware) oscillation power detection and thus the ability to trigger stimulation at a predefined phase of an oscillation.... Does the device detect ALL bands of oscillatory activity or just theta as shown in the following lines ? Pls clarify<br><br>2) For epilepsy clinicians it is important to show example of actual EEGs recorded with this device. I do not think the current figures do the job. Critical readers will question how much muscle and movement noise will impact the EEG waveforms. Fig 1e seems a cherry picked segment but yet with too much higher frequency signal in the muscle artifact range (40-100HZ). Need to extend the X-axis scale to show a couple of seconds of actual EEG instead of jamming tens of seconds.<br>Similarly, Fig1 f Shows only (0.1–32 Hz) and ignores the signal frequency range for muscle artifact. In Figure 1g, Neuro-Stack exceeds Nihon Kohden in high frequency signal.<br>Also a proper comparison of two EEG signals ought to be provided using unbiased and non-cherry picked signals: Please see Kamousi et al (Clinical Neurophysiology Practice (2019) for how unbiased comparison of two EEG signals can be done. I feel that these specific additional experiments would strengthen the case for NeuroStack.<br><br>**Nature Neuroscience and Nature Communications would expect that you appropriately resolve all the points and requests.**

**Remarks to the Author: Reproducibility**	

Open research evaluation

General information

Guidelines for Transparency and Openness Promotion (TOP) in Journal Policies and Practices ("TOP Guidelines")

The recommendations and requests in the table below are aimed at bringing your manuscript in line with common community standards as exemplified by the TOP Guidelines. While every publisher and journal will implement these guidelines differently, the recommendations below are all consistent with the policies at Nature Portfolio. In most cases, these will align with TOP Guidelines Level 2.

FAIR Principles

The goal of the recommendations in the table below related to **data or code** availability is to promote the FAIR Guiding Principles for scientific data management and stewardship (*Scientific Data* **3**: 160018, 2016). The FAIR Principles are a set of guidelines for improving 4 important aspects of digital research objects: **F**indability, **A**ccessibility, **I**nteroperability and **R**eusability.

ORCID

ORCID is a non-profit organization that provides researchers with a unique digital identifier. These identifiers can be used by editors, funding agencies, publishers, and institutions to reliably identify individuals in the same way that ISBNs and DOIs identify books and articles. Thus the risk of confusing your identity with another researcher with the same name is eliminated. **The ORCID website** provides researchers with a page where your comprehensive research activity can be stored.

Springer Nature collaborates with the ORCID organization to ensure that your research contributions (as authors and peer reviewers) are correctly attributed to you. Learn more at https://www.springernature.com/gp/researchers/orcid

Data availability
**Data Availability Statement**
Many journals, including all Nature Portfolio journals, require a Data Availability Statement in the manuscript as a condition of publication. The Data Availability Statement should be as detailed as possible and include accession codes or other unique IDs for deposited data, information about where source data can be found, and specify any restrictions to data access that may apply. At a minimum, the statement should indicate that data are available upon request and explain how data access can be granted. If data access is not possible, the reasons for this must be made clear in the Data Availability Statement.<br><br>More information about the Nature Portfolio data availability policy can be found here: https://www.nature.com/nature-portfolio/editorial-policies/reporting-standards#availability-of-data<br><br>Additional information about Data Availability Statements and Springer Nature's data policies are available here: http://www.springernature.com/gp/authors/research-data-policy/data-availability-statements/12330880
Thank you for including a Data Availability statement in your manuscript. You have stated that data are only available upon request. These statements are strongly discouraged by the research community as they do not make it clear which data are available and under what conditions. The data availability statement must make the conditions of access to the data underlying the study transparent to readers. Please explicitly state if and how readers may access the individual datasets reported in the study, including any unique identifiers or URLs and any restrictions to access that apply.<br><br>See here for more information about Data Availability Statements and Springer Nature's data policies: http://www.springernature.com/gp/authors/research-data-policy/data-availability-statements/12330880
**Other data requests**

In line with community standards regarding open research, Springer Nature strongly supports data sharing and believes that all datasets on which the conclusions of the paper rely should be available to readers. We encourage authors to ensure that their datasets are either deposited in publicly available repositories (where available and appropriate) or presented in the main manuscript or additional supporting files whenever possible.<br><br>To learn more about data sharing and recommended data repositories, please see https://www.springernature.com/gp/authors/research-data-policy/repositories/12327124
All source data underlying the graphs and charts presented in the main figures must be made available as Supplementary Data (in Excel or text format) or via a generalist repository (eg, Figshare or Dryad). This is mandatory for publication in a Nature Portfolio journal, but is also best practice for publication in any venue.
**Data citation**
Please cite (within the main reference list) any datasets stored in external repositories that are mentioned within their manuscript. For previously published datasets, we ask that you cite both the related research article(s) and the datasets themselves. For more information on how to cite datasets in submitted manuscripts, please see our data availability statements and data citations policy:<br>https://www.nature.com/documents/nr-data-availability-statements-data-citations.pdf<br><br>Citing and referencing data in publications supports reproducible research, by increasing the transparency and provenance tracking of data generated or analyzed during research. Citing data formally in reference lists also helps facilitate the tracking of data reuse and may help assign credit for individuals' contributions to research. A number of Springer Nature imprints are signatories of the Joint Declaration on Data Citation Principles, which stress the importance of data resources in scientific communication.

Thank you for depositing your dataset in a public repository. In addition to providing the link within the Data Availability statement, we ask that you also cite the dataset in the main reference list.

Citing and referencing data in publications supports reproducible research, by increasing the transparency and provenance tracking of data generated or analysed during research. Citing data formally in reference lists also helps facilitate the tracking of data reuse and may help assign credit for individuals' contributions to research. A number of Springer Nature imprints are signatories of the Joint Declaration on Data Citation Principles, which stress the importance of data resources in scientific communication.

Code availability and citation

To adhere to community standards and promote transparency in research, any custom software or code should be made publicly available, ideally before publication so that referees can test the code and comment on it.

Please include a statement under the heading "Code Availability", indicating whether and how the custom code/software reported in your study can be accessed, including any restrictions to access. This section should also include information on the versions of any software used, if relevant, and any specific variables or parameters used to generate, test, or process the current dataset. Code availability statements should be provided as a separate section after the Data Availability section.

Upon publication, Nature Portfolio journals consider it best practice to release custom computer code in a way that allows readers to repeat the published results. Code should be deposited in a DOI-minting repository such as Zenodo, Gigantum or Code Ocean and cited in the reference list following the guidelines described in our policy pages (see link below). Authors are encouraged to manage subsequent code versions and to use a license approved by the open source initiative. Full details about how the code can be accessed and any restrictions must be described in the Code Availability statement.

See here for more information about Nature Portfolio's code availability policies: https://www.nature.com/nature-portfolio/editorial-policies/reporting-standards#availability-of-computer-code

We also provide a Code and Software submission checklist that you may find useful: https://www.nature.com/documents/nr-software-policy.pdf

Please note: because of advanced features used in this form, you must use Adobe Reader to open the document and complete it.
Thank you for including a Code Availability statement in your manuscript. However, we noted that you have only indicated that custom code are available upon request. To adhere to community standards and promote transparency in research, the Code Availability Statement must indicate whether and how the code or algorithm can be accessed, including any restrictions to access. Public release of custom software may be required for publication in a Nature Portfolio journal.<br><br>Upon publication, Nature Portfolio journals consider it best practice to release custom computer code in a way that allows readers to repeat the published results. Code should be deposited in a DOI-minting repository such as Zenodo, Gigantum or Code Ocean and cited in the reference list following the guidelines described in our policy pages (see link below). Authors are encouraged to manage subsequent code versions and to use a license approved by the open source initiative. Full details about how the code can be accessed and any restrictions must be described in the Code Availability statement.<br><br>See here for more information about Nature Portfolio's code availability policies: https://www.nature.com/nature-portfolio/editorial-policies/reporting-standards#availability-of-computer-code<br><br>We also provide a Code and Software submission checklist that you may find useful: https://www.nature.com/documents/nr-software-policy.pdf<br><br>Please note: because of advanced features used in this form, you must use Adobe Reader to open the document and complete it.
Thank you for making your custom code available via Github. Upon publication, Nature Portfolio journals consider it best practice to release custom computer code in a way that allows readers to repeat the published results. Code should be deposited in a DOI-minting repository such as Zenodo, Gigantum or Code Ocean and cited in the reference list following the guidelines described in our policy pages (see link below). Authors are encouraged to manage subsequent code versions and to use a license approved by the open source initiative.<br><br>See here for more information about our code availability policies: https://www.nature.com/nature-portfolio/editorial-policies/reporting-standards#availability-o

f-computer-code
**Ethics**
We believe that authors, peer reviewers and editors should be required to disclose any competing interests that might influence their decisions and conclusions around a particular piece of content. In the interests of transparency and to help readers form their own judgements of potential bias, Nature Portfolio journals require authors to declare any competing financial and/or non-financial interests in relation to the work described.<br><br>Please provide a 'Competing interests' statement using one of the following standard sentences:<br><br>1. The authors declare the following competing interests: [specify competing interests]<br>2. The authors declare no competing interests.<br><br>See the Nature Portfolio competing interests policy for further information: https://www.nature.com/nature-research/editorial-policies/competing-interests<br>The Springer Nature policy can be found here: https://www.springernature.com/gp/policies/editorial-policies
We believe that research that involves the use of clinical, biomedical or biometric data from human participants must only be carried out with the explicit consent of those whose data are involved. Consent must be obtained without any form of coercion and with participants' explicit understanding of the purpose for which their data will be used.<br><br>Because your study includes human participants, confirmation that all relevant ethical regulations were followed is needed for publication in any Springer Nature journal, and that informed consent was obtained. This must be stated in the Methods section, including the name of the board and institution that approved the study protocol.<br><br>Further details about the Nature Portfolio policy can be found at https://www.nature.com/commsbio/editorial-policies/ethics-and-biosecurity
**Reporting & reproducibility**

We believe that research publications should adhere to high standards of transparency and robustness in their methods and results. This, in turn, supports the principle of reproducibility, which is a foundation of good research, especially in the natural sciences. All data that support the conclusions drawn must be presented in the manuscript unless they are published elsewhere.<br><br>Nature Portfolio journals do not allow statements of “data not shown”. Please remove these statements or provide the relevant data.
We believe that research publications should adhere to high standards of transparency and robustness in their methods and results. This, in turn, supports the principle of reproducibility, which is a foundation of good research, especially in the natural sciences.<br><br>The Methods section should contain sufficient detail such that the work could be repeated. It is preferable that all key methods be included in the main manuscript, rather than in the Supplementary Information. Please avoid use of “as described previously” or similar, and instead detail the specific methods used, with appropriate attribution.<br><br>Please note that Nature Portfolio journals allow unlimited space for Methods.
We encourage you to share your step-by-step experimental protocols on a protocol sharing platform of their choice. The Nature Portfolio’s Protocol Exchange is a free-to-use and open resource for protocols; protocols deposited in Protocol Exchange are citable and can be linked from the published article. More details can be found at www.nature.com/protocolexchange/about
**Materials availability**
We encourage you to include within the Methods and/or Data Availability Statement details regarding materials availability for the reported components.
**Statistical reporting**

Wherever statistics have been derived (e.g. error bars, box plots, statistical significance) figure legends should provide and define the n number (i.e. the sample size used to derive statistics) as a precise value (not a range), using the wording “n=X biologically independent samples/animals/cells/independent experiments/n= X cells examined over Y independent experiments” etc. as applicable. The figure legends must also indicate the statistical test used. Where appropriate, please indicate in the figure legends whether the statistical tests were one-sided or two-sided and whether adjustments were made for multiple comparisons. For null hypothesis testing, please indicate the test statistic (e.g. F, t, r) with confidence intervals, effect sizes, degrees of freedom and P values noted.

All error bars need to be defined in the figure legends (e.g. SD, SEM) together with a measure of centre (e.g. mean, median). For example, the legends should state something along the lines of “Data are presented as mean values +/- SEM” as appropriate. All box plots need to be defined in the legends in terms of minima, maxima, centre, bounds of box and whiskers and percentile.

For examples of expected description of statistics in figure legends, please see the following: https://www.nature.com/articles/s41467-019-11636-5 or https://www.nature.com/articles/s41467-019-11510-4.

When describing results as "significant" in the main text, please include details about the statistical test used and provide an exact p-value, rather than a signifcance threshold.

Please note that statistics such as error bars significance and p values cannot be derived from n<3 and must be removed in all such cases.

We strongly discourage deriving statistics from technical replicates, unless there is a clear scientific justification for why providing this information is important. Conflating technical and biological variability, e.g., by pooling technically replicates samples across independent experiments is strongly discouraged.

For examples of expected description of statistics in figure legends, please see the following: https://www.nature.com/articles/s41467-019-11636-5 or https://www.nature.com/articles/s41467-019-11510-4.

To improve reproducibility of your analyses, please provide details regarding your treatment of outliers.

To improve reproducibility of your analyses, please detail the methods used for data fitting and provide a rationale for this approach.
Bar graphs should only be used to present counts or proportions. If you are using bar graphs that present means/averages, it is best practice to include individual data points and/or convert the graph to a boxplot or dot-plot. You may wish to refer to this blog post (https://ecrlife420999811.wordpress.com/2018/07/10/beyond-bar-graphs-free-tools-and-resources-for-creating-more-transparent-figures-for-small-datasets/) about representing data distribution in plots (particularly for small datasets).
When publishing identifiable images from human research participants, authors must include a statement in the published paper affirming that they have obtained informed consent for publication of the images. All reasonable measures must be taken to protect patient anonymity. Black bars over the eyes are not acceptable means of anonymization. In certain cases, we may insist upon obtaining evidence of informed consent from authors. Images without appropriate consent will be removed from publication. Please confirm that your images are in compliance with these policies. Further details can be found here: https://www.nature.com/commsbio/editorial-policies/ethics-and-biosecurity#ethics-policy
