# Supplementary figures and images for: A wearable platform for closed-loop stimulation and recording of single-neuron and local field potential activity in freely moving humans
Source: Nat Neurosci. 2023 Feb 20;26(3):517–27. doi: 10.1038/s41593-023-01260-4 (PMC9991917; doi:10.1038/s41593-023-01260-4)

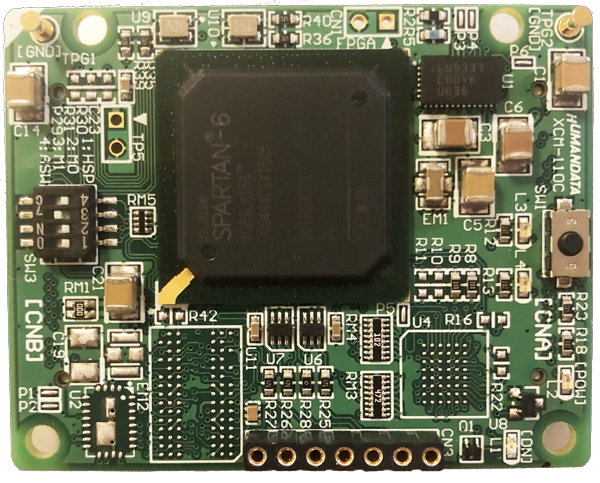

Supplement: Source Data Fig. 1 — Contains data (Figure1_source_data.xlsx) and images (Figure1_source_images) used in making Fig. 1. [file 41593_2023_1260_MOESM4_ESM.zip › Figure1_source_images/Fig1B_comm.tif]

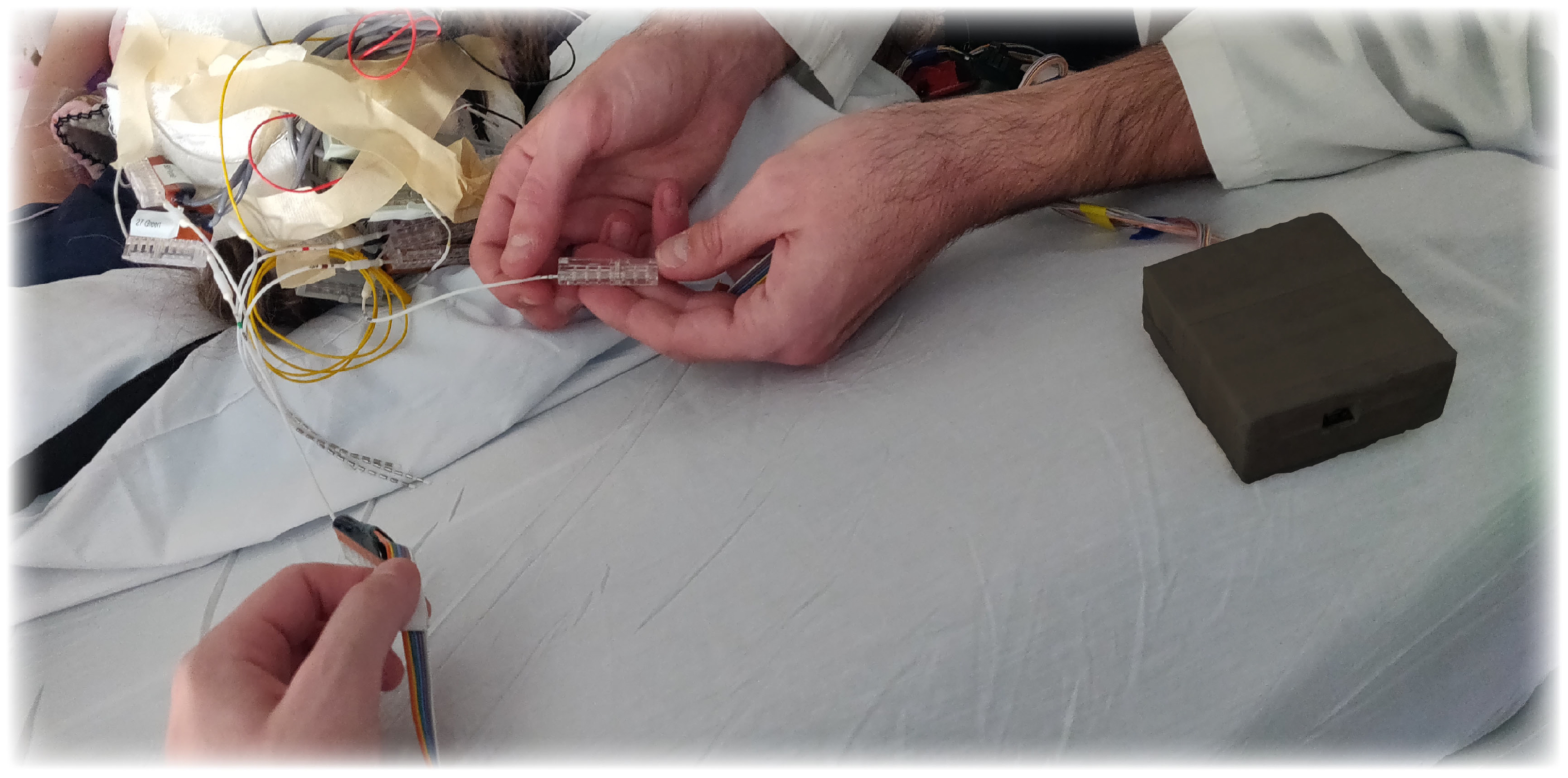

Supplement: Source Data Fig. 1 — Contains data (Figure1_source_data.xlsx) and images (Figure1_source_images) used in making Fig. 1. [file 41593_2023_1260_MOESM4_ESM.zip › Figure1_source_images/Fig1C_connections.jpg]

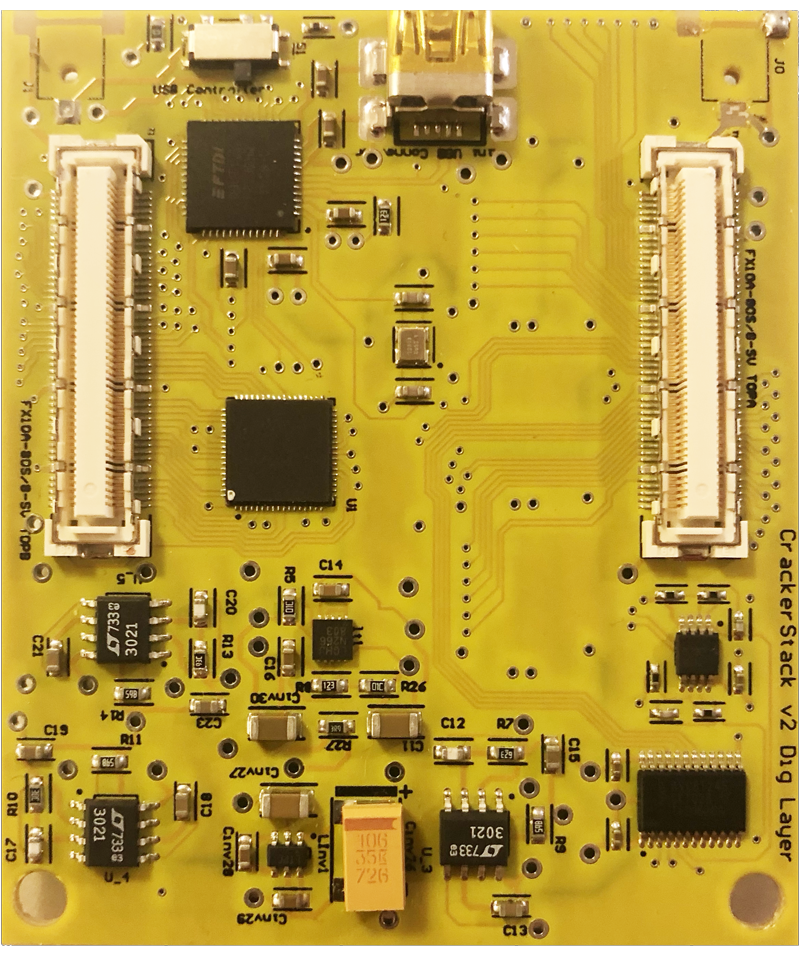

Supplement: Source Data Fig. 1 — Contains data (Figure1_source_data.xlsx) and images (Figure1_source_images) used in making Fig. 1. [file 41593_2023_1260_MOESM4_ESM.zip › Figure1_source_images/Fig1B_digital.tif]

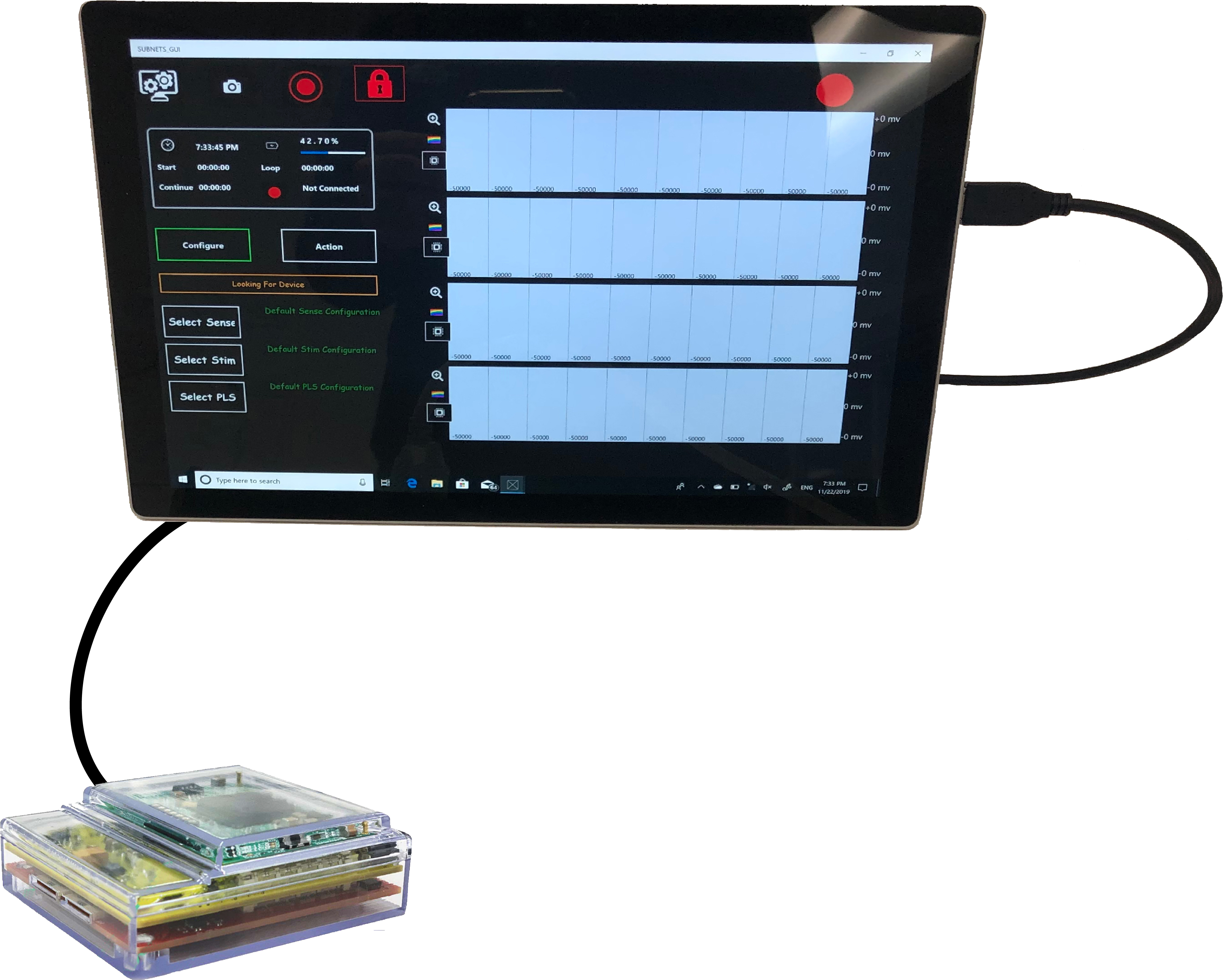

Supplement: Source Data Fig. 1 — Contains data (Figure1_source_data.xlsx) and images (Figure1_source_images) used in making Fig. 1. [file 41593_2023_1260_MOESM4_ESM.zip › Figure1_source_images/Fig1A_tablet.jpg]

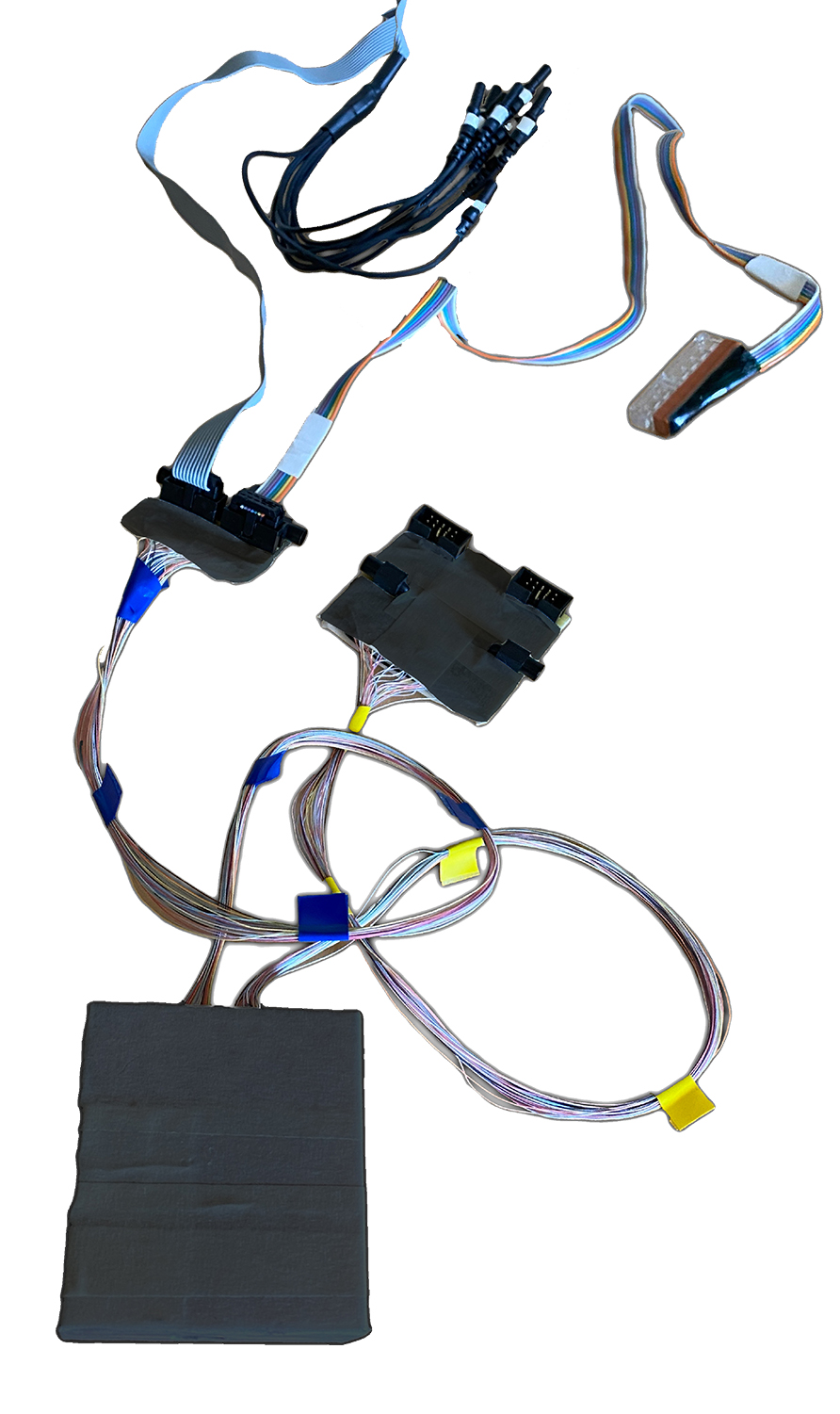

Supplement: Source Data Fig. 1 — Contains data (Figure1_source_data.xlsx) and images (Figure1_source_images) used in making Fig. 1. [file 41593_2023_1260_MOESM4_ESM.zip › Figure1_source_images/Fig1D_connections.jpg]

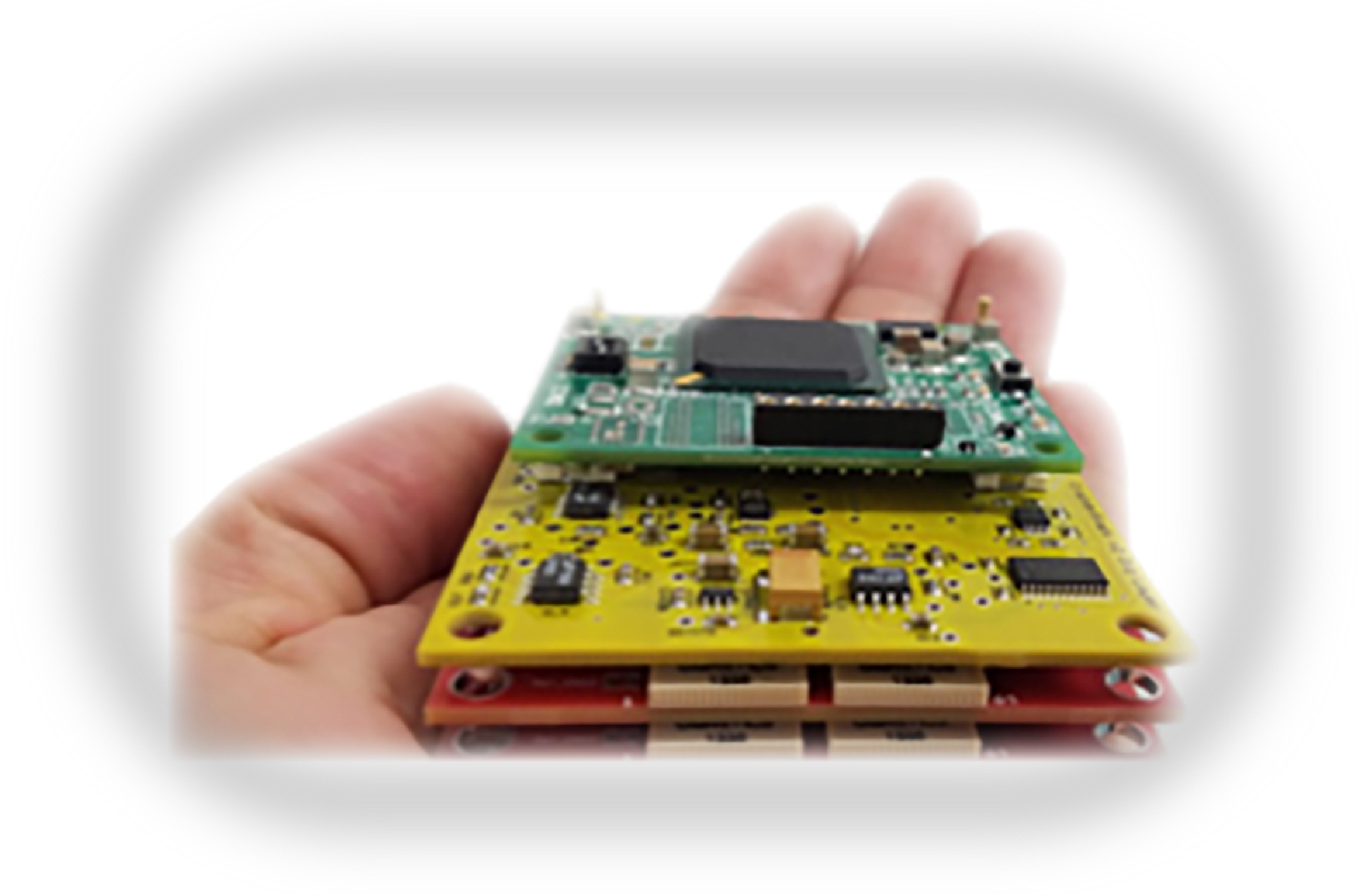

Supplement: Source Data Fig. 1 — Contains data (Figure1_source_data.xlsx) and images (Figure1_source_images) used in making Fig. 1. [file 41593_2023_1260_MOESM4_ESM.zip › Figure1_source_images/Fig1A_handheld.jpg]

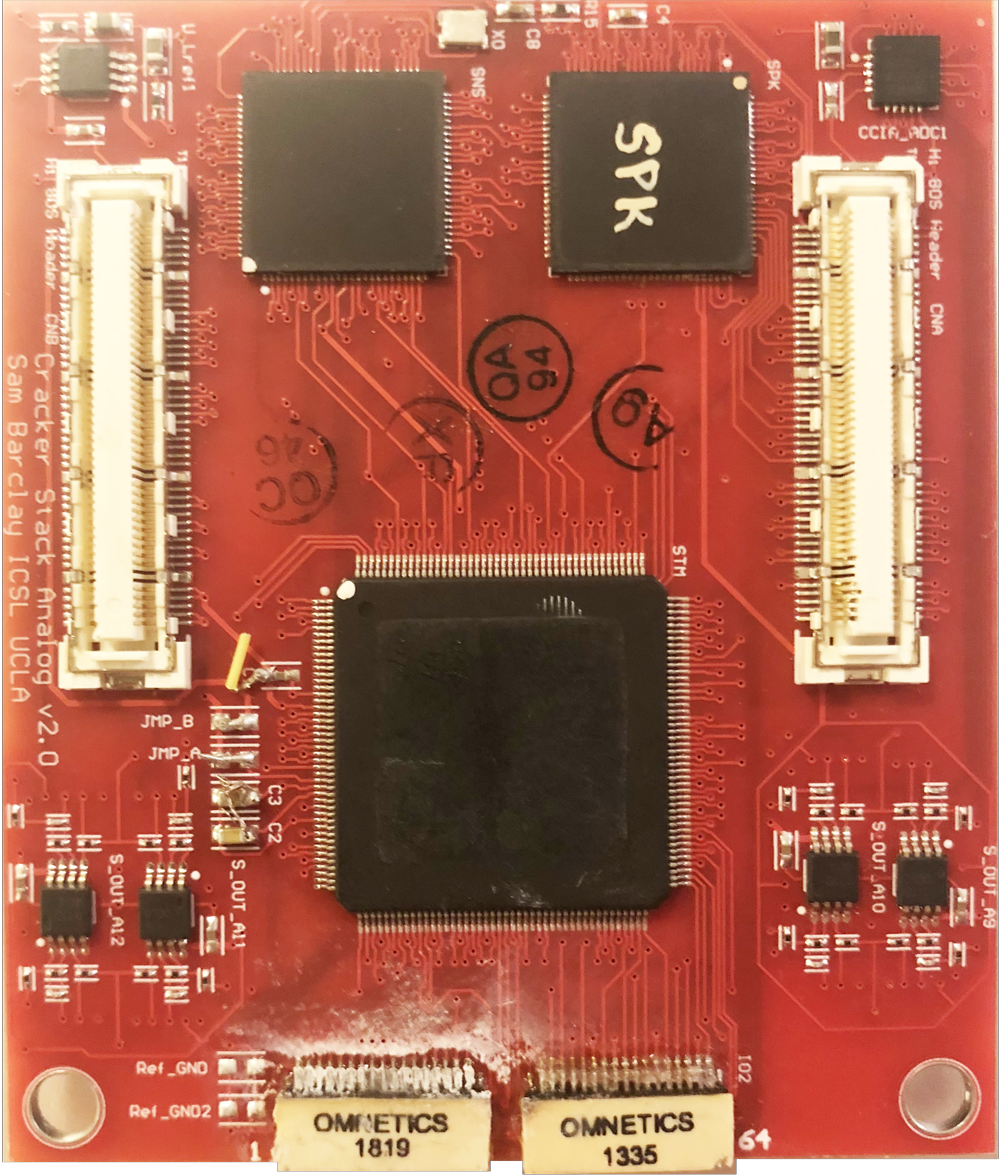

Supplement: Source Data Fig. 1 — Contains data (Figure1_source_data.xlsx) and images (Figure1_source_images) used in making Fig. 1. [file 41593_2023_1260_MOESM4_ESM.zip › Figure1_source_images/Fig1B_analog.tif]

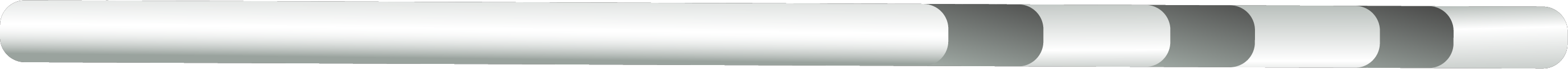

Supplement: Source Data Fig. 1 — Contains data (Figure1_source_data.xlsx) and images (Figure1_source_images) used in making Fig. 1. [file 41593_2023_1260_MOESM4_ESM.zip › Figure1_source_images/Fig1B_electrode.tif]

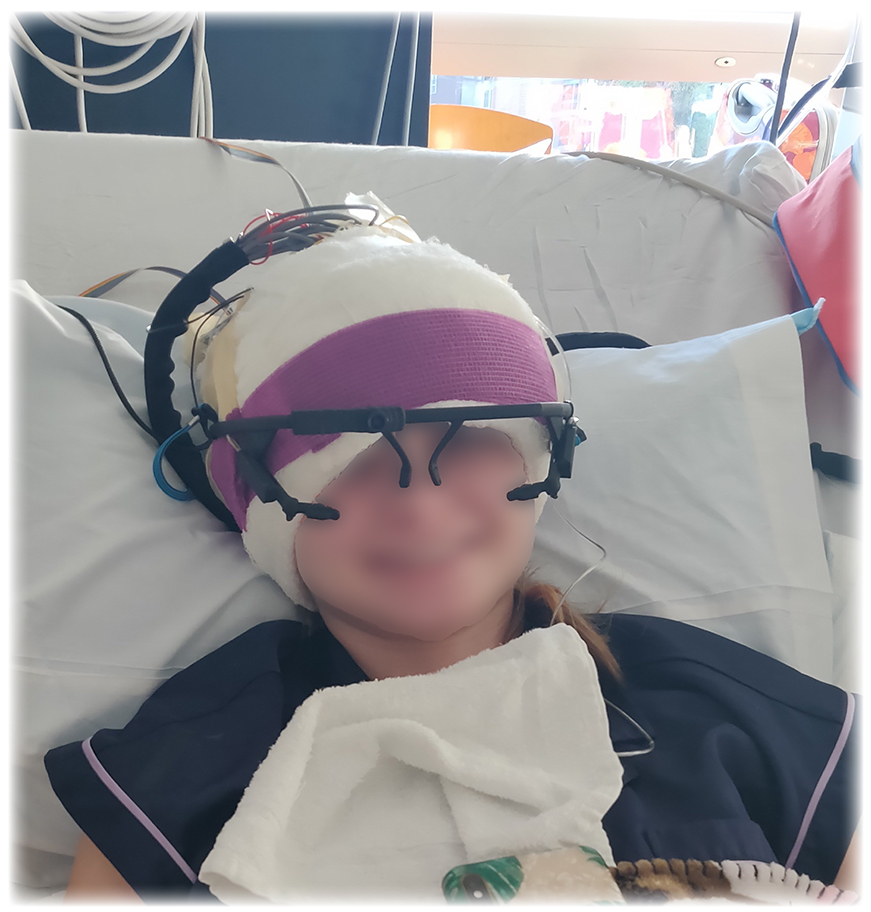

Supplement: Source Data Fig. 1 — Contains data (Figure1_source_data.xlsx) and images (Figure1_source_images) used in making Fig. 1. [file 41593_2023_1260_MOESM4_ESM.zip › Figure1_source_images/Fig1C_participant.jpg]

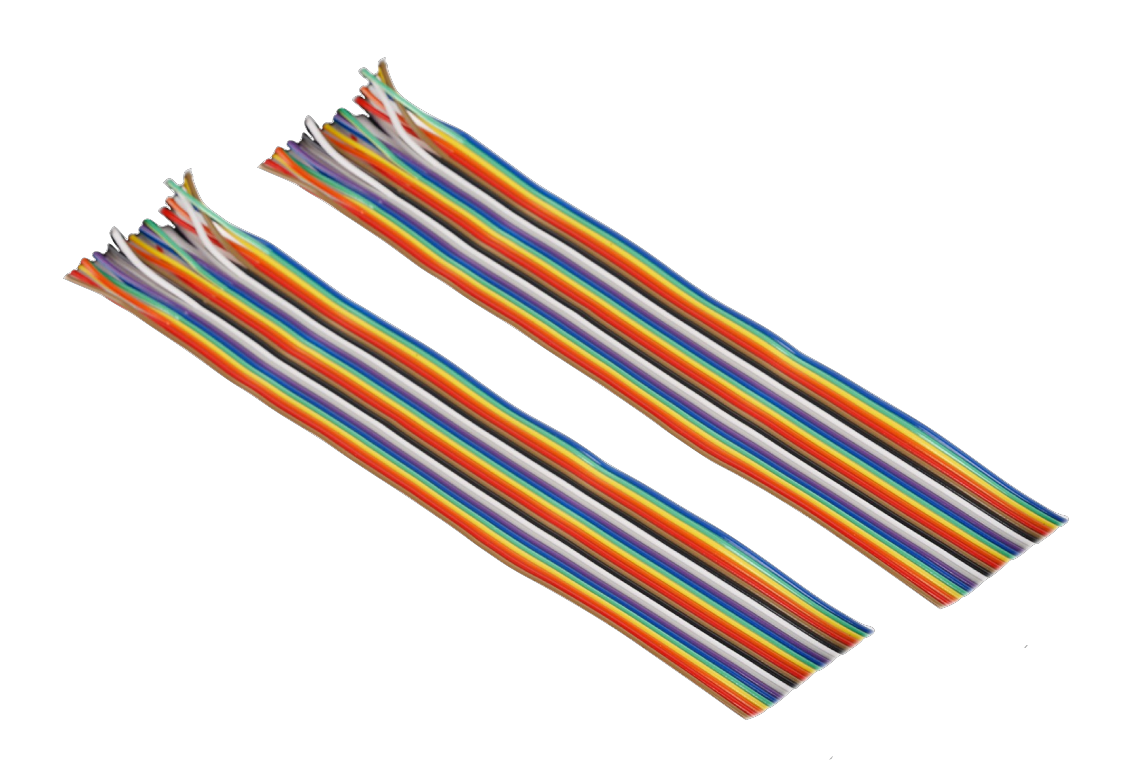

Supplement: Source Data Fig. 1 — Contains data (Figure1_source_data.xlsx) and images (Figure1_source_images) used in making Fig. 1. [file 41593_2023_1260_MOESM4_ESM.zip › Figure1_source_images/Fig1B_ribbon.tif]

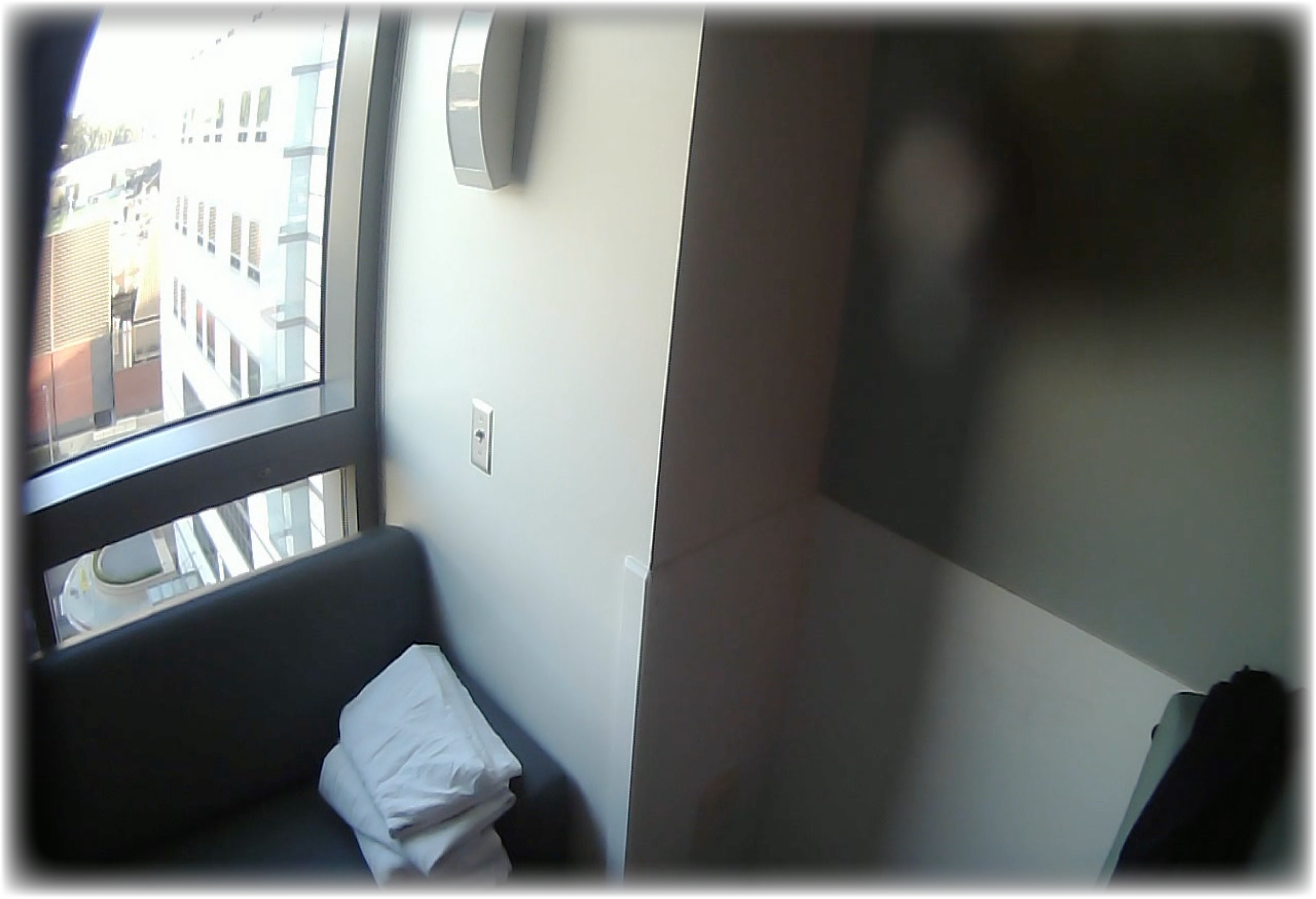

Supplement: Source Data Fig. 2 — Contains data (Figure2_source_data.xlsx) and images (Figure2_source_images) used in making Fig. 2. [file 41593_2023_1260_MOESM5_ESM.zip › Figure2_source_images/Fig2B_worldview.tif]

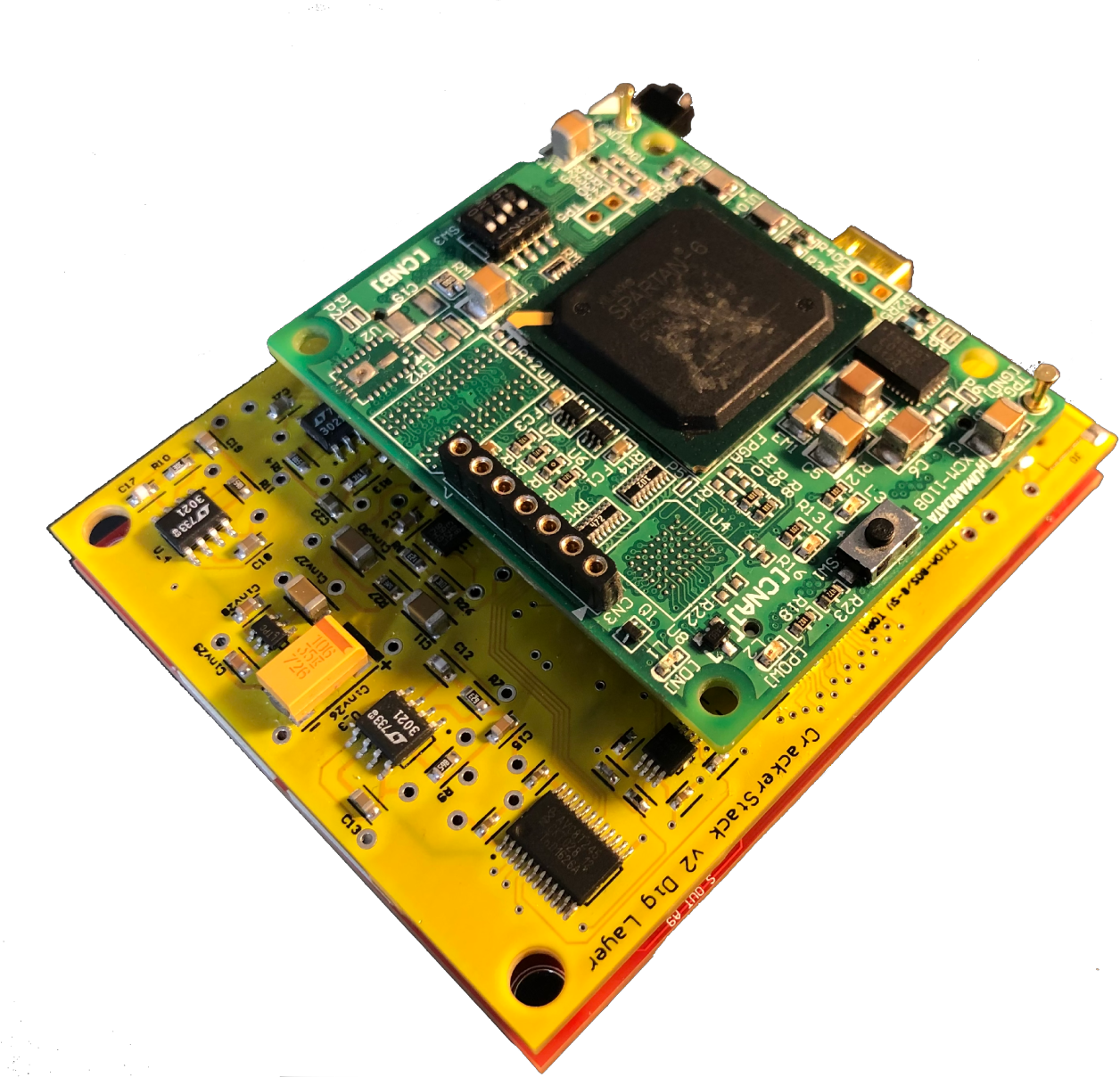

Supplement: Source Data Fig. 2 — Contains data (Figure2_source_data.xlsx) and images (Figure2_source_images) used in making Fig. 2. [file 41593_2023_1260_MOESM5_ESM.zip › Figure2_source_images/Fig2A_neurostack.tif]

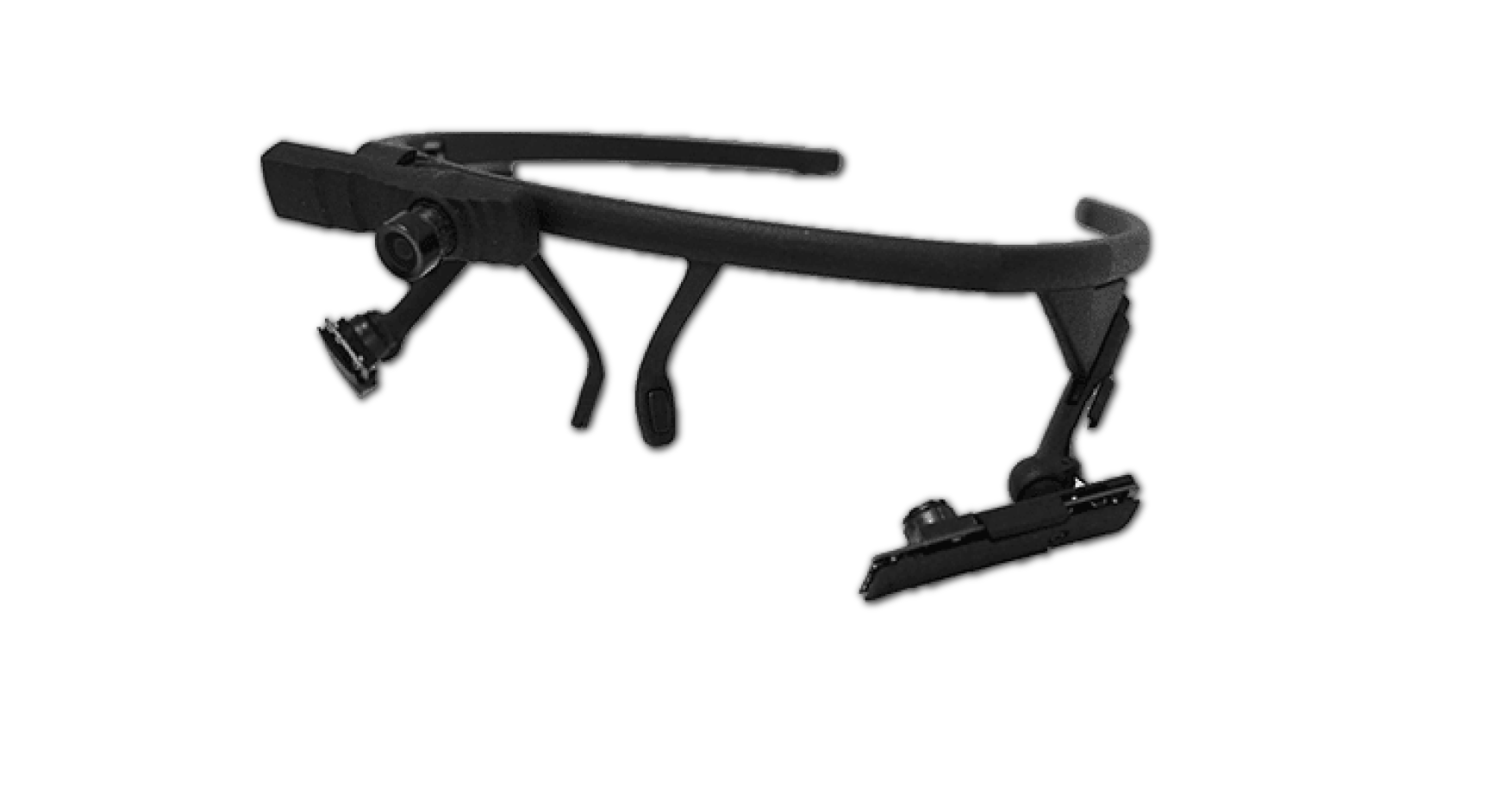

Supplement: Source Data Fig. 2 — Contains data (Figure2_source_data.xlsx) and images (Figure2_source_images) used in making Fig. 2. [file 41593_2023_1260_MOESM5_ESM.zip › Figure2_source_images/Fig2A_pupils.tif]

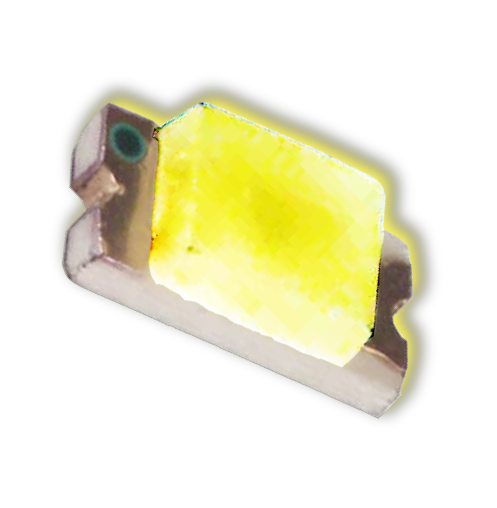

Supplement: Source Data Fig. 2 — Contains data (Figure2_source_data.xlsx) and images (Figure2_source_images) used in making Fig. 2. [file 41593_2023_1260_MOESM5_ESM.zip › Figure2_source_images/Fig2A_led.tif]

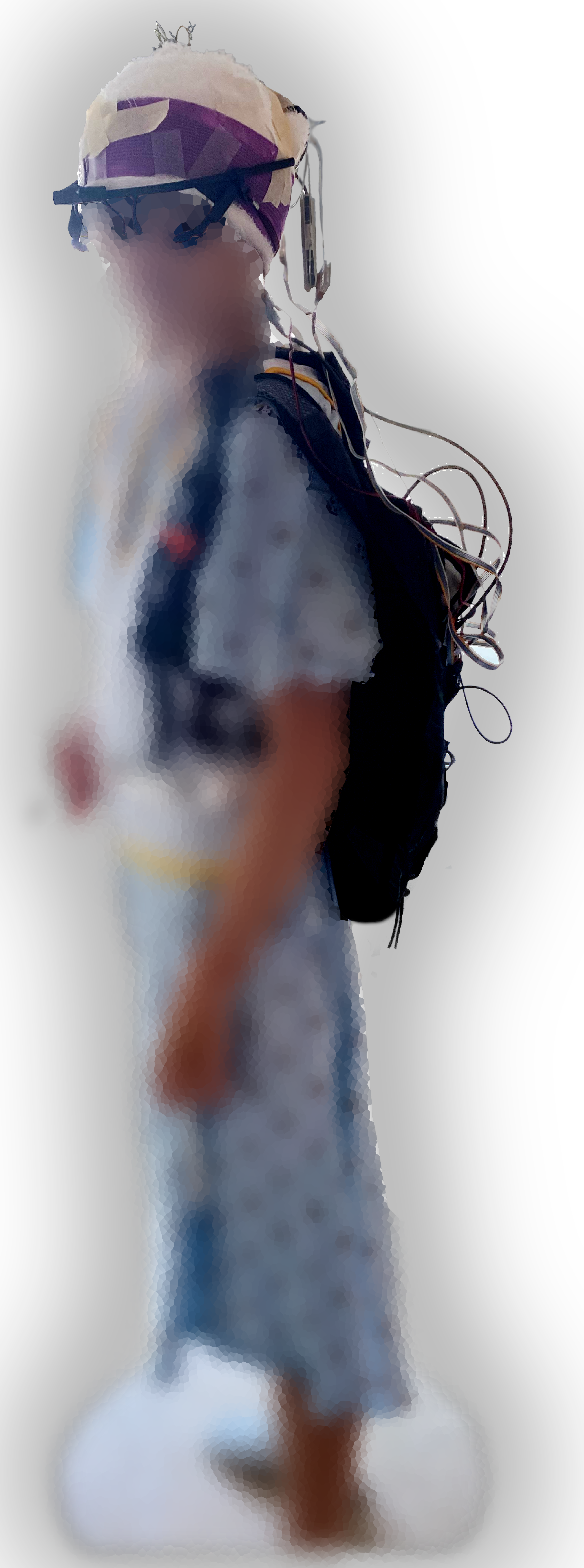

Supplement: Source Data Fig. 2 — Contains data (Figure2_source_data.xlsx) and images (Figure2_source_images) used in making Fig. 2. [file 41593_2023_1260_MOESM5_ESM.zip › Figure2_source_images/Fig2A_participant.tif]

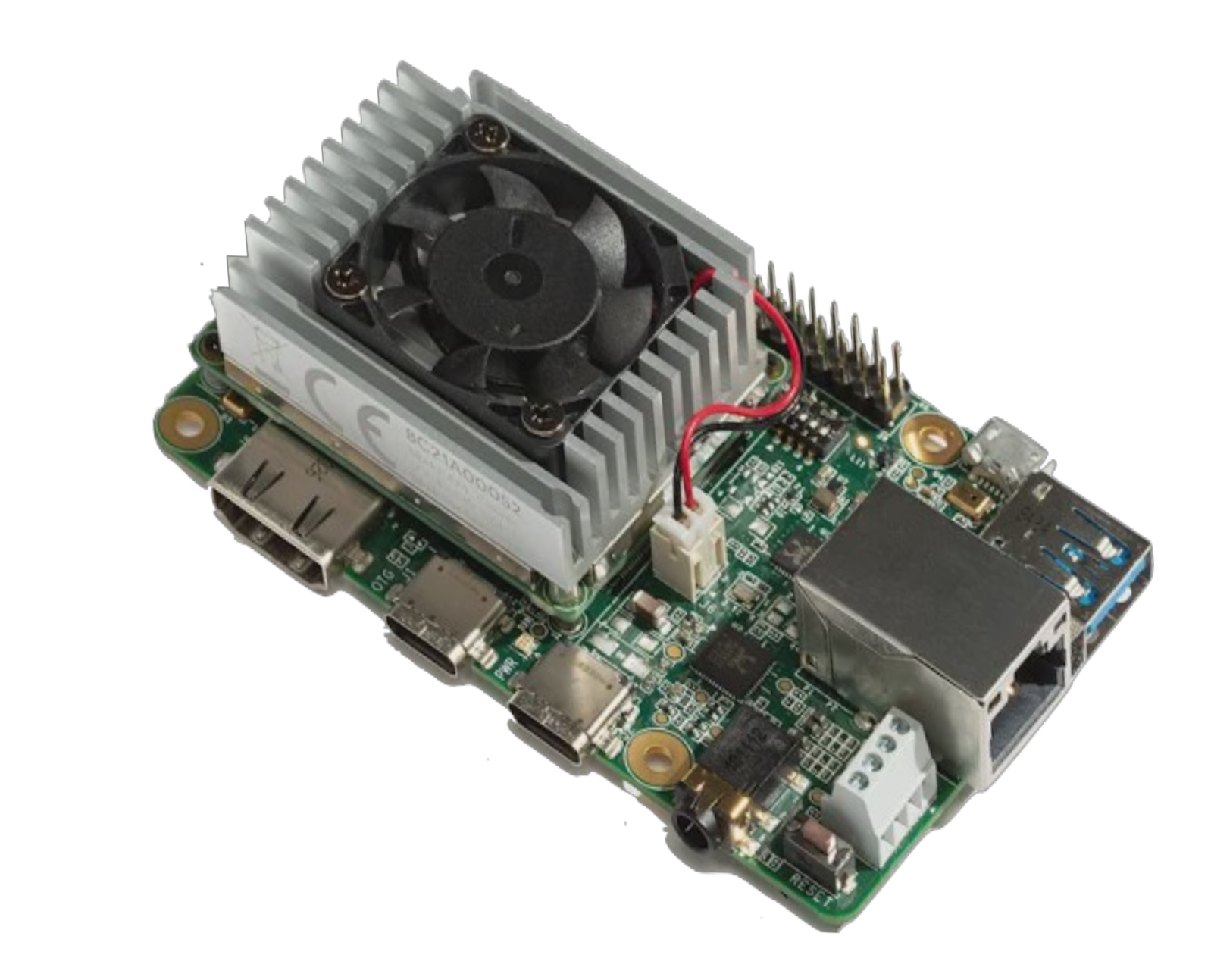

Supplement: Source Data Fig. 2 — Contains data (Figure2_source_data.xlsx) and images (Figure2_source_images) used in making Fig. 2. [file 41593_2023_1260_MOESM5_ESM.zip › Figure2_source_images/Fig2A_coral.tif]

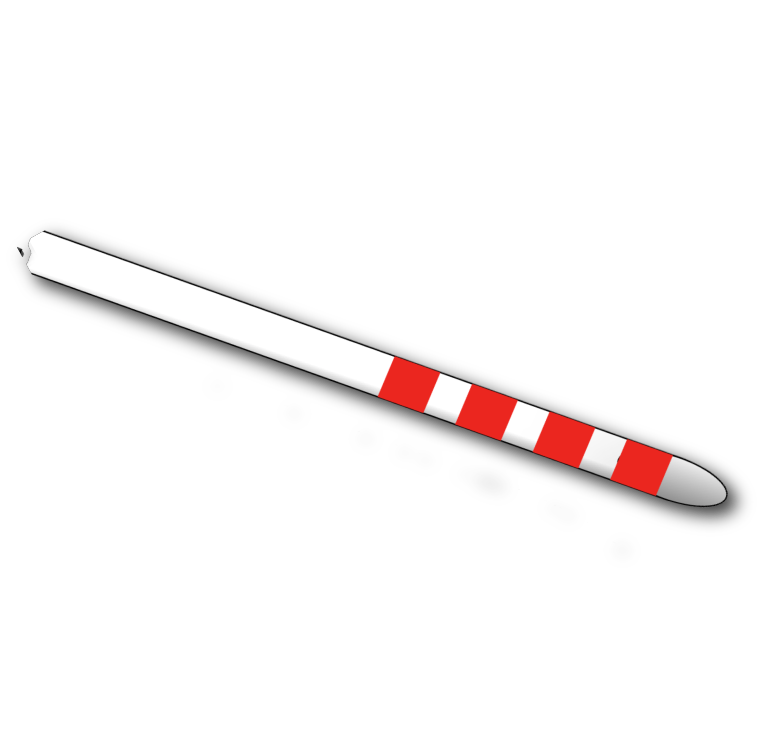

Supplement: Source Data Fig. 4 — Contains data (Figure4_source_data.xlsx) and images (Figure4_source_images) used in making Fig. 4. [file 41593_2023_1260_MOESM7_ESM.zip › Figure4_source_images/Fig4B_electrode_1.tif]

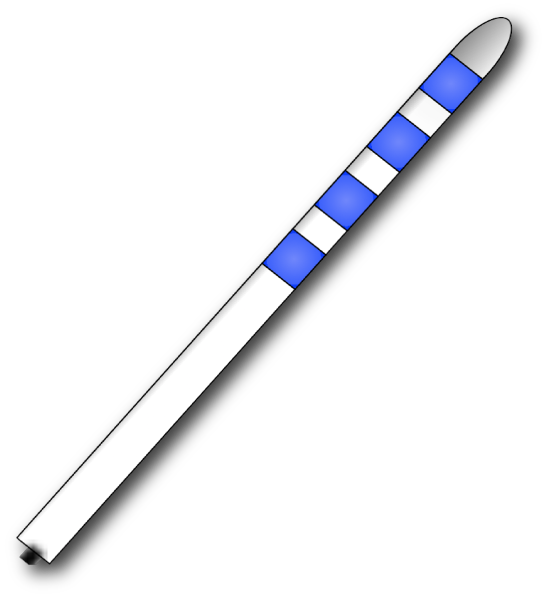

Supplement: Source Data Fig. 4 — Contains data (Figure4_source_data.xlsx) and images (Figure4_source_images) used in making Fig. 4. [file 41593_2023_1260_MOESM7_ESM.zip › Figure4_source_images/Fig4B_electrode_2.tif]

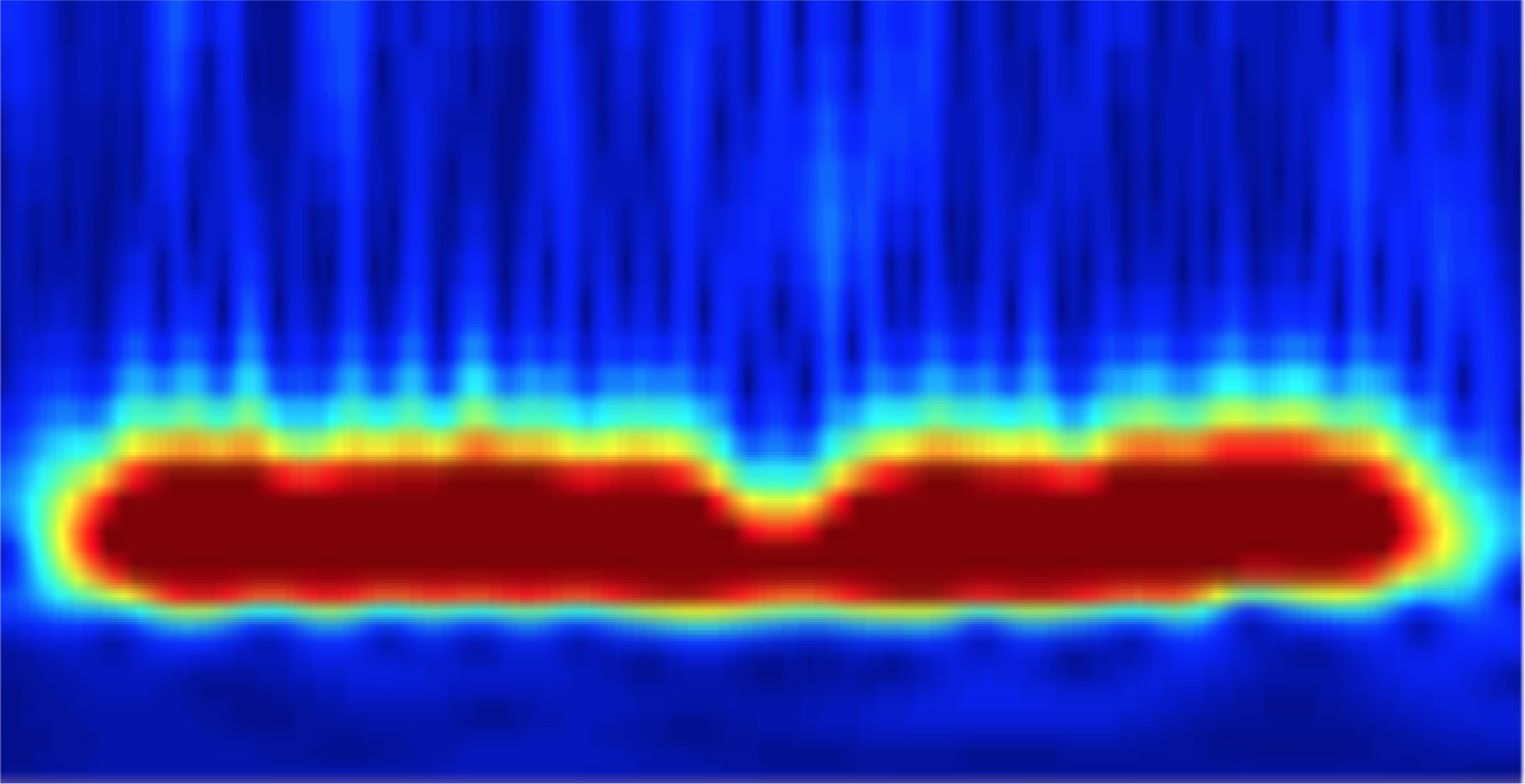

Supplement: Source Data Fig. 4 — Contains data (Figure4_source_data.xlsx) and images (Figure4_source_images) used in making Fig. 4. [file 41593_2023_1260_MOESM7_ESM.zip › Figure4_source_images/Fig4F_tf.jpg]

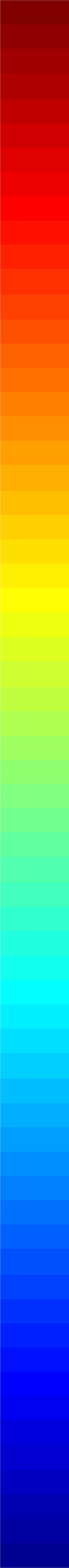

Supplement: Source Data Fig. 4 — Contains data (Figure4_source_data.xlsx) and images (Figure4_source_images) used in making Fig. 4. [file 41593_2023_1260_MOESM7_ESM.zip › Figure4_source_images/Fig4FG_colorbar.jpg]

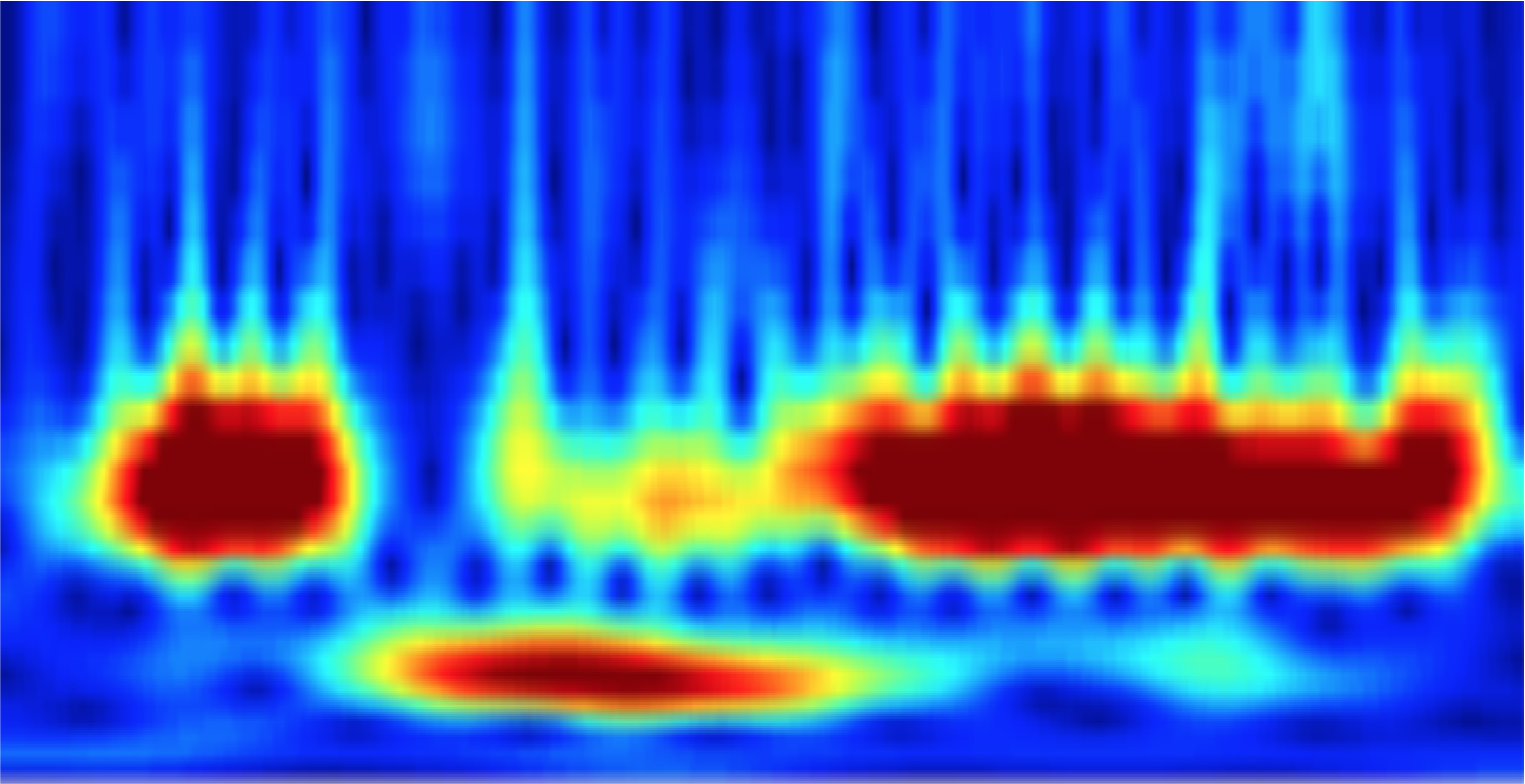

Supplement: Source Data Fig. 4 — Contains data (Figure4_source_data.xlsx) and images (Figure4_source_images) used in making Fig. 4. [file 41593_2023_1260_MOESM7_ESM.zip › Figure4_source_images/Fig4G_tf.jpg]

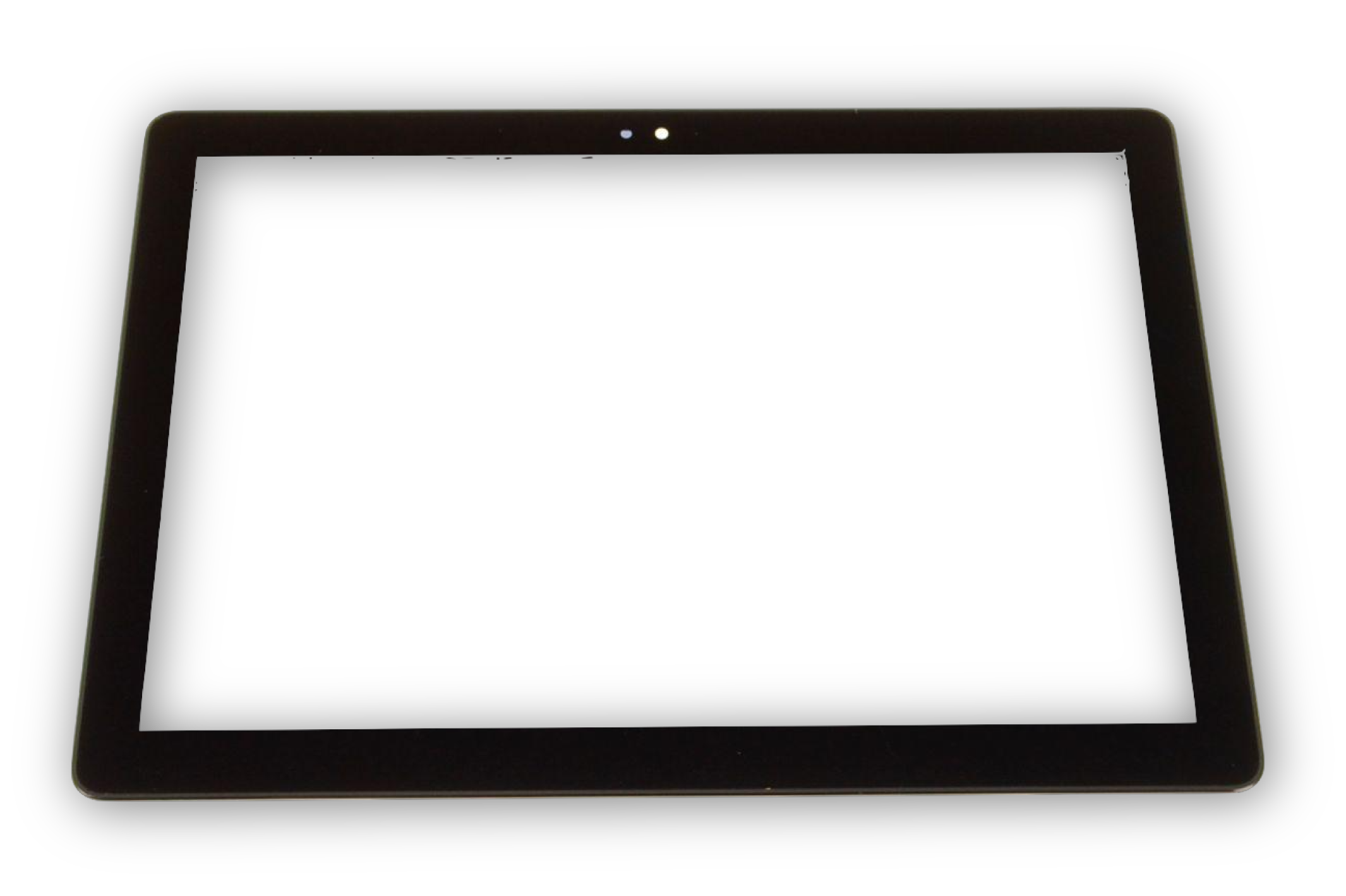

Supplement: Source Data Fig. 4 — Contains data (Figure4_source_data.xlsx) and images (Figure4_source_images) used in making Fig. 4. [file 41593_2023_1260_MOESM7_ESM.zip › Figure4_source_images/Fig4B_tablet.tif]

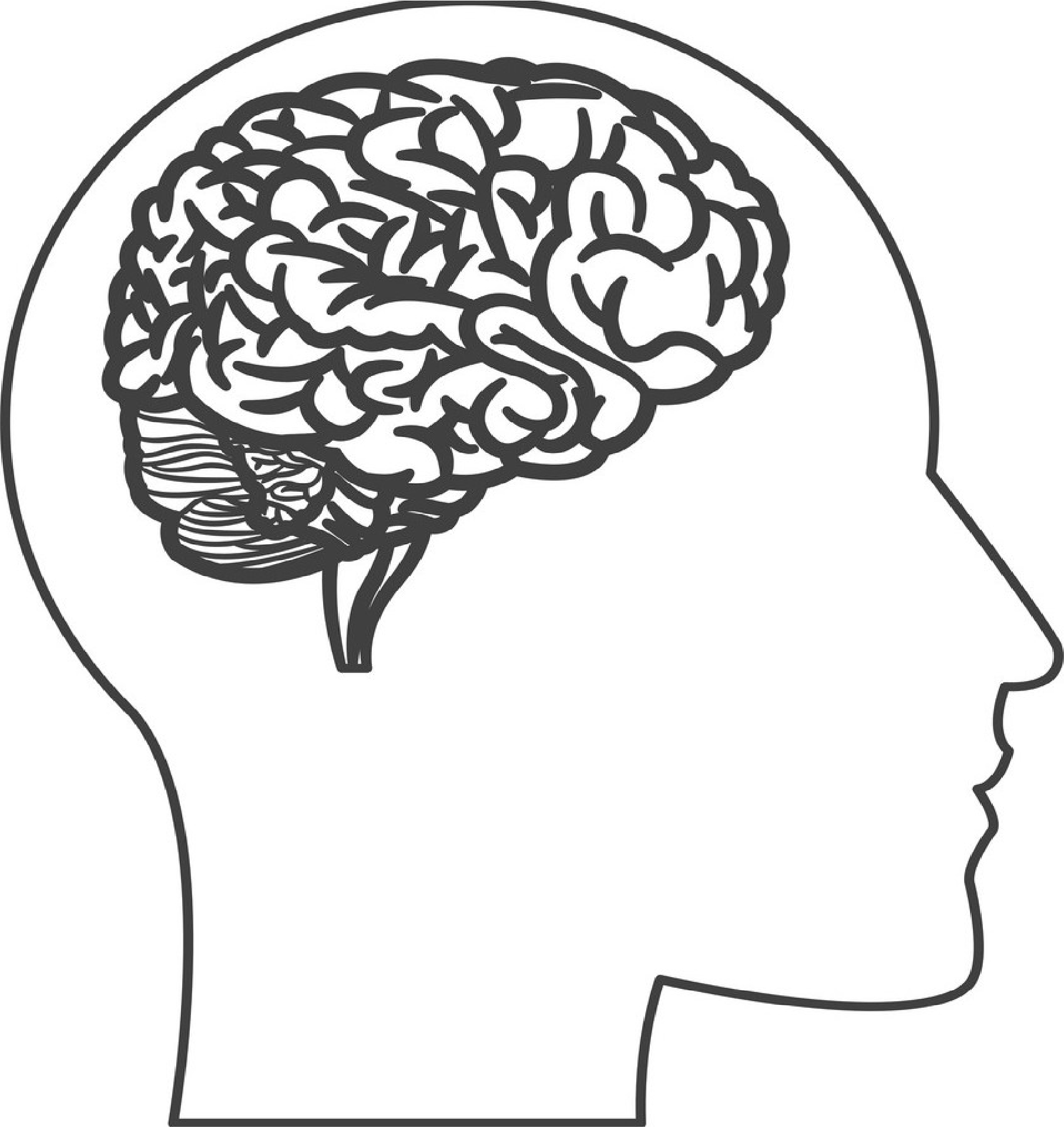

Supplement: Source Data Fig. 4 — Contains data (Figure4_source_data.xlsx) and images (Figure4_source_images) used in making Fig. 4. [file 41593_2023_1260_MOESM7_ESM.zip › Figure4_source_images/Fig4B_participant.jpg]

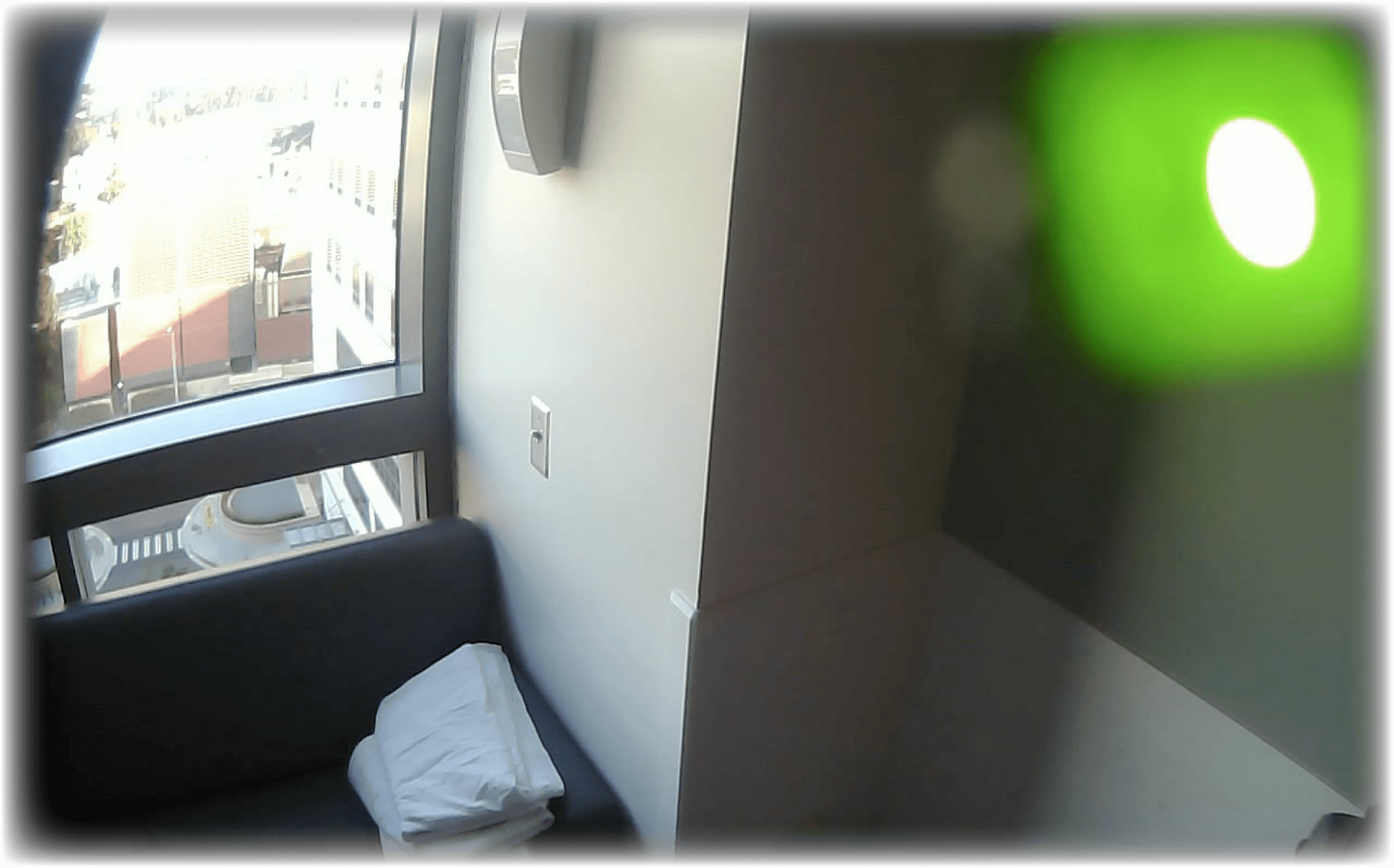

Supplement: Source Data Extended Data Fig. 2 — Contains data (EDFigure2_source_data.xlsx) and images (EDFigure2_source_images) used in making Extended Data Fig. 2. [file 41593_2023_1260_MOESM8_ESM.zip › EDFigure2_source_images/EDFigD_worldview.tif]

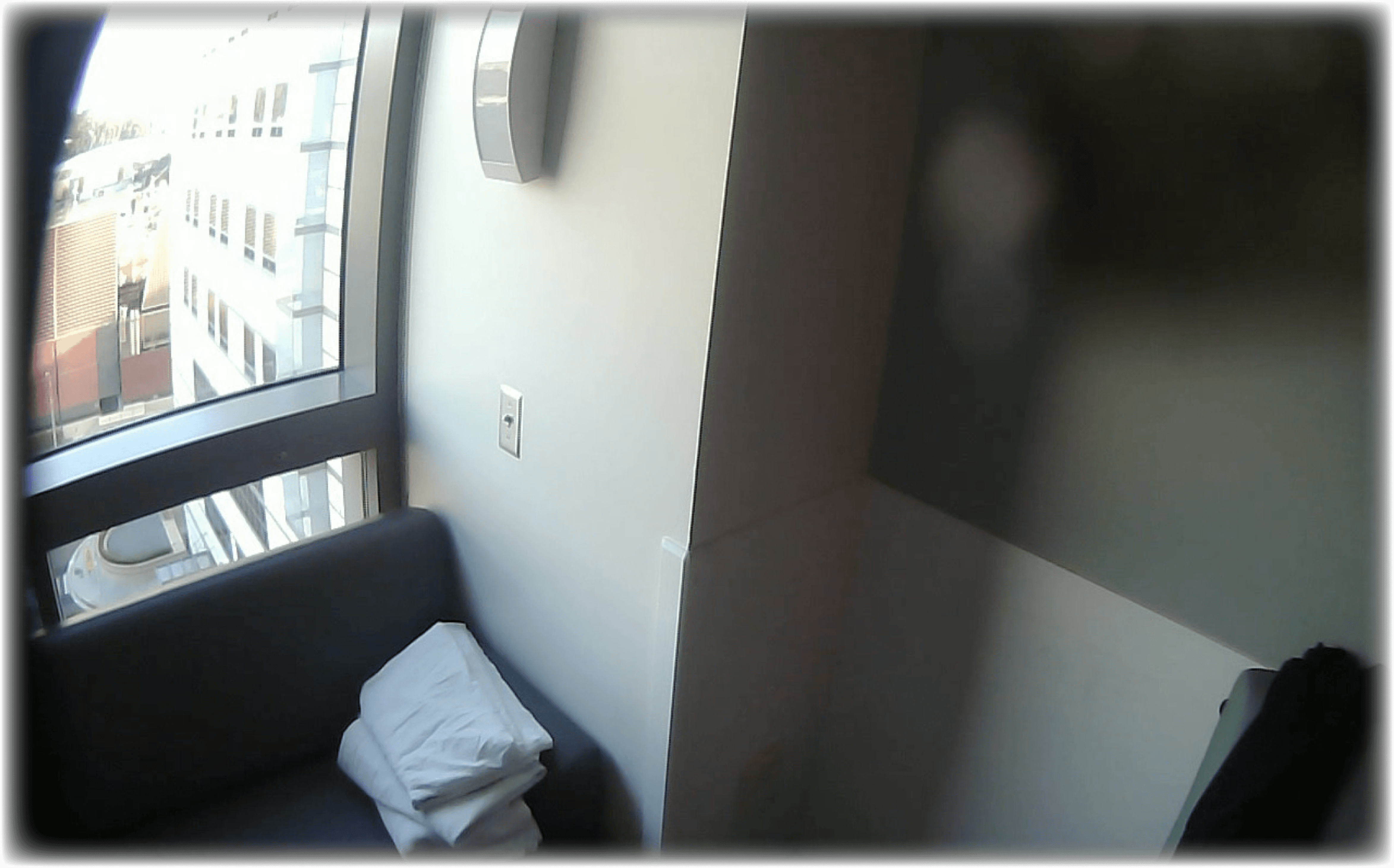

Supplement: Source Data Extended Data Fig. 2 — Contains data (EDFigure2_source_data.xlsx) and images (EDFigure2_source_images) used in making Extended Data Fig. 2. [file 41593_2023_1260_MOESM8_ESM.zip › EDFigure2_source_images/EDFigC_worldview.tif]

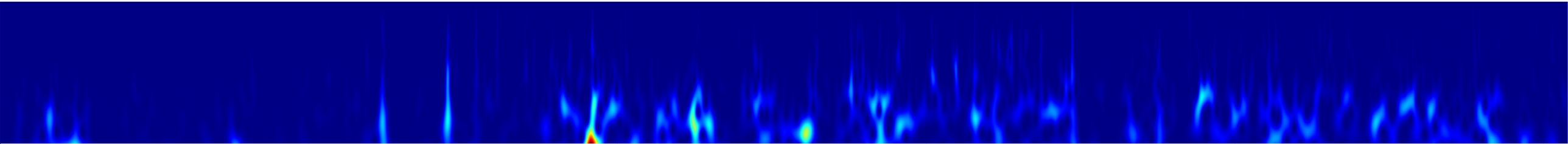

Supplement: Source Data Extended Data Fig. 3 — Contains data (EDFigure3_source_data.xlsx) and images (EDFigure3_source_images) used in making Extended Data Fig. 3. [file 41593_2023_1260_MOESM9_ESM.zip › EDFigure3_source_images/EDFig3B_tf.jpg]

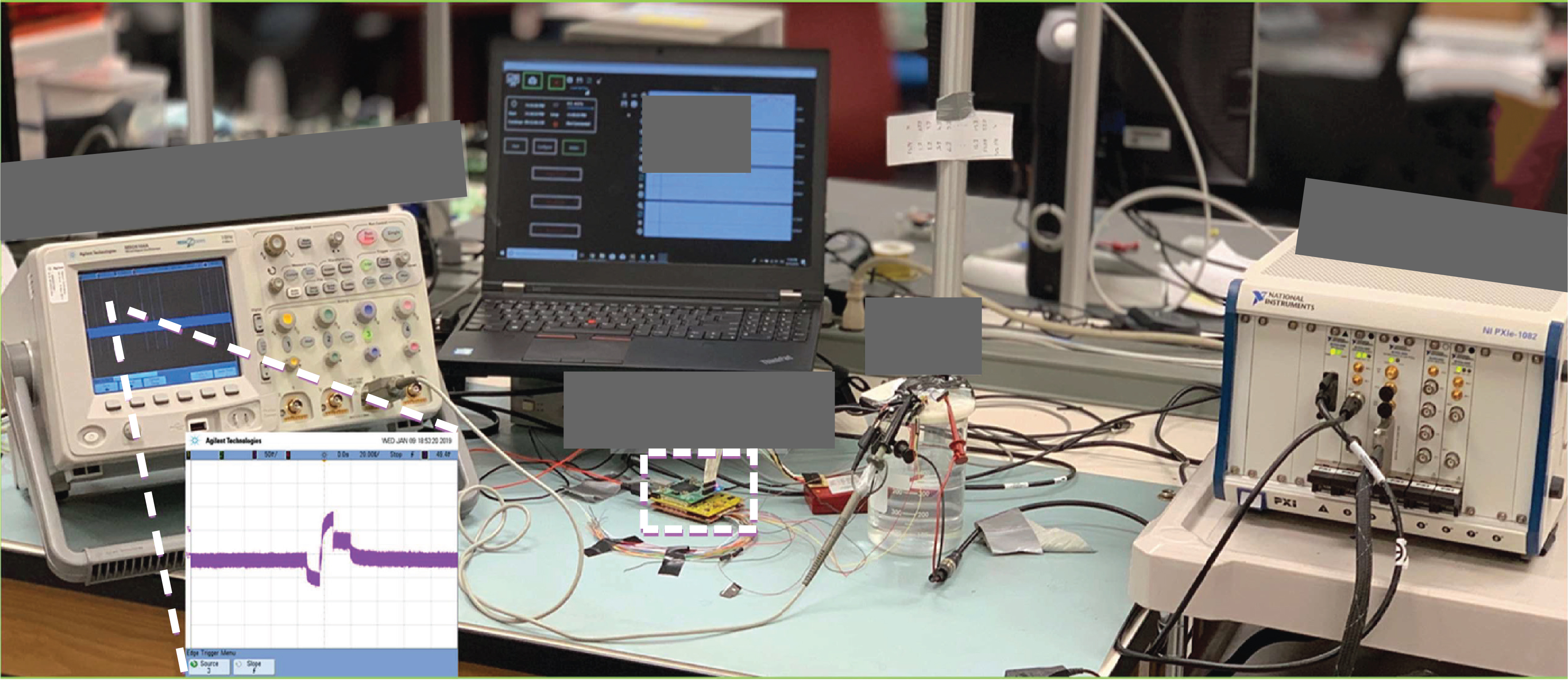

Supplement: Source Data Extended Data Fig. 5 — Contains data (EDFigure6_source_data.xlsx) and images (EDFigure6_source_images) used in making Extended Data Fig. 6. [file 41593_2023_1260_MOESM11_ESM.zip › EDFigure5_source_images/EDFig5_setup.jpg]

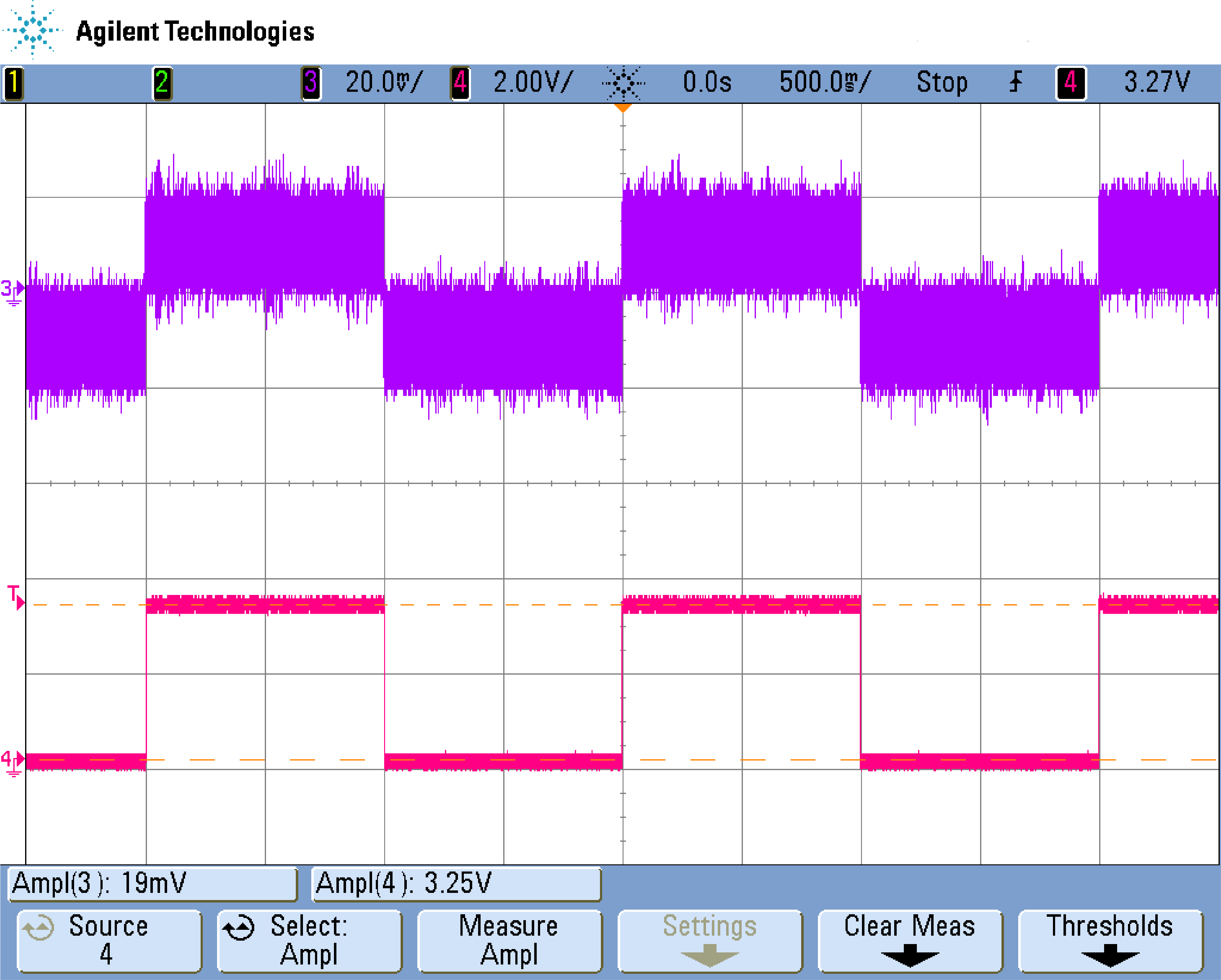

Supplement: Source Data Extended Data Fig. 6 — Contains data (EDFigure6_source_data.xlsx) and images (EDFigure6_source_images) used in making Extended Data Fig. 6. [file 41593_2023_1260_MOESM12_ESM.zip › EDFigure6_source_images/EDFig6E_oscilloscope1.jpg]

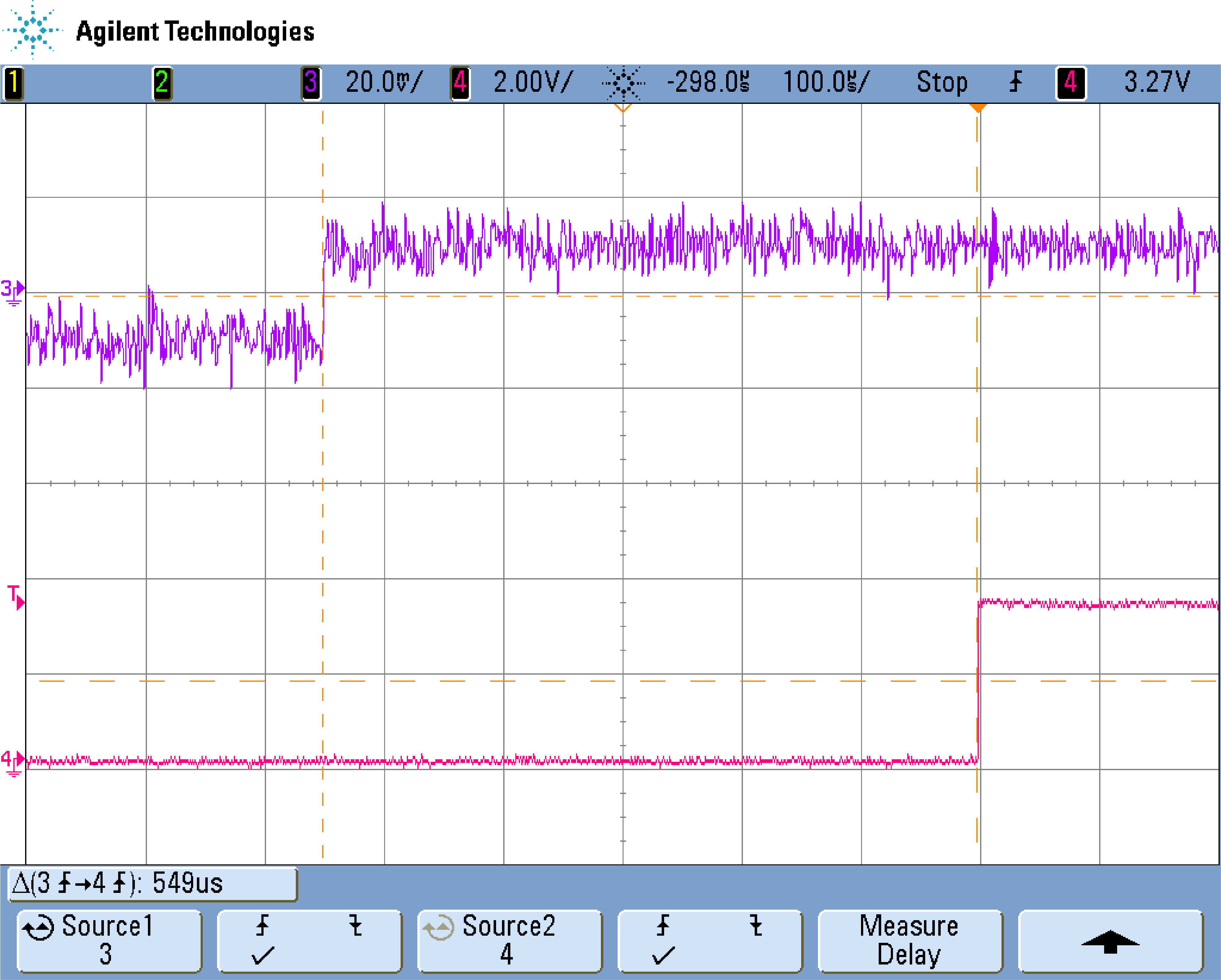

Supplement: Source Data Extended Data Fig. 6 — Contains data (EDFigure6_source_data.xlsx) and images (EDFigure6_source_images) used in making Extended Data Fig. 6. [file 41593_2023_1260_MOESM12_ESM.zip › EDFigure6_source_images/EDFig6E_oscilloscope2.jpg]

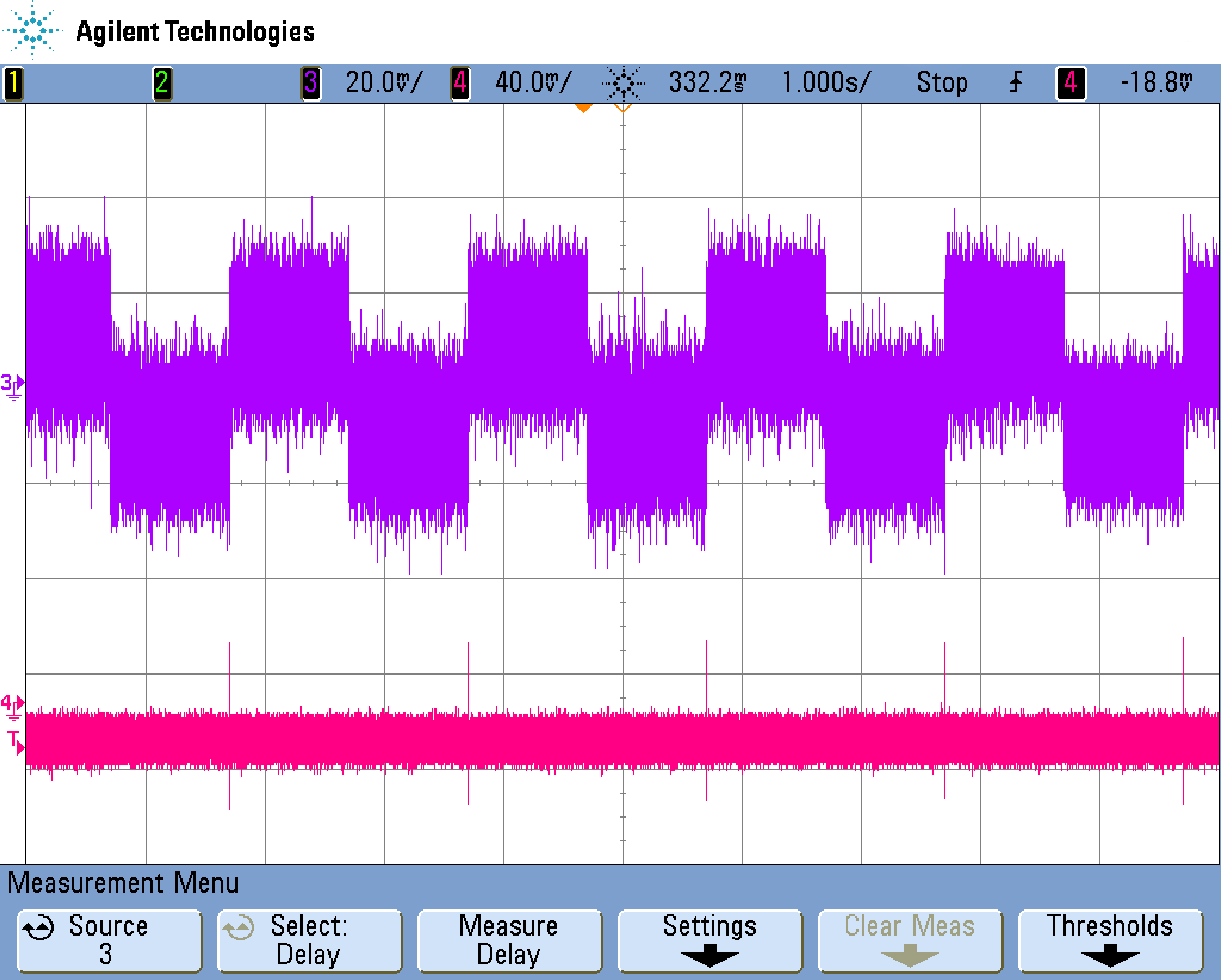

Supplement: Source Data Extended Data Fig. 6 — Contains data (EDFigure6_source_data.xlsx) and images (EDFigure6_source_images) used in making Extended Data Fig. 6. [file 41593_2023_1260_MOESM12_ESM.zip › EDFigure6_source_images/EDFig6D_oscilloscope1.jpg]

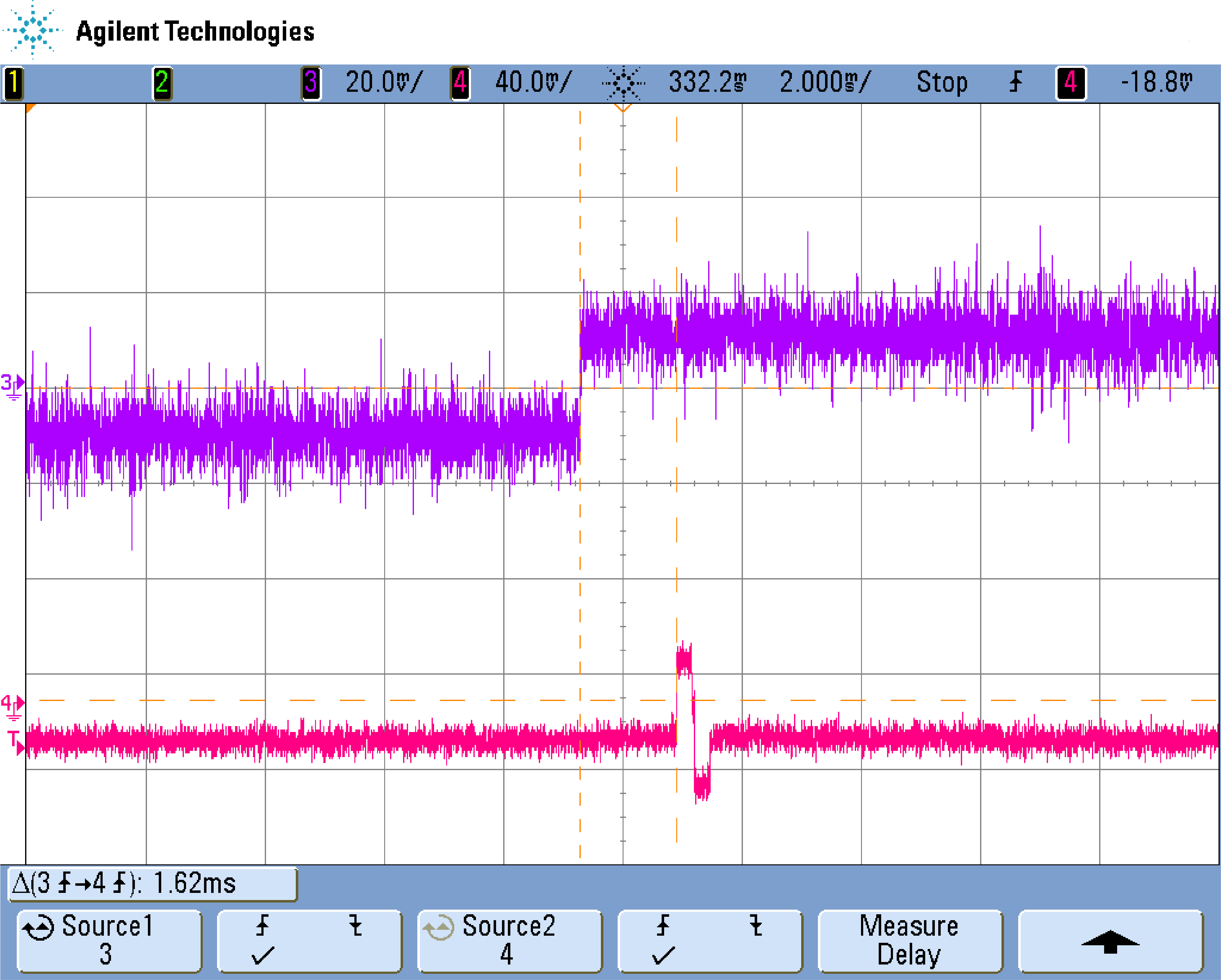

Supplement: Source Data Extended Data Fig. 6 — Contains data (EDFigure6_source_data.xlsx) and images (EDFigure6_source_images) used in making Extended Data Fig. 6. [file 41593_2023_1260_MOESM12_ESM.zip › EDFigure6_source_images/EDFig6D_oscilloscope2.jpg]
